# Evidence for a Grooming Claw in a North American Adapiform Primate: Implications for Anthropoid Origins

**DOI:** 10.1371/journal.pone.0029135

**Published:** 2012-01-10

**Authors:** Stephanie Maiolino, Doug M. Boyer, Jonathan I. Bloch, Christopher C. Gilbert, Joseph Groenke

**Affiliations:** 1 Stony Brook University, Stony Brook, New York, United States of America; 2 Brooklyn College, City University of New York, Brooklyn, New York, United States of America; 3 Florida Museum of Natural History, University of Florida, Gainesville, Florida, United States of America; 4 Hunter College, City University of New York, New York, New York, United States of America; 5 Stony Brook University, Stony Brook, New York, United States of America; University College London, United Kingdom

## Abstract

Among fossil primates, the Eocene adapiforms have been suggested as the closest relatives of living anthropoids (monkeys, apes, and humans). Central to this argument is the form of the second pedal digit. Extant strepsirrhines and tarsiers possess a grooming claw on this digit, while most anthropoids have a nail. While controversial, the possible presence of a nail in certain European adapiforms has been considered evidence for anthropoid affinities. Skeletons preserved well enough to test this idea have been lacking for North American adapiforms. Here, we document and quantitatively analyze, for the first time, a dentally associated skeleton of *Notharctus tenebrosus* from the early Eocene of Wyoming that preserves the complete bones of digit II in semi-articulation. Utilizing twelve shape variables, we compare the distal phalanges of *Notharctus tenebrosus* to those of extant primates that bear nails (n = 21), tegulae (n = 4), and grooming claws (n = 10), and those of non-primates that bear claws (n = 7). Quantitative analyses demonstrate that *Notharctus tenebrosus* possessed a grooming claw with a surprisingly well-developed apical tuft on its second pedal digit. The presence of a wide apical tuft on the pedal digit II of *Notharctus tenebrosus* may reflect intermediate morphology between a typical grooming claw and a nail, which is consistent with the recent hypothesis that loss of a grooming claw occurred in a clade containing adapiforms (e.g. *Darwinius masillae*) and anthropoids. However, a cladistic analysis including newly documented morphologies and thorough representation of characters acknowledged to have states constituting strepsirrhine, haplorhine, and anthropoid synapomorphies groups *Notharctus tenebrosus* and *Darwinius masillae* with extant strepsirrhines rather than haplorhines suggesting that the form of pedal digit II reflects substantial homoplasy during the course of early primate evolution.

## Introduction

The oldest fossil euprimates are known from the Late Paleocene-Eocene of Africa and the Eocene of North America, Europe, and Asia. There are two major radiations of early euprimates: the adapiforms and the omomyiforms. Adapiforms are a highly diverse group variously classified in a number of subfamilies: Notharctinae, Cercamoniinae, Caenopithecinae, Djebelemurinae, Asiadapinae, Adapinae, and Sivaladapinae. Notharctines are known exclusively from North America and include for example *Cantius*, *Notharctus*, and *Smilodectes*. Cercamoniinae has traditionally referred to many genera from Europe, North America, Asia, and Africa including *Europolemur*, *Donrussellia*, *Cercamonius*, *Caenopithecus*, *Mahgarita*, *Djebelemur*, *Aframonius*, and *Anchomomys* among others [1]. However, more recent analyses have suggested the separation of some into their own subfamilies: *Caenopithecus*, *Aframonius*, and *Mahgarita*, along with *Afradapis*, are considered caenopithecines by some [2], [3]. It has also been suggested that *Djebelemur* and ‘*Anchomomys*’ *milleri* are more closely related to strepsirrhine primates than other adapiform groups, and as such, this clade is sometimes referred to as the djebelemurines [2], [4], [5]. Notharctines, cercamoniines (including those genera that are sometimes distilled into other subfamilies), and another subfamily, the Asiadapinae, are usually grouped together in the family Notharctidae. Additionally, two other groups of adapiforms are recognized, the European and Asian Adapinae and the late surviving Sivaladapinae from Asia.

The relationship of adapiforms to living primate groups has always been a matter of debate. Recently, this debate has been re-kindled by the discovery of an exceptionally complete skeleton of the European cercamoniine *Darwinius masillae* [6]. Disagreement about the phylogenetic significance of its morphology highlights the need for more complete documentation of early euprimate anatomy [7] as well as a more quantitatively rigorous and broadly acceptable (by researchers with different philosophies on phylogeny reconstruction) analytical framework [8].

### Hypothesized relationships of adapiforms to extant primate clades

Two major hypothesized relationships between adapiforms and extant primates have been suggested. One position is that adapiforms share a special relationship with crown strepsirrhines as either a paraphyletic stem or monophyletic sister group e.g., [9], [10], [11], [12], [13], [14]. Alternatively, other researchers have suggested that adapiforms are more closely related to crown anthropoids e.g., [15], [16], [17], [18], [19], [20], [21], [22], [23], [24], [25].

A great number of shared similarities have been cited to link adapiforms and extant strepsirrhines. However, many of these similarities can be explained as plesiomorphic (primitive) primate characteristics [1], [26], [27]. These include the presence of a postorbital bar, an unfused mandibular symphysis (in the oldest and most primitive species, e.g. *Cantius* and *Donrussellia*), a ring-like ectotympanic within the petrosal bulla, a relatively long snout, and a median gap between the upper central incisors associated with wet-nose strepsirrhinism. However, several similarities in the postcranial morphology of adapiforms and strepsirrhines are absent in other primates as well as other eutherian mammals; these could be interpreted as shared-derived characteristics (synapomorphies) linking the two groups. These include a talus with a gently sloping talofibular-joint facet, a groove for the tendon of *M. flexor hallucis longus* that is positioned lateral to the posterior portion of the tibiotalar joint, and a large posterior trochlear shelf (though this is reduced in some adapines and the caenopithecine *Afradapis* [26]); a navicular on which the articular facet for the cuboid is contiguous with both those for the ectocuneiform and the mesocuneiform; and a strongly rotated medial malleolus of the tibia [10], [11], [12]. Results of some cladistic analyses using many taxa and large numbers of characters also support a strepsirrhine affinity for adapiforms [2], [28], [29], [30], [31], [32], [33].

In contrast, there are a number of features that have been suggested to be possible synapomorphies that link adapiforms, particularly cercamoniines, with anthropoids. These include similar skull shape; a fused mandibular symphysis (in some later occurring and more derived species, e.g., *Adapis, Notharctus* and *Darwinius*); a robust mandibular corpus; sexual dimorphism in canine size; “non-elongated” tarsals; an unfused tibia and fibula; and at least in *Mahgarita*, an enlarged promontory canal, pneumatized mastoid region, and the presence of a transverse septum that runs from the promontory canal to the lateral wall of the bulla [20], [23], [24]. Additionally, a number of features of the anterior teeth are shared between the two groups: an I_1_ that is relatively smaller than I_2_, short and vertical incisors with spatulate crowns but see [34], large and interlocking canines, an upper canine with a mesial groove, a canine honing facet on the anterior lower premolar, and heteromorphic anterior teeth [19], [23], [24]. Cladistic analyses have also been shown to support a close relationship between adapiforms and anthropoids rather than strepsirrhines [8], [20].

### *Darwinius* in the controversy

A remarkably complete skeleton of the cercamoniine adapiform, *Darwinius masillae*, from the Eocene of Grube Messel, Germany has recently been interpreted to provide strong evidence for an anthropoid affiliation of adapiforms [6], [8]. It was initially considered to be a haplorhine based on the presence of features considered to be key synapomorphies: a cranium with a short rostrum, a deep mandibular ramus, a fused mandibular symphysis (partial), vertical and spatulate incisors, a talus with a steep talofibular facet, and a loss of grooming claws [6]. It was further described as lacking several derived features of strepsirrhines: a tooth comb, a medio-laterally compressed mesocuneiform, and a pes with the fourth digit the longest [6], [8].

However, this interpretation has been highly controversial. Results from a cladistic analysis of 360 characters coded for a diversity of both extant and fossil taxa (n = 117; “many taxa” approach [8]) with a lot of missing data (53%), instead, suggest that *Darwinius* is part of a clade of caenopithecine adapiforms that (along with other adapiform taxa) is placed as a sister taxon to strepsirrhines [2] rather than haplorhines [6]. This result suggested that similarities shared between *Darwinius* (and other caenopithecines, like *Afradapis*) and haplorhines could be interpreted as the result of convergent evolution. Additionally, Williams et al. [7] noted that a short rostrum, symphyseal fusion, and a deep mandible have evolved in multiple euprimate lineages (both strepsirrhine and haplorhine), while vertical, spatulate incisors could be interpreted as primitive for euprimates. Complete symphyseal fusion is also absent in early haplorhines, including some early undisputed anthropoids from the Fayum [7], [34]. Williams et al. [7] further argue that a vertically oriented talofibular facet might also be primitive for euprimates; they interpret a vertical orientation in *Darwinius* as a secondary reversion to this condition, while maintaining that its presence in haplorhines is a primitive retention. They also suggest that the polarity of the absence of a grooming claw is unclear, and suggest that a nail on the second pedal digit might be the primitive primate condition.

Building on these ideas, Gingerich et al. [8] performed a cladistic analysis using a matrix of 30 characters coded for fewer primate taxa (n = 8), with much less missing data (6%). Results from this analysis suggested a special relationship between *Darwinius* and anthropoids specifically. Gingerich et al. [8] reinterpret a vertically oriented talofibular facet as a primitive euprimate trait, but maintain that a loss of grooming claws is an anthropoid synapomorphy. Two additional characters were also added as haplorhine synapomorphies: an uncompressed mesocuneiform and quadrate lower molars. Further, they criticize the use of matrices with large numbers of characters and many fragmentary fossil taxa, suggesting that character interdependence and large amounts of systematically distributed missing data might critically mislead such analyses.

Of particular interest in this debate, is the presence or absence of a grooming claw on the second pedal digit. Among fossil euprimates, a grooming claw has been described in one cercamoniine species, *Europolemur kelleri* [35], but is thought to be absent in the closely related *Europolemur koenigswaldi* [6], [36]. While a grooming claw has also been reported for *Notharctus* [37], it was based on an incomplete (missing distal end) element that, while part of a dentally associated skeleton, lacked any documented evidence of articulation with other digits. *Darwinius* was determined to lack a grooming claw, possessing “scutiform” distal phalanges on all digits [6]. We note that grooming claws, as well as all primate distal phalanges, possess an apical tuft (the mediolaterally flaring apron of bone at the tip of the terminal phalanx; presumably the “scutiform” morphology referred to by [6]). As such, the presence of an apical tuft does not necessarily indicate the absence of a grooming claw [38]. Rather, we recommend that a quantitative comparative approach is needed to best diagnose this feature in fossil euprimates. Furthermore, the presence of a grooming claw on the second pedal digit in some platyrrhines [1], [38], [39], [40], [41] suggests at least the possibility that this structure may have also been present in early anthropoids. However, relatively little is known about grooming claw evolution; no strong evidence refutes the possibility that it was acquired more than once by different primate groups.

Here, we report a newly discovered foot of *Notharctus tenebrosus*, an early to middle Eocene adapiform from Wyoming. The individual bones of this specimen were preserved in full to semi-articulation with each other. It is the first such specimen to be described with detailed documentation and analysis of its *in situ* context, and as such, it is the first specimen of a North American adapiform with verifiable attribution of phalanges to particular digit rays. Because of this context, we can now confidently identify the proximal, intermediate, and distal phalanges of the second and third digit rays, the proximal and distal phalanges of the fourth digit ray, and the proximal and intermediate phalanges of the fifth digit ray. To document the presence or absence of a grooming claw and examine the phenetic similarities of this specimen to extant primate taxa, we analyze the morphology of these digits, making comparisons with samples of fossil and extant primates (See Tables S1 and S2 for specimens in comparative sample and Table 1 for institutional abbreviations). Finally, we assess the phylogenetic significance of the new morphology by adding codings of *Notharctus* to an existing character matrix.

**Table 1:** Institutional abbreviations.

Abbreviation	Institution
AIZU	Anthropologisches Institut und Museum der Universität Zürich-Irchel, Zürich, Switzerland
AMNH	American Museum of Natural History, New York, NY
BMNH	British Museum of Natural History, London, England
BC	Brooklyn College, Brooklyn, NY
CMNH	Carnegie Museum of Natural History, Pittsburgh, PA
DUPC	Duke University Primate Center, Durham, NC
FMNH	The Field Museum of Natural History, Chicago IL
MCZH	Museum of Comparative Zoology (Harvard University), Cambridge, MA
MNHN	Muséum nationale d'Histoire naturelle, Paris
MNHU	Museum für Naturkunde der Humboldt Universität, Berlin, Germany
NMNH	National Museum of Natural History (Smithsonian Institution), Washington D.C.
RMNH	Rijksmuseum van Natuurlijke Historie, Leiden, The Netherlands
SBU	Stony Brook University, Stony Brook, NY
UNSM	University of Nebraska State Museum, Lincoln, NE

Abbreviations of institutions from which specimens were studied.

## Results

### Description of fossil specimens

#### Context and taxonomic attribution

**Figure 1:**
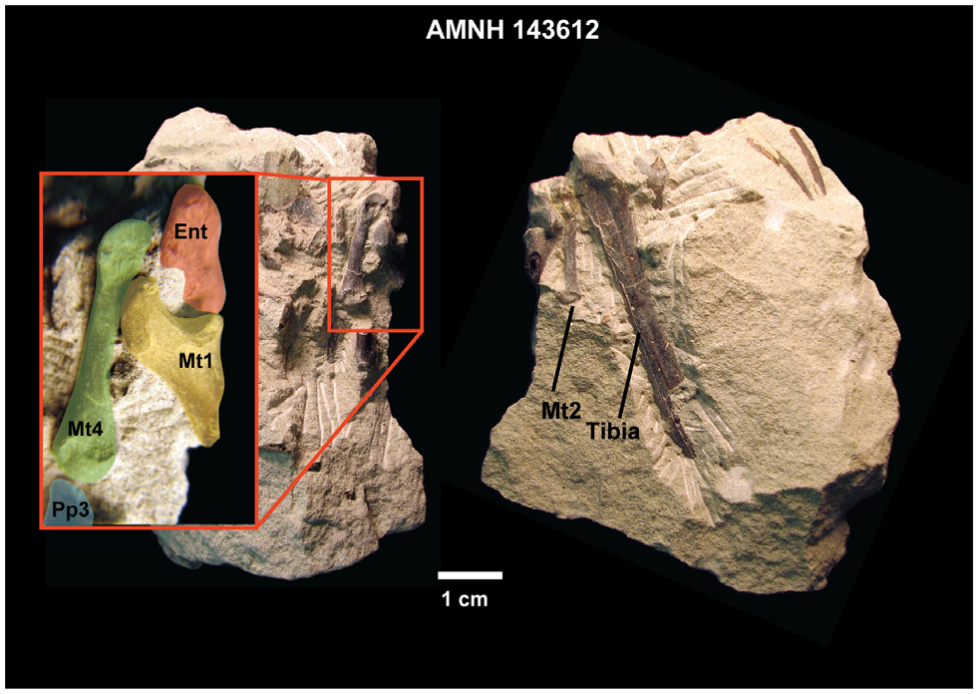
Original block containing new partial, semi-articulated foot of *Notharctus tenebrosus* AMNH 143612. Shown here as when found in collections at American Museum of Natural History. Two views are rotated 90 degrees around a vertical axis with respect to one another. Inset on left labels some of the bones visible on the surface, indicating potential for more below. Abbreviations: Ent, entocuneiform; Mt, metatarsal; pp, proximal phalanx. Numbers refer to digit rays.

**Figure 2:**
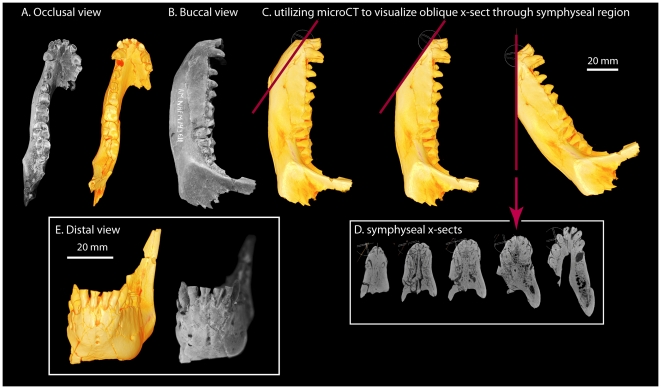
Mandible of *Notharctus tenebrosus* AMNH 143611 associated with AMNH 143612. Photographs are paired with voltex renderings generated from microCT scan data of this mandible. The scan was acquired at 0.05672105 millimeter resolution (cubic voxels) at the AMNH microCT scanning facility. A, Occlusal view. B, Buccal view. C, Buccal view of microCT rendering showing steps and positioning in preparation for viewing cross-sections through long-axis of mandibular symphysis. D, Series of cross-sections through mandibular symphysis, ranging from most ventral (left-most) to most dorsal (right-most), showing symphyseal fusion. E, Distal view showing spatulate nature of vertical incisors, and fused symphysis.

The blocks of sediment from which this study's focal specimens of *Notharctus tenebrosus* (AMNH 143612 and associated AMNH 143611) were prepared, were recovered from a cabinet in the fossil mammal collections at American Museum of Natural History. These materials are the results of collecting efforts by J. Alexander from 1990–2000 in the Bridger Formation of Grizzly Buttes (Bridger B), Bridger Basin, Wyoming [42], [43]. Before further preparation, one partially prepared block of AMNH 143612 revealed what were clearly semi-articulated elements of an adapiform foot (Fig. 1). Additional identifiable specimens in this accumulation include a partial femur and mandible (Fig. 2). The associated mandible (AMNH 143611) is readily identifiable as *Notharctus tenebrosus* on the basis of the size and shape of the teeth [I_1_ = 2.10 mm(mesiodistal length)×1.82 mm(buccolingual width); I_2_ = 2.44×2.38; C_1_ = 2.98×3.07; P_1_ = 2.05×2.08; P_2_ = 2.63×2.02; P_3_ = 3.62×2.56; P_4_ = 4.60×3.50; M_1_ = 5.34(mesiodistal length)×4.20(trigonid width)×4.76(talonid width); M_2_ = 5.88×4.77×5.21; M_3_ = 7.13×4.78×3.66] in addition to its stratigraphic horizon at Grizzly Buttes in Bridger B [44]. Like some other *Notharctus*, it exhibits vertical canines, spatulate incisors, and a fused mandibular symphysis (Fig. 2).

After beginning preparation of the hind foot elements entombed in AMNH 143612, another matrix block (AMNH 143640) from a different cabinet at the AMNH was scanned to assess its contents. To our surprise, it contained more foot material relatable to AMNH 143612 by the presence of re-attachable fragments of the first metatarsal and the digit I proximal phalanx of AMNH 143612. Additionally, the block preserved on its surface an impression of a non-hallucal proximal phalanx from AMNH 143612 (this was molded prior to preparation and is also visible in CT scans available on request from the authors). Elements of the fifth digit ray of the foot of AMNH 143612 were also present within AMNH 143640. Repositioning the part and counter part of the bone fragments and impressions allowed reconstruction of the positions of bones in one block with respect to the other.

#### Documentation of association of the pedal elements represented

**Figure 3:**
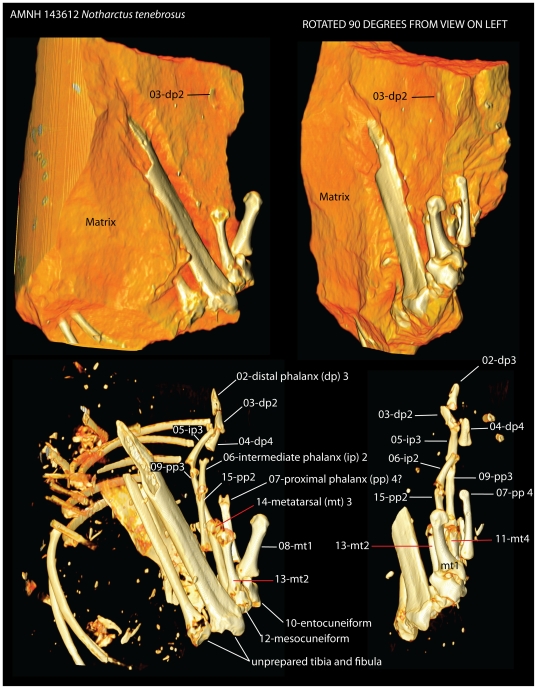
Labeled ct reconstruction of *in situ* elements. Left vs. right images are the same specimen rotated 90 degrees with respect to one another. Top vs. bottom images are rendered to show low density tufaceous matrix and to exclude it, respectively. Prior to preparation, all blocks catalogued as AMNH 143612 and AMNH 143640 were CT scanned at Stony Brook University Medical Center. The resulting images allowed us to determine which blocks contained pedal material and where it lay. Surprisingly, only two blocks (one depicted here, the same as in figure 1) contained identifiable foot material. As elements were removed, they were labeled with a unique number which is indicated for each bone visible in the bottom images. These numbers were recorded in a 3D pdf files containing images of the *in situ* bones, like that shown here, as physical preparation was undertaken (Appendix S2, S3). Only pedal elements have been physically removed at this time. Ribs, tibia, fibula, and fragments of an innominate remain embedded.

**Figure 4:**
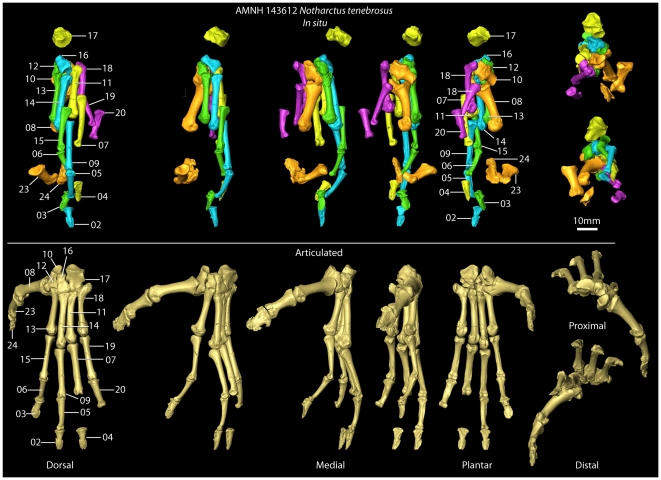
Pedal elements after preparation. After physical preparation of the foot was completed, all bones were scanned with microCT at resolutions ranging from 0.013–0.031 millimeter voxels. High resolution surface files were created from these images. One set of images was overlaid on the original CT scan shown in Fig. 3 to allow easier viewing and study of the *in situ* elements (top row). Another set of 3D surface images were articulated in a “closest packed” arrangement to get a better sense of what the foot looked like in the living animal (bottom row).

Based on 3D virtual representations of the preserved foot bones (Figs. 3, 4), it is clear that some disarticulation occurred shortly after burial (but before lithification). The movement of bones appears patterned, in that certain sets of elements have been similarly displaced relative to other sets; this patterning allows for confident reconstruction of original positions of displaced bones.

Bones that remained essentially fully articulated, as in the living animal, include the ectocuneiform; mesocuneiform; entocuneiform; and first, second and third metatarsals. The cuboid was rotated and shifted proximally out of articulation. Likewise, the fourth metatarsal, although not dramatically displaced, was also shifted proximally. Its proximal end was additionally moved medially, so that it touched the lateral surface of the entocuneiform. Finally, the plantar surface of the fourth metatarsal's distal end was rotated so that it faced more laterally. The fifth metatarsal was shifted both proximally and medially so that its peroneal process almost touched the plantar surface of the entocuneiform. Two sets of proximal and intermediate phalanges remained directly articulated during preservation. The more medial pair (#06 and #15; Figs. 3, 4) was shifted proximally and dorsally relative to the distal ends of the metatarsals. Additionally, this pair seems to have moved laterally until its progress was stopped by abutting with the adjacent lateral digit (#05 and #09; Figs. 3, 4), which appears to be in articulation with the third metatarsal (but see below). A third proximal phalanx (#07; Figs. 3, 4), that lacks an articulating intermediate phalanx, was shifted farther proximally than the other two, was rotated more and slipped to that plantar side of the foot. The distal phalanges are in close proximity to the tips of the intermediate phalanges #05–06 (Figs. 3, 4). They seemed to have shifted distally and thereby pulled out of articulation with their corresponding intermediate phalanges. Another set of proximal (#19) and intermediate (#20; Figs. 3, 4) phalanges was clearly associated with the fifth metatarsal (these are elements from AMNH 143640). This proximal phalanx (like those for other digit rays) was shifted proximally and laterally. In this case the intermediate phalanx has been similarly shifted with respect to the proximal phalanx (Fig. 4). Finally, the proximal and distal phalanges of the first digit are touching and, together, shifted distally away from their original contact with the first metatarsal (Fig. 4).

Images of the bones *in situ* from approximate dorsal views (Fig. 4: labeled images on top row) show that the distal phalanx with the most proximal position is also the most laterally positioned of the three (#04). The distal phalanx with the second most proximal position is the most medial of the three elements (#03). Finally the most distally positioned distal phalanx is in between the other two (#02). This matches the pattern of positions of the proximal phalanges with the most lateral (#07) being most proximally positioned, followed by the most medial (#15) and finally by the phalanx positioned in between the other two (#09), which has the most distal position.

**Figure 5:**
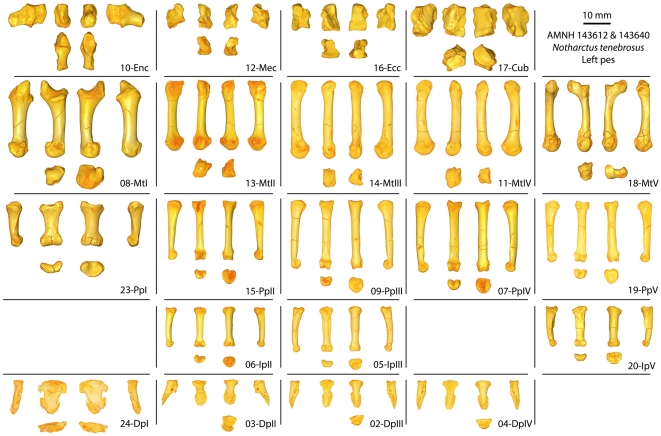
Standard views of all preserved pedal elements for AMNH 143612 and AMNH 143640. Standard views were captured in Avizo 6.3 as represented using the voltex view feature. Top rows of each element, from left to right depict lateral, plantar/volar, dorsal, and medial views. Bottom rows depict distal on the left and proximal on the right. Left most column shows bones of digit ray I, followed by ray II to the right, and so on.

Also of note, is the fact that the most medially positioned proximal, intermediate, and distal phalanges (#15, #06, and #03; Fig. 5) are shorter and have narrower articular surfaces than the more laterally positioned serial homologues. Comparing phalanges #09, #05, and #02 (those occurring just lateral to the most medial set mentioned above) to the next, more lateral proximal (#07) and distal (#04) phalanges, it is apparent that the latter two have greater length and breadth dimensions (Table 2; Fig. 5). Finally the proximal and intermediate phalanges that are most laterally positioned (#19, #20) and closest to the fifth metatarsal have decreased lengths, but similar widths, as compared to more medial proximal (#07, #09) and intermediate (#05) phalanges (Table 2; Fig. 5). Minimally, the stated observations suggest that bones #15, #06, and #03 belong to a single ray; that #09, #05, and #02 belong to a single, laterally adjacent ray, that #07 and #04 were a part of the next more lateral ray, and that #19 and #20 were part of a more lateral ray yet. Accepting this interpretation, it appears that bones #15, #06 and #03 belong to the second ray; #09, #05, and #02 belong to the third ray; #07 and #04 belong to the fourth ray; and #19 and #20 belong to the fifth. While other possible interpretations exist, they are much more complex in terms of the disarticulation movements that must have occurred and are excluded from consideration for that reason.

**Table 2:** Basic measurements of AMNH 143612 & 143640, *Notharctus tenebrosus*.

Specimen	Element^a^	L^b^	PEW^c^	PED^c^	MSW	MSD	DEW^d^	DED^d^
AMNH 143612-10	Enc	9.33	4.20	6.65	na*	na	5.57	7.71
AMNH 143612-12	Mec	6.60	5.16	6.63	na	na	3.56	5.91
AMNH 143612-16	Ecc	9.62	6.51	6.63	na	na	4.27	5.24
AMNH 143612-17	Cub	11.34	8.69	5.67	na	na	7.28	∼5.71
AMNH 143612-08	Mt1	25.56	10.11	8.90	4.51	5.04	nm**	6.74
AMNH 143612-13	Mt2	25.56	4.82	6.66	3.05	3.28	5.42	7.06
AMNH 143612-14	Mt3	28.44	5.42	5.73	3.31	3.34	4.75	6.88
AMNH 143612-11	Mt4	27.46	4.64	5.68	3.51	3.48	5.28	7.74
AMNH 143640-18	Mt5	24.53	8.45	5.22	2.91	2.19	5.41	6.73
AMNH 143612-23	Pp1	16.02	8.86	5.86	4.83	3.50	8.14	3.83
AMNH 143612-15	Pp2	21.49	5.07	5.22	2.43	2.86	4.24	3.53
AMNH 143612-09	Pp3	25.16	5.21	5.39	3.01	2.70	4.38	3.61
AMNH 143612-07	Pp4	25.98	5.53	5.50	3.29	2.83	4.86	3.80
AMNH 143640-19	Pp5	22.41	5.35	5.37	3.03	2.73	4.84	3.58
AMNH 143612-06	Ip2	14.47	4.60	4.01	2.49	2.26	4.20	2.91
AMNH 143612-05	Ip3	16.43	4.83	4.02	3.13	2.33	4.26	2.64
AMNH 143640-20	Ip5	15.14	5.73	4.58	3.58	2.61	4.50	2.68

Measurements (in millimeters) of AMNH 143612 and 143640 elements.

a Element abbreviations: Cub, cuboid; Ecc, ectocuneiform; Enc, entocuneiform; Ip, intermediate phalanx; Mec, mesocuneiform; Mt, metatarsal; Pp, proximal phalanx.

b Measurement abbreviations: DED, distal end dorsovolar depth; DEW, distal end mediolateral width; L, proximodistal length; MSD, midshaft dorsovolar depth; MSW, midshaft mediolateral width; PED, proximal end dorsovolar depth; PEW, proximal end mediolateral width.

c For elements Enc and Cub, these measurements refer specifically to the dimensions of navicular facet and calcaneal facet, respectively.

d For elements Enc and Cub, these measurements refer specifically to the dimensions of the Mt1 facet and Mt4-5 facet, respectively.

*Measurement not applicable.

**Measurement not obtainable due to breakage.

On an initial pass, a more complex scenario might be suggested by the fact that proximal phalanx #09 appears to be in contact with the fourth metatarsal. However, this would then require positing #07 as belonging to the fifth digit, which would present an unusual set of proportions for this foot. First, it would imply that the fifth proximal phalanx is longer than the fourth proximal phalanx. In an extant sample of 285 individual primates (39 species), not a single specimen exhibits such proportions. This interpretation would then require that bones #19–20 be attributed to the second digit. This is problematic as they are quite distant from the second metatarsal, and it would present another strange pattern in which the phalanges of digit 3 (#15, #06, #03 under this interpretation) were shorter and narrower than those of digit 2 and 4.

What seems most likely to us is that the four sets of non-hallucal phalanges have essentially kept their correct anatomical positions relative to each other and the hallucal proximal phalanx, but have all been shifted proximally and laterally to varying degrees relative to the metatarsals. The second digit also shifted dorsally, while the fourth and fifth digits had plantar components to their trajectory. The third digit was neutral with respect to dorsoplantar deviations, thus its trajectory drew it from the more distal and medial position of the third metatarsal head to the more proximal and lateral position of the fourth metatarsal head, thereby presenting a “false” articulation. This interpretation is implemented in the analyses presented below.

#### Morphological description of AMNH 143612 and 143640

The tarsal and metatarsal elements (Fig. 5) are very similar to those previously described for *Notharctus tenebrosus* e.g., [9], [45] and are not re-described at this time. However, it is worth noting that the mesocuneiform is narrower and smaller than the ectocuneiform [8]. Additionally, when articulated, a plantar process of the proximal aspect of the mesocuneiform wraps laterally under the ectocuneiform and contacts the cuboid in most foot positions (Fig. 4), probably with the exception of an extremely inverted foot posture. This configuration implies a navicular exhibiting a cuboid facet that touches the mesocuneiform facet as well as the ectocuneiform facet e.g., [10].

The new partial skeleton of *Notharctus tenebrosus* described here (AMNH 143612, 143640) is exceptional in allowing description of nearly all phalanges (excepting the fourth intermediate and the fifth distal) for the first time. Compared to the other non-hallucal proximal phalanges, the proximal phalanx of the second digit (pp2) has: (1) the shortest length (Fig. 5; Table 2), (2) a shaft that is narrower mediolaterally, and deeper dorsplantarly, (3) flexor sheath ridges that are positioned more proximally and are less well-developed, (4) a smaller proximal end with a more restricted lateroplantar tubercle, and (5) distal condyles with a more pronounced asymmetry that is reversed (the lateral condyle projects farther than the medial). In contrast, compared to the other non-hallucal proximal phalanges, the proximal phalanx of the fourth digit (pp4) is the longest, most robust, and has a shaft that is more curved (as observable in a lateral view of Fig. 5). The pp4 is most similar to the proximal phalanx of the fifth digit (pp5) as, when compared to the other non-hallucal proximal phalanges, they both have: (1) flexor sheath ridges that flare more prominently, (2) a medioplantar tubercle on the proximal end that is more strongly developed, and (3) distal condyles that have lower degrees of asymmetry. Finally, compared to the non-hallucal proximal phalanges, the hallucal proximal phalanx (pp1): (1) is much shorter and mediolaterally broader, (2) has a much smaller lateroplantar process compared to the strongly proximally projecting medioplantar process, and (3) has a distal articular surface that is wide and flat, more like the condition of the intermediate phalanges.

The differences between the medial two preserved intermediate phalanges are in some ways similar to those exhibited by the proximal phalanges of the same digit rays (Fig. 5; Table 2). Compared to the intermediate phalanx of the third digit (ip3), the intermediate phalanx of the second digit (ip2): (1) is shorter and narrower, (2) has less-developed flexor sheath ridges (and flexor tendon attachments), and (3) has reduced shaft curvature. The shape and degree of asymmetry in the distal condyles appears to be similar in ip2 and ip3, with the lateral condyles projecting more plantarly than the medial condyles. The intermediate phalanx of the fifth digit (ip5) is unique among the three preserved in having: (1) fairly symmetrical distal condyles, (2) the highest degree of shaft curvature, (3) the strongest flexor sheath ridges (especially that on the medial side), (4) the most robust shaft proportions, and (5) a strongly asymmetrical proximal end that faces laterally, suggesting that ip5 projects laterally when articulated with pp5. Finally, ip5 is longer than ip2, but shorter than ip3.

**Figure 6:**
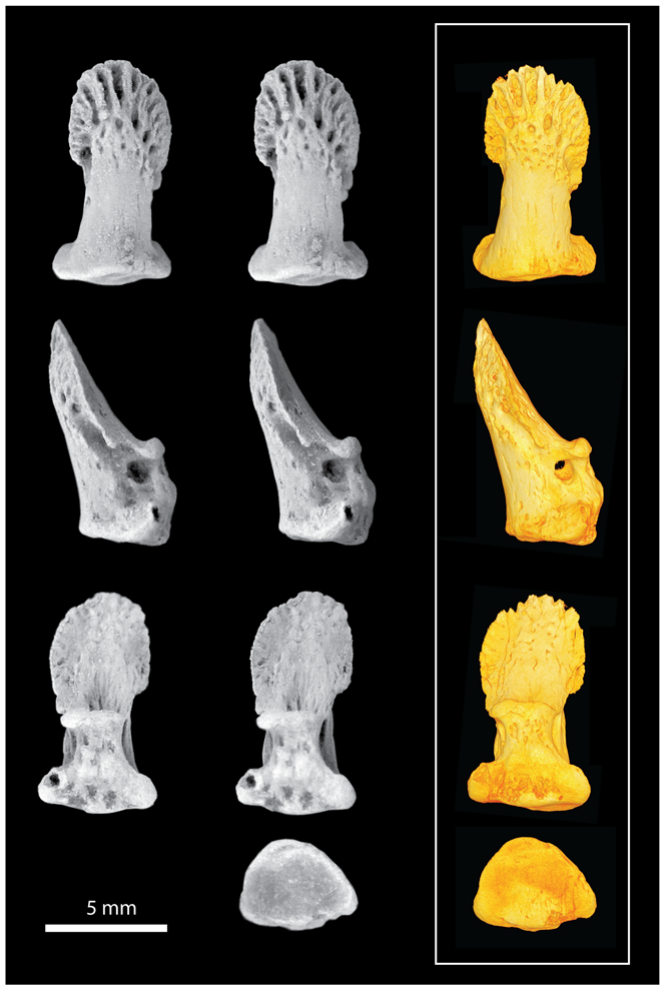
*Notharctus tenebrosus* AMNH 143612-03, pedal distal phalanx of digit two. Views are dorsal (top row), medial (second row), ventral (third row), and proximal (bottom row). Left two images are stereopair photographs. Right side images are virtual reconstructions from a microCT scan taken at 0.013 mm resolution.

**Figure 7:**
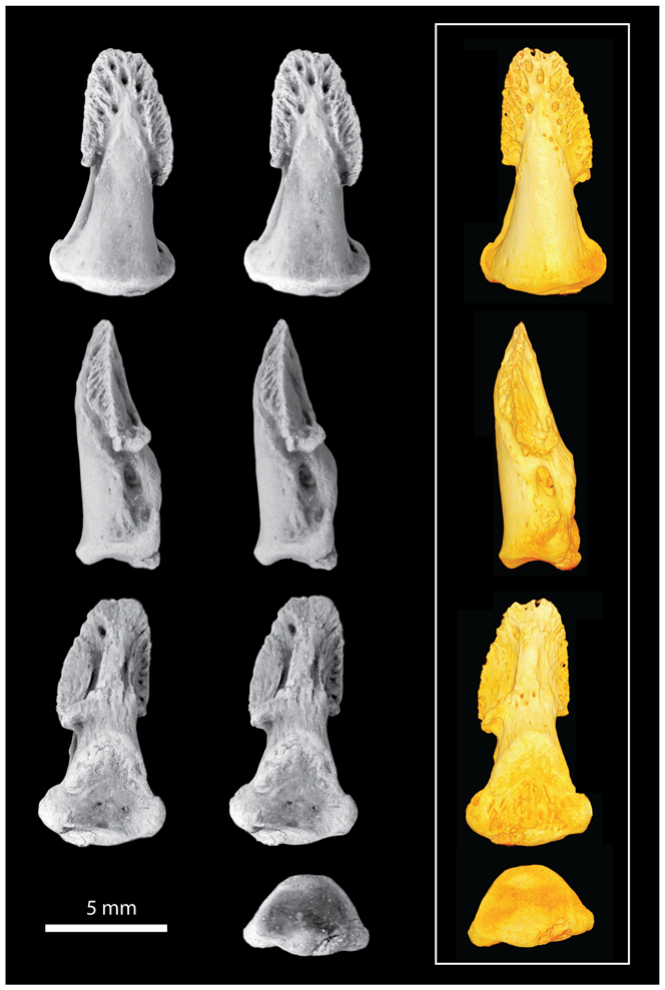
*Notharctus tenebrosus* AMNH 143612-02, pedal distal phalanx of digit three. Views are dorsal (top row), medial (second row), ventral (third row), and proximal (bottom row). Left two images are stereopair photographs. Right side images are virtual reconstructions from a microCT scan taken at 0.013 mm resolution.

**Figure 8:**
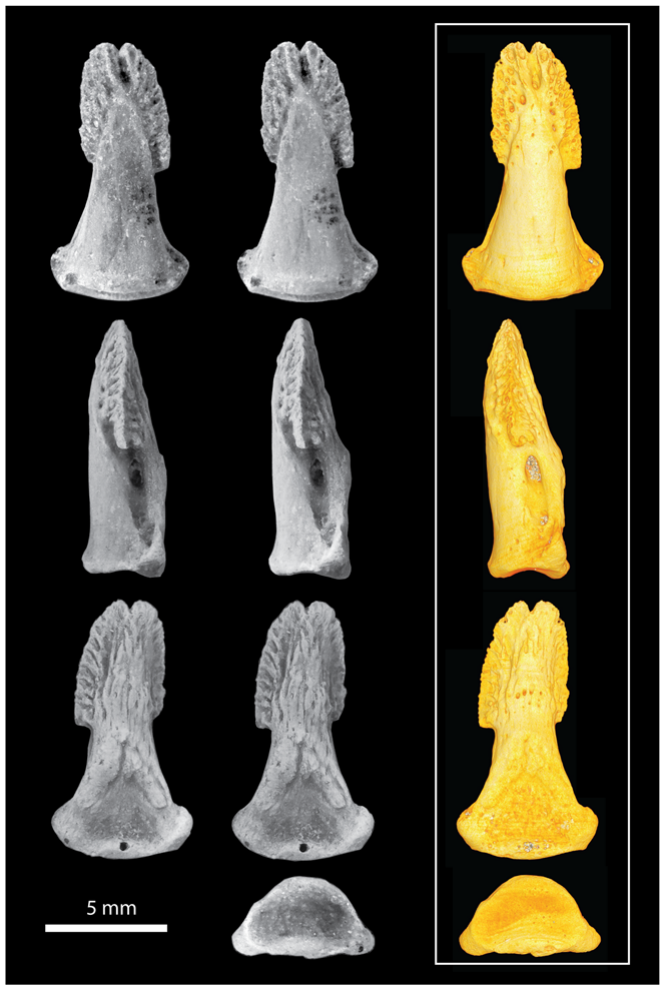
*Notharctus tenebrosus* AMNH 143612-04, pedal distal phalanx of digit four. Views are dorsal (top row), medial (second row), ventral (third row), and proximal (bottom row). Left two images are stereopair photographs. Right side images are virtual reconstructions from a microCT scan taken at 0.013 mm resolution.

**Figure 9:**
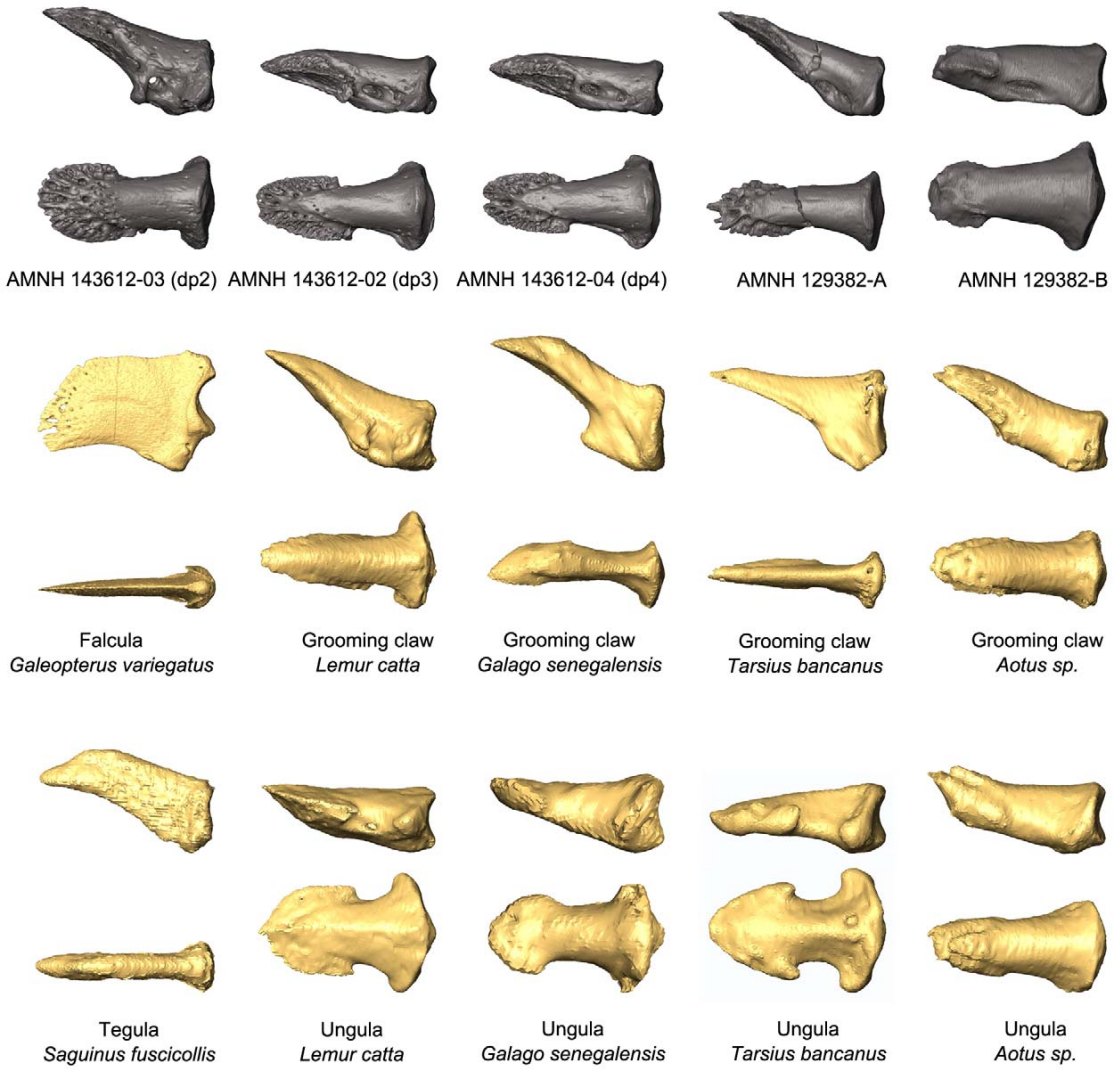
Comparison of fossil and extant distal phalanges. MicroCT images of distal phalanges are displayed in two views: lateral (above) and dorsal (below). Fossil unguals are shown in comparison to extant specimens that bear different unguis forms: falculae (claws), grooming claws, tegulae, and ungulae (nails).

The distal phalanges provide the most important new information available in AMNH 143612 (Figs. 5, 6, 7, 8, 9; Table 3). As discussed above, these can be confidently associated with pedal digits one, two, three, and four. All have well-developed apical tufts and appear as ungulae (not claws or falculae) in dorsal view. Compared to the non-hallucal distal phalanges, the hallucal distal phalanx (dp1) is much larger, wider, and flatter (Table 3) with a shaft that is more asymmetrical and deviates strongly laterally. In the three non-hallucal elements, the tufts are asymmetrical and point slightly laterally. In lateral view, the dorsal margins exhibit greater convexity than the volar margins, giving the bones a more “claw-like” appearance. Finally, like dp1, all exhibit massive, paired proximal nutrient foramina which lead to a proliferation of vascular channels in the bone of the apical tuft (Figs. 5, 6, 7, 8, 9). Compared to the other non-hallucal distal phalanges, that attributed to the second digit (dp2) is the most distinct in many features: (1) its shaft and tuft are strongly dorsally inclined relative to the proximal articular surface (see below), (2) the area of attachment for the flexor tendon (a convex, v-shaped tuberosity which forms the distal margin of a deep concavity) is positioned more proximally and is less extensive, (3) the maximum distal extent of the volar process is reduced, (4) the length of the shaft is absolutely less, (5) the breadth of the apical tuft is absolutely greater, (6) the breadth of the proximal end is absolutely smaller, while its height is absolutely greater, (7) in dorsal view the shaft deviates slightly medially relative to the proximal articular surface (in the opposite direction from the flaring tuft), (8) it has a prominent, ridge-like process that runs medio-laterally at the distal margin of the volar process and flares medially to end in a prominent tubercle (Fig. 6), and (9) the plantar surface, beyond the volar process of dp2 is relatively flat, rather than mediolaterally convex. The distal phalanx of the third digit (dp3; Fig. 7) is in most respects similar to that of the fourth (dp4), but is slightly smaller, has a shaft that projects slightly more dorsally, a volar process that is slightly more proximally restricted, and a proximal articular surface that is slightly dorsoventrally deeper relative to its mediolateral width. Like dp2, dp3 possesses a similar ridge-like process on its volar surface, but differs in that it is positioned at the proximal extent of the apical tuft, is less pronounced, and runs at a more oblique angle. This feature is absent in dp4, and is positioned and structured differently in dp2 and dp3, so its significance is difficult to interpret. Further, its presence may simply be pathological. Compared to the other non-hallucal distal phalanges, dp4 (Fig. 8) is: (1) the largest, (2) has a shaft that projects more distally and less dorsally, (3) a volar process that is more distally extended, and (4) a proximal articular surface that is widest relative to its dorsoplantar depth. Many of these features are put into a quantitative comparative context in the following sections.

**Table 3:** Basic measurements of *Notharctus* distal phalanges.

Specimen	TPL^b^	BH	BW	VFL	SW¾	FSA	VFL/TPL	(SH-¼)/SH(-¾)	(SW-¾)/TPL
11474^a^	8.15	2.45	3.80	3.69	2.10	83.38	0.45	1.57	0.26
129382-A	9.15	2.95	4.02	3.12	2.98	61.05	0.34	1.88	0.33
129382-B	6.40	2.48	3.95	4.01	2.13	80.60	0.63	1.28	0.33
143640-24 (dp1)	13.36	3.65	10.53	12.09	6.24	72.94	0.90	1.04	0.47
143612-03 (dp2)	10.64	3.94	5.21	4.38	4.18	56.87	0.41	2.33	0.39
143612-02 (dp3)	10.71	3.49	5.35	4.79	3.25	76.88	0.45	1.64	0.30
143612-04 (dp4)	11.05	3.65	5.96	5.30	3.42	77.97	0.48	1.57	0.31

Measurements from *Notharctus tenebrosus* pedal distal phalanges held at the American Museum of Natural History. TPL, BH, BW, VFL, and SW-3/4 are in millimeters; FSA is in degrees; and VFL/TPL, (SH-1/4)/(SH-3/4), and (SW-3/4)/TPL are dimensionless indices. See Materials and Methods for details on measurements.

a All specimen numbers are from AMNH.

b Measurements: TPL, total proximodistal length of phalanx; BH, dorsovolar height of phalanx base; BW, mediolateral width of phalanx base; FSA, angle between plane of proximal articular facet and proximodistal axis of shaft; SH¼, dorsovolar height of shaft measured at a distance of ¼ of the total shaft length from the proximal end; SH¾, dorsovolar height of shaft measured at a distance of ¾ of the total shaft length from the proximal end; SW¾, mediolateral width of shaft at ¾ TPL length as measured from the proximal end; VFL, Proximodistal distance between proximal end and distal-most extent of volar process.

#### Description of other distal phalanges from Bridger Basin Notharctus

We include in this study two additional, previously undescribed, distal phalanges of *Notharctus tenebrosus* (AMNH 129382). These phalanges are pedally-associated (though not with specific rays). They are similar to one another in having apical tufts that are well-developed, dorsal margins that are more convex in lateral view than the volar margins, and massive paired proximal nutrient foramina leading to a proliferation of vascular channels in the apical tuft (Fig. 9). Otherwise, these two bones are very different from each other. Compared to distal phalanx ‘B’ (Table 3), distal phalanx ‘A’ (Fig. 9): (1) is much larger, (2) has a shaft that is relatively narrower and dorsally inclined, (3) has a more proximally placed flexor tendon attachment, (4) a volar process that is much more proximally restricted, and (5) exhibits little asymmetry (‘B’ has a shaft that projects to the left relative to its proximal articular surface in dorsal view).

### Comparisons

#### Comparisons to feet of other *Notharctus*

Gregory [9] published measurements of metatarsals for two specimens of *Notharctus*: AMNH 11474 *Notharctus osborni* and AMNH 11478 *Notharctus tyrannus* (both now considered synonyms of *Notharctus tenebrosus*) [44]. The new specimen (AMNH 143612, 143640) appears to have relatively larger feet, with its non-hallucal metatarsals ranging in length from 111–144% of those of AMNH 11474 and 11478. It also has different metatarsal proportions with metatarsal 2 measuring 90% the length of metatarsal 3 (they have the same length in both AMNH 11474 and 11478). The preserved pes-associated proximal and intermediate phalanges of AMNH 11474 do not differ substantially from those attributed to the third and fourth digits of AMNH 143612, although (like the metatarsals) the phalanges of AMNH 11474 are also smaller than those of AMNH 143612. Likewise, a single complete pes-associated non-hallucal distal phalanx of AMNH 11474 is essentially similar to the distal phalanges attributed to the third and fourth digits of AMNH 143612, with several exceptions: its apical tuft appears slightly narrower (Table 3), it lacks any development of a ridge and tubercle on its volar process or at the proximal extent of its apical tuft, it is absolutely smaller, and its shaft and tuft seem more symmetrical.

The distal phalanges of AMNH 129382 differ from those of AMNH 143612 mainly in being absolutely smaller, relatively narrower, and lacking development of a ridge and tubercle on its volar surface as on dp2–3 of AMNH 143612. AMNH 129382-A is most similar to dp2 of AMNH143612 in the inclination of its shaft, convex form the attachment for the flexor tendon, and proximal restriction of its volar process. AMNH 129382-B is more similar to dp3–4 of AMNH 143612 in these and other features.

#### Comparisons of distal phalanx shape among extant and fossil euprimates

**Figure 10:**
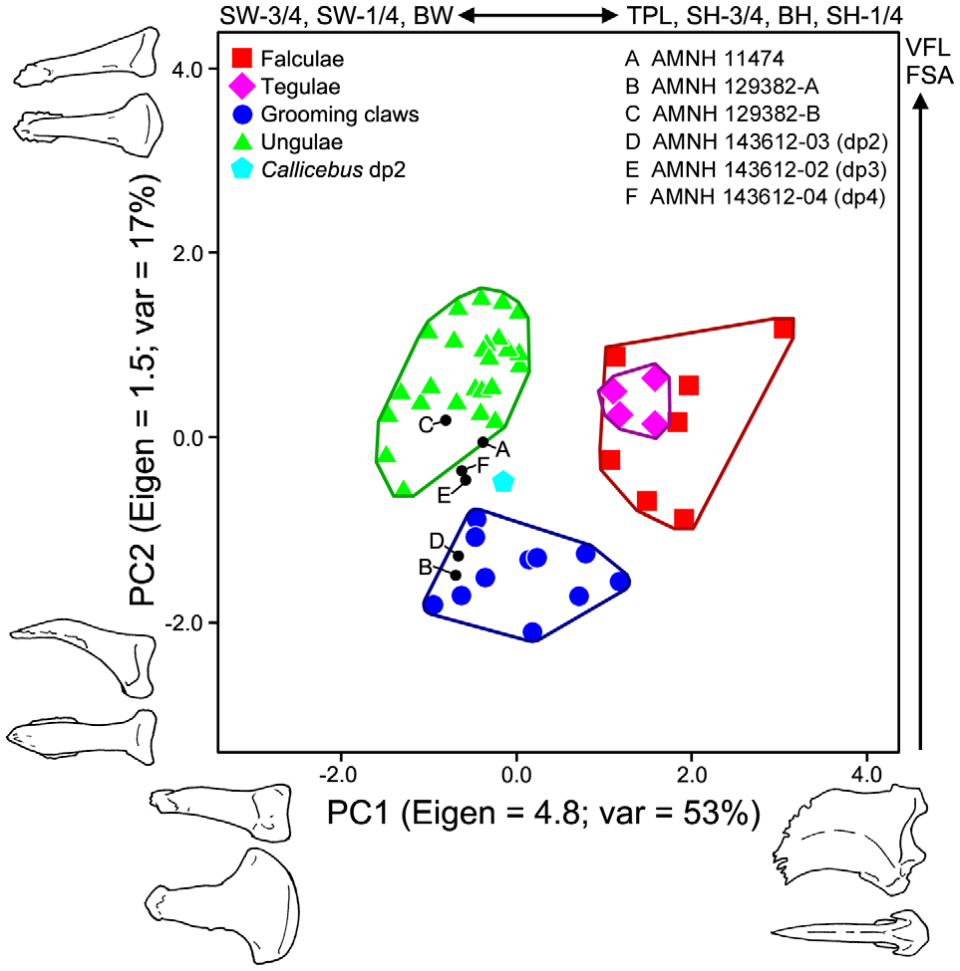
Principle components analysis of distal phalanx morphology. The first two components of a principal components analysis of ungual morphology are plotted. *Notharctus tenebrosus* specimens are represented by black dots. Specimens illustrated along the axes represent the extreme points along each axis: x axis, *Nycticebus coucang* (dp2) and *Galeopterus variegatus*; y axis, *Nycticebus coucang* (dp3) and *Hylobates sp.* Variables which are most strongly correlated with each component are also listed along the axes. See Table 3 and Materials and Methods for abbreviations and measurement descriptions.

**Figure 11:**
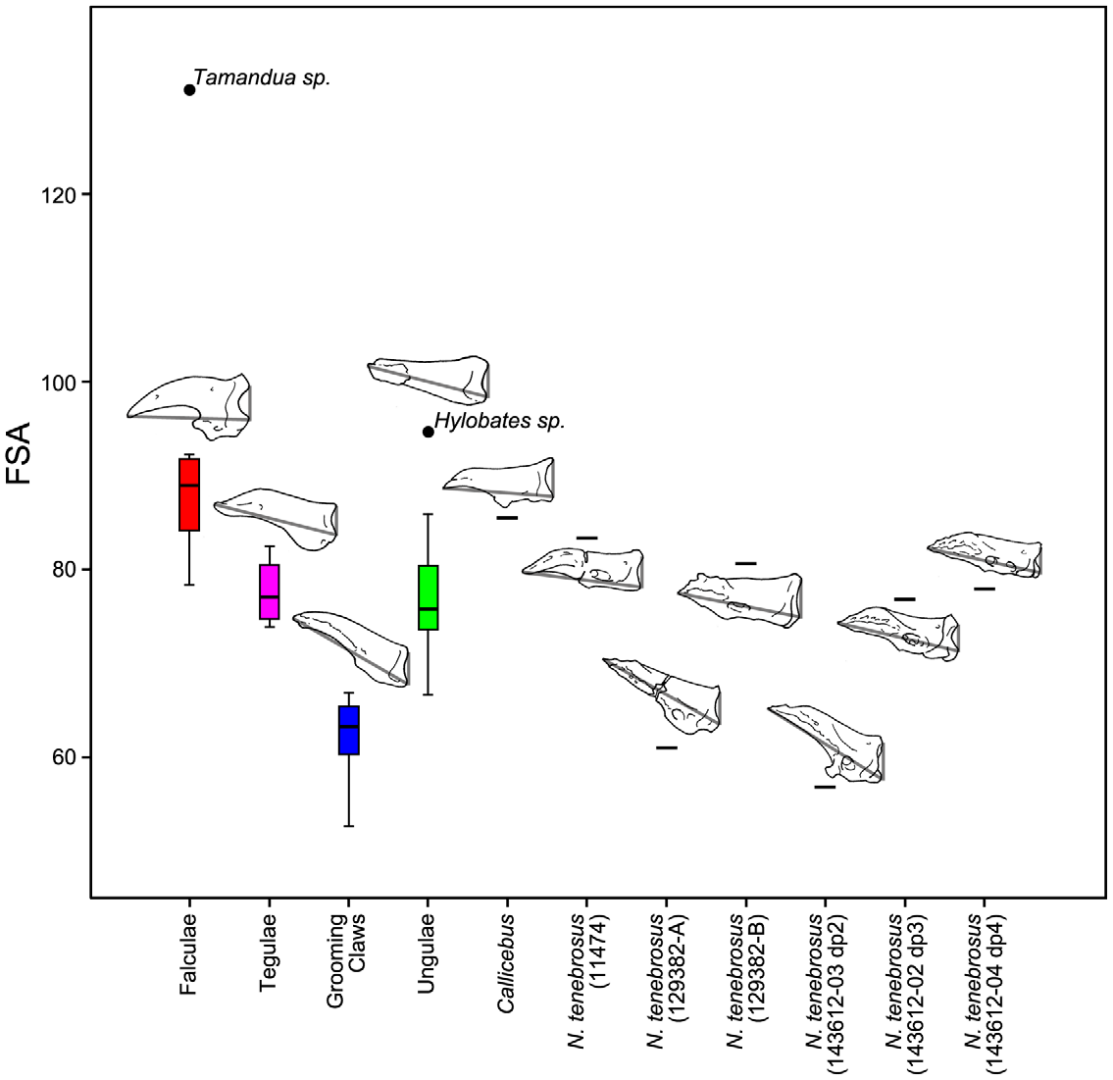
Boxplot of angle formed between distal phalanx proximal articular facet and shaft. Facet-Shaft Angle (FSA, the angle between the two segments) is demonstrated using illustrations of specimens scaled to the same length and oriented such that the superior and inferior margins of the articular facet are within the same plane. The median specimen from each extant group is illustrated: *Suricata suricatta* represents the falculae group; *Callithrix sp.*, tegulae; *Hapalemur griseus*, grooming claws; and *Galago senegalensis*, ungulae. See Materials and Methods for measurement description.

A principal component analysis (PCA), MANOVA/ANOVA, and *t*-tests were performed on size-adjusted shape and angular variables from distal phalanges of extant and fossil species to quantify and diagnose grooming claw morphology (see Materials and Methods for sample and group inclusions, and for variable definitions). The first two components of the PCA account for 70% of the total variance (Fig. 10). The first component has an eigenvalue of 4.8 and accounts for 53% of the variance while the second component has an eigenvalue of 1.5 and accounts for 17%. See Table 4 for component loadings. Ungular phalanges (phalanges that bear ungulae or nails) and grooming phalanges (phalanges that bear grooming claws) of extant primates are well separated from one another along component 2. In particular, the variables facet-shaft angle (FSA) and volar feature length (VFL) are most strongly correlated with component 2 showing that the shafts of grooming phalanges are dorsally canted with respect to their proximal articular facets (low values of FSA) and have shorter volar processes (lower VFL, a measure of the portion of the phalanx which supports the apical pad) in comparison to ungular phalanges. Component 1 separates ungular and grooming phalanges from falcular (phalanges of non-primate mammals that bear falculae or claws) and tegular (phalanges of callitrichine primates that bear claw-like tegulae) phalanges. Two *Notharctus* specimens [AMNH 129382-A and AMNH 143612-03 (dp2)] fall within the convex hull defined by measurements from the grooming phalanges of extant primates. AMNH 129382-B falls within the convex hull defined by those from ungular phalanges of extant primates and AMNH 11474 falls right on its edge. The shape of AMNH 143612-02 (dp3) and 04 (dp4), along with that of the second pedal phalanx of *Callicebus* (see Materials and Methods), is between that of the extant ungular and grooming phalanges. The MANOVA shows that there are significant differences among unguis-form groups (p<0.001), and more specifically, a post hoc Hotelling's pairwise comparison shows that there is a significant difference between extant grooming and ungular groups (p<0.001). A series of ANOVAs show that there are significant differences among unguis forms for all variables (p<0.001; Table 5). However, post hoc Tamhane's T2 tests show significant differences (significance is assessed at the Bonferroni adjusted alpha of 0.0042) between the ungular and grooming phalanx groups for total phalanx length (TPL), FSA, and VFL (p<0.0042), but not for any of the measurements of width (base width [BW], width of the proximal portion of the shaft [SW-1/4], and width of the distal portion of the shaft [SW-3/4]) or for base height (BH) and height of the proximal portion of the shaft (SH-1/4; p>0.0042). Height at the distal portion of the shaft (SH-3/4) was not significant at the Bonferroni adjusted value, but still had a low p-value (p<0.008). These analyses demonstrate that grooming phalanges are dorsally canted (low FSA; Fig. 11), have shorter volar features (low VFL), and are relatively longer when compared to ungular phalanges. The volar feature is associated with the extent of the apical pad along the volar surface of the phalanx; thus short volar features of the grooming phalanges indicate that the shaft of the phalanx projects far beyond the apical pad.

**Table 4:** Principal component loadings from an analysis of distal phalanx shape.

Variable	PC 1	PC 2
BH/GM	0.88	−0.30
BW/GM	−0.72	−0.38
TPL/GM	0.67	−0.47
SH-¼/GM	0.89	−0.15*
SW-¼/GM	−0.83	−0.15*
SH-¾/GM	0.75	0.43
SW-¾/GM	−0.83	−0.11*
FSA	0.31	0.59
VFL/GM	−0.42	0.70

Loadings for a principal component analysis of distal phalanx shape variables are reported as Pearson correlation coefficients between each variable and principal component. See Table 3 and Materials and Methods for measurement details.

*Non-significant correlations (p>0.05).

**Table 5:** ANOVAs and post hoc tests of distal phalanx variables.

Variable	ANOVAs	Ungulae means	Grooming means	Tamhane's T2 Tests
BH/GM*	F = 33.530; p<0.001	0.903 (0.009)	1.255 (0.096)	p = 0.022
TPL/GM	F = 23.303; p<0.001	2.554 (0.094)	3.303 (0.136)	p<0.001
SH-¼/GM	F = 49.090; p<0.001	0.689 (0.007)	0.905 (0.040)	p = 0.030
SH-¾/GM	F = 15.783; p<0.001	0.549 (0.009)	0.444 (0.005)	p<0.008
BW/GM	F = 8.568; p<0.001	1.266 (0.047)	1.313 (0.024)	p = 0.977
SW-¼/GM	F = 8.369; p<0.001	0.693 (0.011)	0.673 (0.021)	p = 0.999
SW-¾/GM	F = 15.249; p<0.001	0.691 (0.024)	0.624 (0.016)	p = 0.723
FSA	F = 19.234; p<0.001	77.040 (42.776)	61.932 (20.102)	p<0.001
VFL/GM	F = 40.891; p<0.001	2.078 (0.115)	1.230 (0.022)	p<0.001
VFL/TPL	F = 88.122; p<0.001	0.817 (0.013)	0.376 (0.004)	p<0.001
(SH-¼)/SH(-¾)	F = 15.729; p<0.001	1.281 (0.047)	2.079 (0.264)	p<0.002
(SW-¾)/TPL	F = 14.184; p<0.001	0.279 (0.008)	0.193 (0.003)	p<0.006

ANOVAs among distal phalanx groups (ungular, tegular, falcular, and grooming phalanges) for indices of distal phalanx shape and an angular measurement (FSA). The columns labeled ungulae and grooming means display the means and variances (in parentheses) for the ungulae and grooming claw groups respectively. Post hoc tests compare ungular and grooming phalanx groups. Comparisons are considered significant at the Bonferroni adjusted alpha of 0.0042. See Table 3 and Materials and Methods for measurement and group details.

*GM: “geometric mean” of all linear measurements which is used for size standardization.

**Figure 12:**
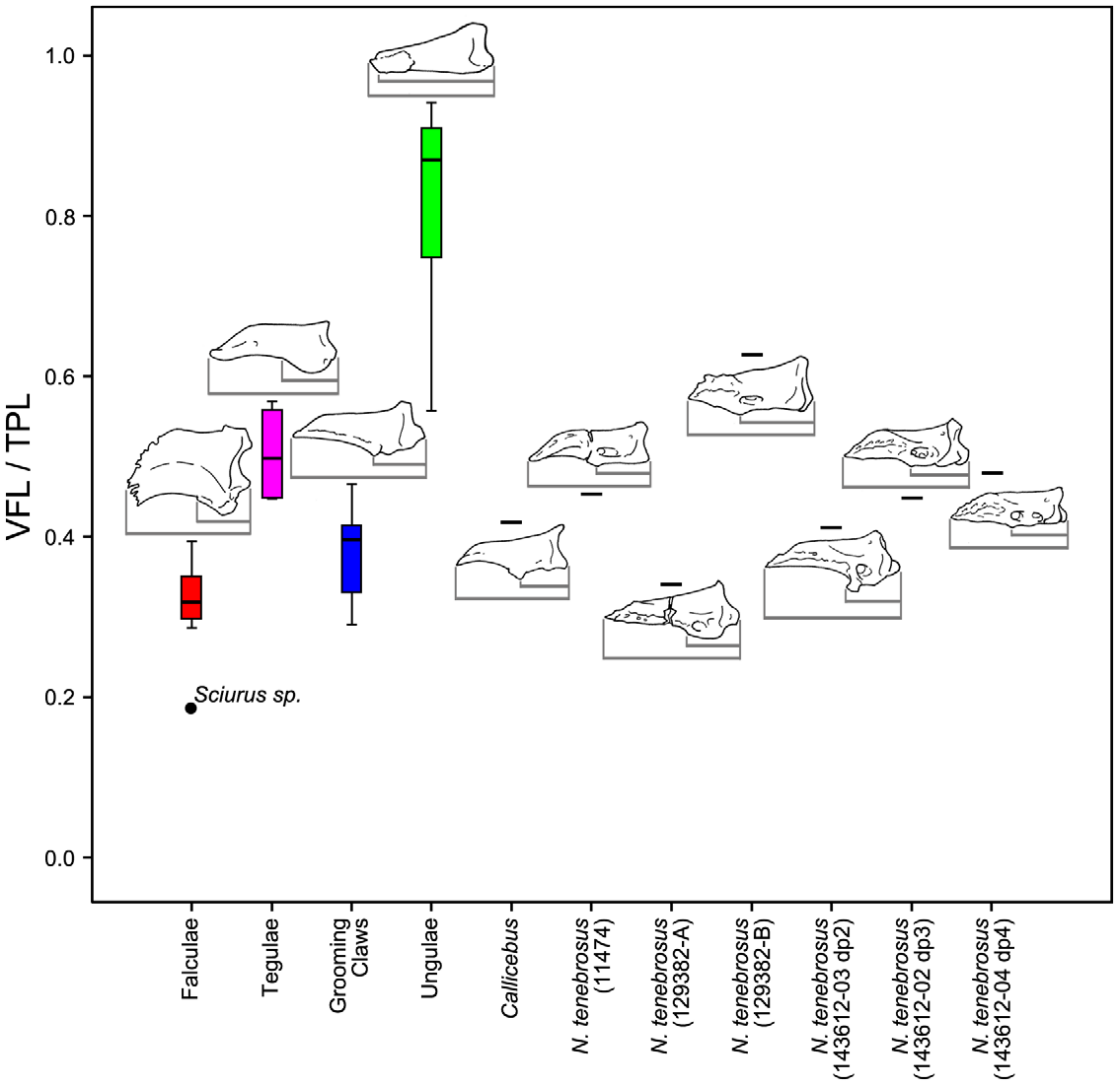
Boxplot of volar feature length scaled to total distal phalanx length. Raw values of volar feature length (VFL) are divided by total phalanx length (TPL). VFL and TPL are demonstrated using illustrations of specimens scaled to the same length and oriented according to their long axes. The median specimen from each extant group is illustrated: *Galeopterus vareigatus* represents the falculae group; *Leontopithecus sp.*, tegulae; *Lemur catta*, grooming claws; and *Galago senegalensis*, ungulae. See Materials and Methods for measurement descriptions.

**Figure 13:**
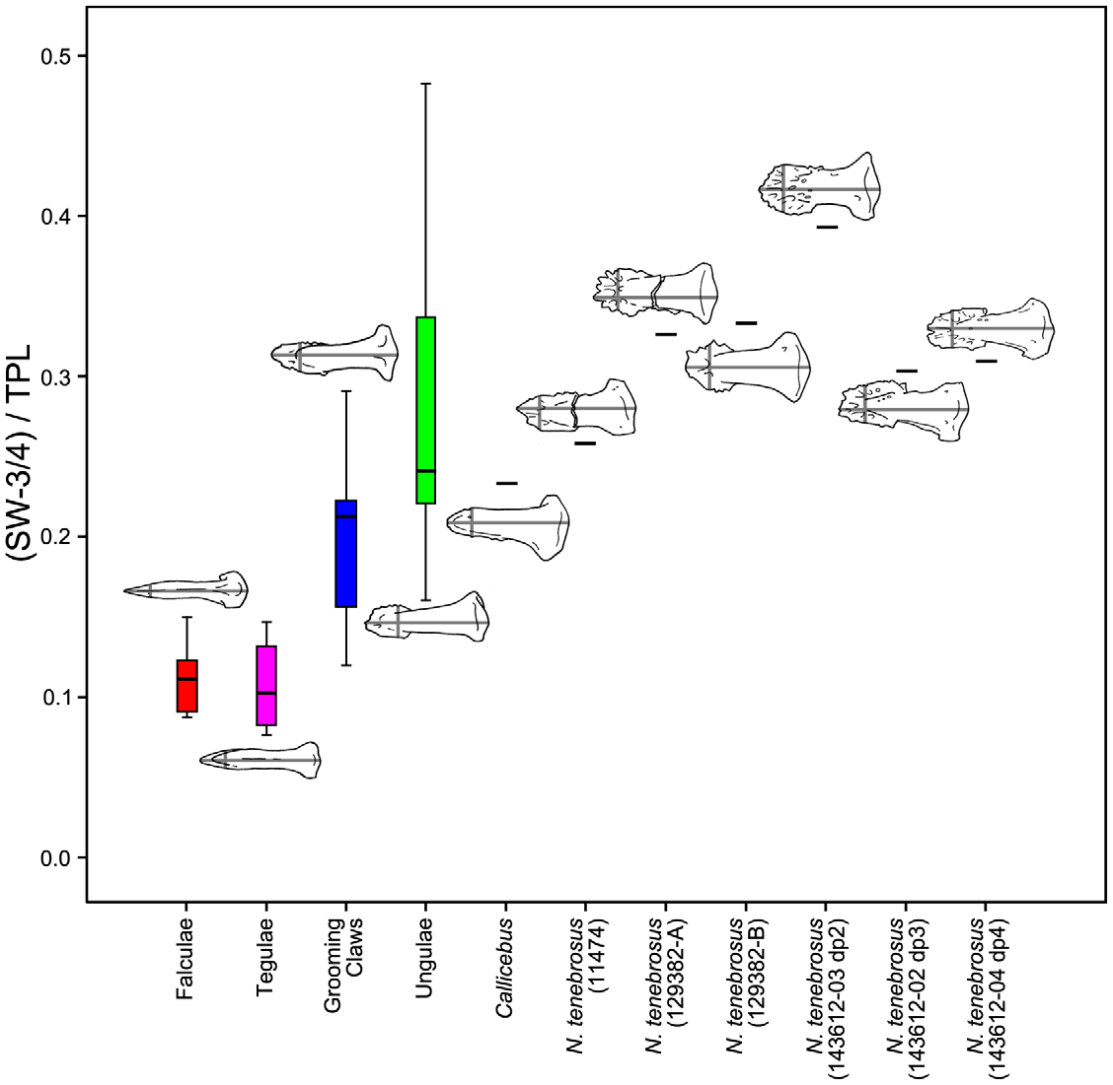
Boxplot of distal phalanx distal shaft width scaled to total phalanx length. Raw values of shaft width taken at ¾ of the length of the shaft (SW-3/4) are divided by total phalanx length (TPL). SW-3/4 and TPL are demonstrated using illustrations of specimens scaled to the same length. The median specimen from each extant group is illustrated: *Tupaia glis* represents the falculae group; *Callithrix sp.*, tegulae; *Galago senegalensis*, grooming claws; and *Saimiri sp.*, ungulae. See Materials and Methods for measurement descriptions.

**Figure 14:**
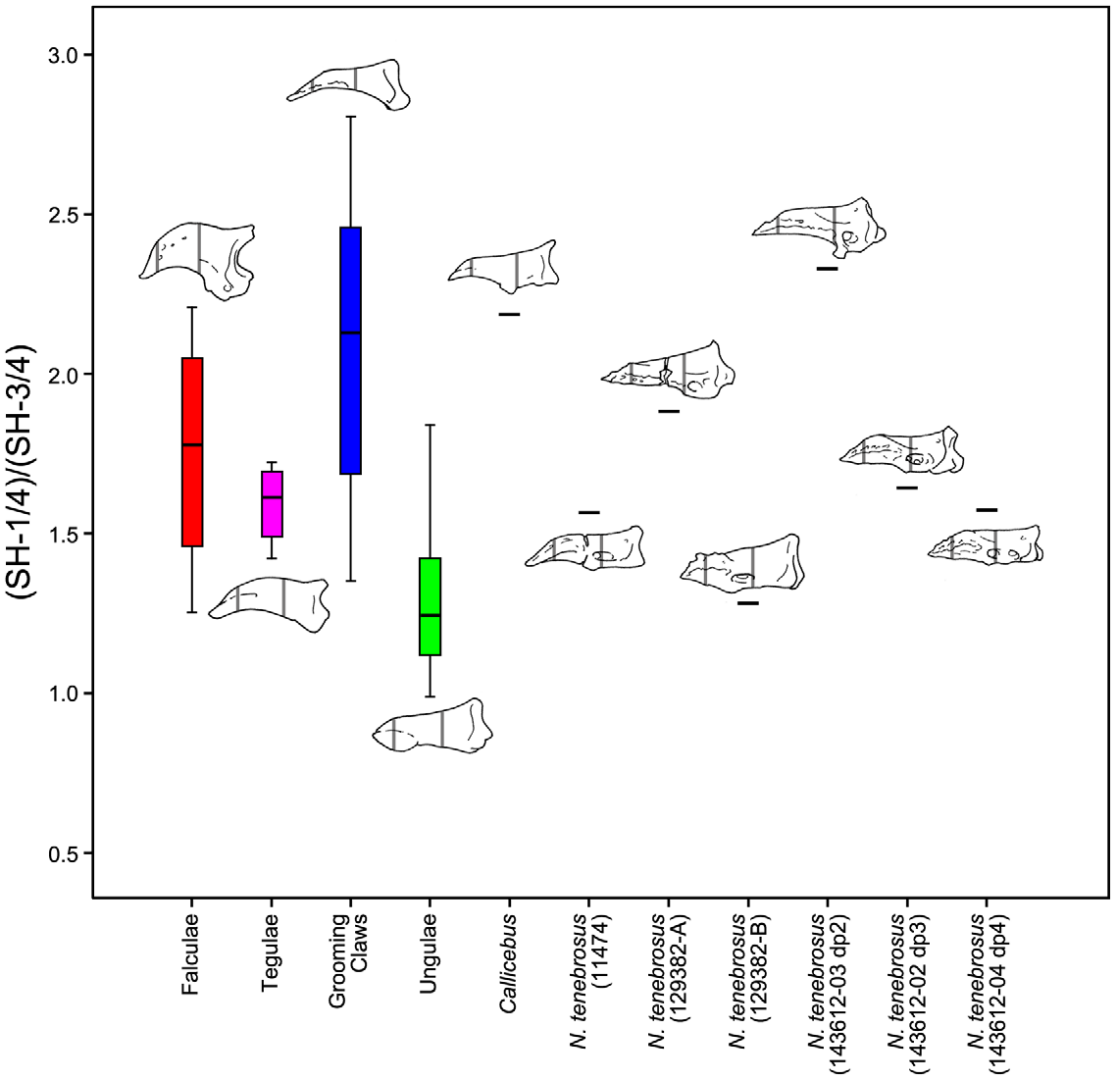
Boxplot of ratio of distal phalanx proximal shaft height to distal shaft height. Proximal height (SH¼) is measured at a point along the shaft of the phalanx that is ¼ of the distance from the proximal end to the tip, distal height (SH¾) at ¾ the distance. The median specimen from each extant group is illustrated: falculae - *Phalanger orientalis*; tegulae - *Callithrix sp.*; grooming phalanges - *Nycticebus coucang*; ungulae - *Chlorocebus aethiops.* See Materials and Methods for measurement descriptions.

We also looked at variation in several, more-simply constructed variables. Specifically, we analyzed the ratio of VFL to TPL as an alternative expression of relative volar feature length (Fig. 12), of SW-3/4 to TPL as an alternative expression of relative mediolateral width of the apical tuft (Fig. 13), and of SH-1/4 to SH-3/4 as a previously unquantified measure of how substantially the shaft tapers (Fig. 14). ANOVA using unguis groups with these variables, like the analyses of the geometric mean-standardized variables, yielded highly significant results (Table 5). Tamhane's T2 tests (again, assessed at the Bonferroni adjusted alpha of 0.0042) suggested significant differences between ungular and grooming phalanges for VFL/TPL and (SH-1/4)/(SH-3/4) (p<0.0042). (SW-3/4)/TPL had a low p-value (p<0.006), but was higher than the Bonferroni adjusted critical value for alpha. (SW-3/4)/TPL measures the width of the distal portion of the shaft which is also the apical tuft in primate distal phalanges. This presents a different view than the previous analyses in the case of SW-3/4, as the results show that there is a strong trend in which grooming phalanges have more narrowed apical tufts than ungular phalanges when compared to phalangeal length. The lower values of SH-1/4 to SH-3/4 for grooming phalanges yields information not available in the geometric mean-standardized variables indicating a more strongly tapering shaft is also diagnostic of grooming phalanges.

Focusing on variables for which groups of extant ungular and grooming phalanges significantly differ (TPL, FSA, VFL, VFL/TPL, [SH-1/4]/[SH-3/4]; p<0.0042; Table 5) as well as SH-3/4, *t*-values from *t*-tests comparing AMNH 129382-A and AMNH 143612-03 (dp2) to extant samples show these fossils to be more similar to extant grooming phalanges than to ungular phalanges (though not significantly different from either group; p>0.0042). The only exception is that AMNH 143612-03 is more similar to ungular phalanges in having a somewhat shorter shaft relative to its geometric mean (TPL), but again, it is not significantly different from either group (Table 6). Furthermore, AMNH 129382-A and AMNH 143612-03 (dp2) are the *only* fossil specimens that are more similar to grooming phalanges in their values for FSA and SH-3/4, reflecting their pronounced dorsal inclination and shallow distal shafts (as before, they are not significantly different from either group; p>0.0042). While most fossils have high values of (SH-1/4)/(SH-3/4) exhibiting an affinity to extant grooming phalanx morphology in this way, AMNH 129382-A and AMNH 143612-03 (dp2) have the highest values, demonstrating that they exhibit more pronounced distal tapering of their shafts than the other fossil specimens. In fact, the value of (SH-1/4)/(SH-3/4) for AMNH 143612-03 (dp2) is the only value for any variable of any fossil that is significantly different from the ungular phalanx group.

**Table 6:** *T*-tests comparing *Notharctus* distal phalanx morphology to extant primate ungular and grooming phalanges.

Variable		11474^a^	129382-A	129382-B	143612-03 (dp2)	143612-02 (dp3)	143612-04 (dp4)
BH/GM	U	−0.27	0.69	−0.33	0.62	−0.17	−0.26
	G	−1.17	−0.88	−1.19	−0.91	−1.14	−1.16
TPL/GM	U	1.18	1.46	−0.97	0.14	0.54	0.34
	G	−0.99	−0.77	−2.73	−1.84	−1.51	−1.67
SH-¼/GM	U	1.45	1.42	0.53	*3.61	1.61	1.31
	G	−0.45	−0.47	−0.82	0.41	−0.39	−0.51
SH-¾/GM	U	−0.32	−1.21	0.22	−1.25	−0.49	−0.42
	G	0.98	−0.19	1.69	−0.24	0.76	0.84
BW/GM	U	0.43	0.24	0.56	0.02	0.42	0.76
	G	0.29	0.04	0.48	−0.26	0.28	0.74
SW-¼/GM	U	−0.08	0.49	1.23	−0.06	0.38	0.08
	G	0.08	0.48	1.01	0.09	0.41	0.19
SW-¾/GM	U	0.40	1.84	0.38	2.09	0.86	0.84
	G	0.97	2.66	0.94	2.95	1.50	1.48
FSA	U	0.95	−2.40	0.53	−3.02	−0.02	0.14
	G	*4.58	−0.19	*3.99	−1.08	3.19	3.42
VFL/GM	U	−2.18	−3.04	−1.93	−2.92	−2.49	−2.32
	G	0.59	−1.31	1.16	−1.04	−0.08	0.29
VFL/TPL	U	*−3.17	*−4.14	−1.65	*−3.52	*−3.21	−2.94
	G	1.22	−0.57	*4.01	0.57	1.14	1.65
(SH-¼)/SH(-¾)	U	1.28	2.72	0.01	*4.74	1.64	1.32
	G	−0.96	−0.37	−1.48	0.47	−0.81	−0.94
(SW-¾)/TPL	U	−0.23	0.50	0.58	1.22	0.26	0.32
	G	1.21	2.46	2.59	3.69	2.04	2.15

The results of *t*-tests comparing *Notharctus tenebrosus* specimens to ungulae and grooming claw group means. For each variable, the first row (‘U’) is comprised of *t*-values from comparisons to ungular phalanges; the second (‘G’) from comparisons to grooming phalanges. The smallest absolute *t*-value indicates which extant group the fossil is most similar to. Asterisks mark significant comparisons at the Bonferroni corrected alpha of 0.0042.

a All specimen numbers are from AMNH.

*Significant comparisons (p<0.0042).

Turning to the remaining fossil specimens, AMNH 129382-B, AMNH 11474, AMNH 143612-02 (dp3), AMNH 143612-04 (dp4) are similar to ungular phalanges as they have less dorsally projecting shafts than grooming phalanges (their FSA is higher; this difference is significant in the case of AMNH 11474 and AMNH 129382-B; p<0.0042) and deeper distal shafts (SH-3/4; though not significantly so). However, it should be noted that some specimens (AMNH 11474, AMNH 143612-02 [dp3] and -04 [dp4]) are similar to grooming phalanges, AMNH 143612-03 (dp2), and AMNH 129382-A in also possessing shorter volar features (VFL/TPL) than those of extant ungular phalanges (Fig. 12; p<0.0042). On this basis alone, these *Notharctus* distal phalanges could be classified as grooming phalanges. However, when taking into account other variables in this analysis (Figs. 10, 11, 14), such a designation is clearly incorrect. Therefore, a short VFL/TPL alone should not be considered diagnostic evidence of a grooming phalanx. Rather, it is a combination of traits that distinguishes grooming from ungular phalanges: a short volar process combined with a dorsally canted and strongly distally tapering (in height) shaft. Finally, it is interesting to note that, in the case of relative width of the apical tuft, (SW-3/4)/TPL, all fossil specimens (including AMNH 129382-A and AMNH 143612-03 [dp2]) are more similar to ungular phalanges than to grooming phalanges.

#### Comparisons of foot proportions among extant and fossil euprimates

We compared the preserved pedal elements in AMNH 143612 and AMNH 143640 to a sample representing those of extant primates in order to determine whether there are distinctive functional and or/phylogenetic patterns distinguishing groups of extant primates and linking the fossils to one or another of these groups. Digits supporting grooming claws appear to be relatively shorter than those that do not, so we predicted that extant primates with a grooming claw will also have a shorter second digit. Furthermore, it has been observed that prosimian primates have a shorter third digit than fourth digit; we evaluate whether *Notharctus* also exhibits this feature. Our measurements are taken in the same way as those previously published for *Darwinius masillae* [6], which we include here as well. Two discriminant function analyses (DFA) were conducted using a training set of 279 individuals representing 39 species of extant primates (Table S2a, S2b). The first discriminant function analysis of thirteen geometric mean-standardized variables representing the lengths of mt1-5, pp1-5, and ip2-3;5 (Table S3) had a 97.1% cross-validated success rate in assigning taxa to the appropriate one of nine designated groups (Table S2; Table S4a). *Notharctus* was assigned to Cheirogaleiidae with a probability 0.92 (group #4, Table S2). *Darwinius* was assigned to a group consisting of several species of galago with a probability of 1.00 (group #1, Table S2).

A second DFA was run using only phalangeal measurements (no metatarsals). In this case there were seven geometric mean-standardized measurements analyzed (Table S4b). Only 78.9% of taxa were correctly identified (which is still surprisingly accurate for nine groups). *Notharctus* was classified as an anthropoid with a probability of 0.76 (group #9, Table S2), while *Darwinius* was classified as a member of a group including several lemurid species with a probability of 0.52 (group #7, Table S2). However, in this analysis various strepsirrhines were miss-classified as anthropoids as well. These included 1 specimen out of 51 lemurids with a probability of 0.45 (group #7), and 2 out of 33 indriids with probabilities of 0.77 and 0.29 (group #6).

**Figure 15:**
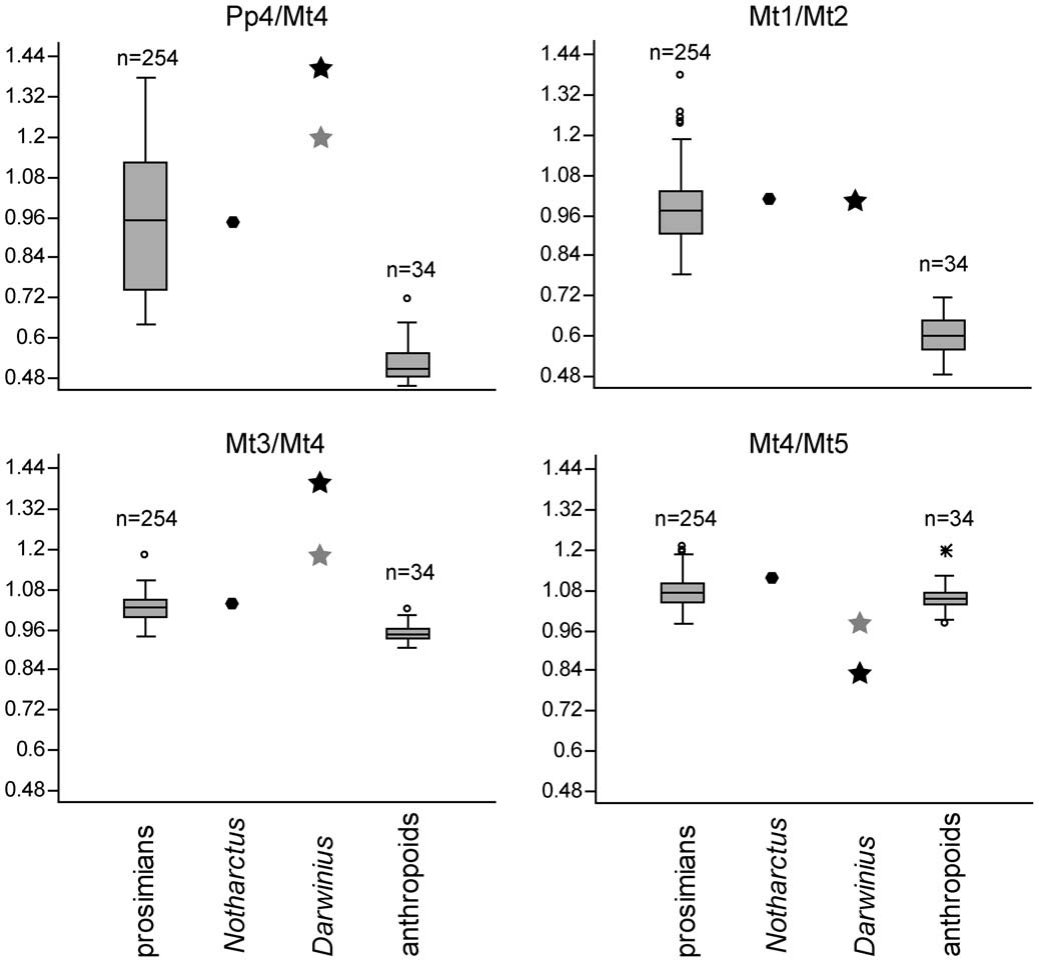
Boxplots of pedal proportions. Abbreviations: Pp, proximal phalanx; Mt, metatarsal. Statistical treatment of their data is given in Table 7. See Materials and Methods for further information.

Next, we examined univariate patterns reflected by several different inter-element ratios (Fig. 15). We looked at two sets of ratios: those involving metatarsals, and those involving only proximal phalanges. For four ratios involving metatarsals, anthropoid and prosimian groups were significantly different from each other (Table 7). Compared to anthropoids, prosimians exhibit diagnostically longer fourth proximal phalanges relative to fourth metatarsals; longer hallucal metatarsals relative to second metatarsals; and longer third metatarsals relative to fourth metatarsals (p<0.0001 using Student's *t*-test and the non-parametric Mann-Whitney U test for all comparisons). In these three ratios, both *Darwinius* and *Notharctus* are outside the range of anthropoid values and within or (in one case) beyond the range of prosimian values (Fig. 15). Using one-sample *t*-tests, we compared the values of *Notharctus* and *Darwinius* to each of the extant distributions for all variables (Table 7). Comparisons are considered significant at the Bonferroni adjusted alpha of 0.0125. *Notharctus* is not significantly different from the prosimian group for any variable, but is significantly different from the anthropoid group for pp4/mt4, mt1/mt2, and mt3/mt4 (p<0.0125). The ratio involving metatarsals mt4/mt5 was not strongly different between prosimians and anthropoids. In fact, the only outlier to the distribution is *Darwinius*. This seems to be the result of an inaccurate measurement for mt4. We use a correction based on the extant sample as well as the mt3 and mt5 of *Darwinius* to estimate a more plausible length for this bone (see Appendix S1, section 2 for details). The gray symbol in figure 15 represents ratios using this estimated value. *Darwinius* was significantly different from the prosimian group for mt3/mt4 when using both the reported and estimated values of mt4. It was only different from these groups for mt4/mt5 when using the value reported by Franzen et al. [6] (p<0.0125). *Darwinius* was not significantly different from prosimians for any other ratio, but was significantly different from anthropoids (p<0.0125) for all ratios with the exception of mt4/mt5 when using the estimated value of mt4 (p = 0.035). Further, the absolute value of the *t*-statistic can be used to determine to which extant group each fossil is most similar. This is particularly useful when a specimen is significantly different from both groups, or not significantly different for either group (see results for analyses of distal phalanges above). Both *Notharctus* and *Darwinius* are most similar to the prosimian group for all variables in their *t*-statistic.

**Table 7:** *T*-tests comparing foot proportions.

Two-sample *t*-tests (prosimians v. anthropoids)						
	Prosimians		Anthropoids			
Variable	mean	var	mean	var	*t* *	p
pp4/mt4^a^	0.947	0.040	0.516	0.003	27.481	<0.0001
mt1/mt2	0.977	0.010	0.600	0.003	31.942	<0.0001
mt3/mt4	1.021	0.001	0.948	0.001	14.831	<0.0001
mt4/mt5	1.074	0.002	1.056	0.001	2.447	0.0343

Notharctus *t*-tests						
		Prosimians		Anthropoids		
Variable	Value	*t*	p	*t*	p	
pp4/mt4	0.943	−0.021	0.983	7.674	<0.0001	
mt1/mt2	0.999	0.220	0.826	6.747	<0.0001	
mt3/mt4	1.036	0.432	0.673	3.387	0.002	
mt4/mt5	1.11	0.840	0.402	1.482	0.148	

Darwinius *t*-tests						
		Prosimians		Anthropoids		
Variable	value	*t*	p	*t*	p	
pp4/mt4	1.407/1.189^b^	2.294/1.206	0.023/0.229	16.017/12.097	<0.0001/<0.0001	
mt1/mt2	0.994	0.170	0.865	6.662	<0.0001	
mt3/mt4	1.390/1.175	10.416/4.347	<0.0001/<0.0001	17.040/8.748	<0.0001/<0.0001	
mt4/mt5	0.820/0.970	−5.319/−2.172	<0.0001/0.031	−6.054/−2.208	<0.0001/0.035	

Results of two-sample *t*-tests comparing extant primate pedal proportions and *t*-tests comparing those of *Notharctus tenebrosus* (AMNH 143612/143640) and *Darwinius* to extant primate groups. For the fossil analyses, the smallest absolute *t*-value indicates which extant group the fossil is most similar to. Comparisons are considered significant at the Bonferroni adjusted alpha of 0.0125.

*t-statistic has been adjusted for comparison of distributions with unequal variance.

a Abbreviations: Mt, metatarsal; proximal phalanx; ip, intermediate phalanx.

b For comparisons of *Darwinius* ratios involving mt4, both the reported and estimated values were analyzed (See Materials and Methods and Appendix S1). Therefore, the results of comparisons of the reported values are placed before the ‘/’ while those of the estimated values follow.

**Figure 16:**
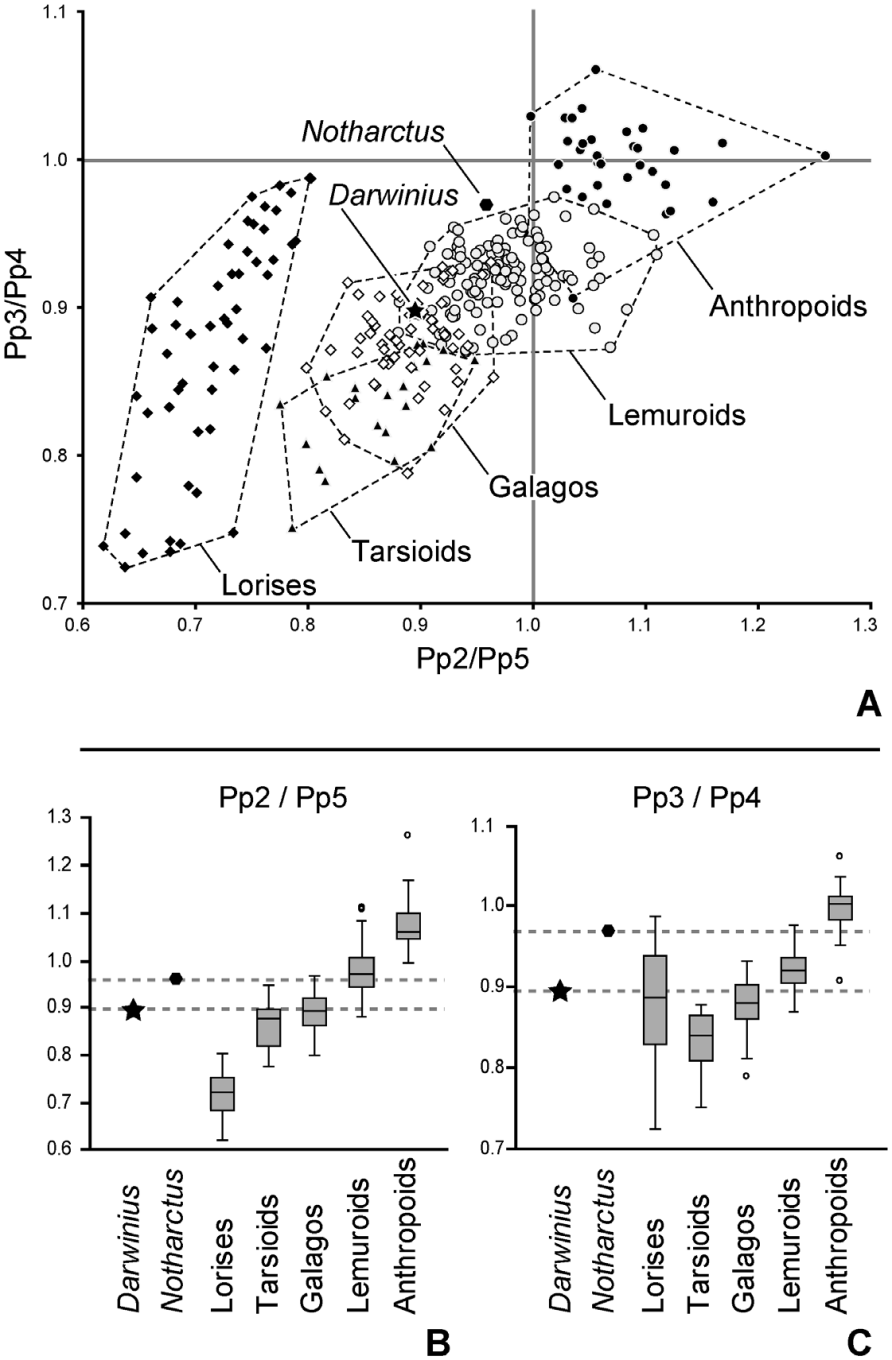
Plots of pp2 length/pp5 length and pp3 length/pp4. A, Bivariate plot of pp2/pp5 length and pp3/pp4 length: closed circles (anthropoids); closed triangles (tarsiiforms); closed diamonds (lorises); open diamonds (galagos); open circles (lemuroids); closed octagon (*Notharctus*); closed star (*Darwinius*). Abbreviations: Pp, proximal phalanx; Mt, metatarsal. B, Univariate boxplots of pp2/pp5 length. C, univariate boxplots of pp3/pp4 length. Dashed lines in A and B show position of fossils relative to extant groups.

Next, we examined two ratios relating to proximal phalanges only. These include the ratio of pp2/pp5 and the ratio of pp3/pp4. We chose these comparisons because we predicted them to vary with grasp-type (I–II and I–V grasps), grooming claw presence (dp2–3, just dp2, or none), and phylogenetic affinity (dramatically shortened digit 2 of lorises). In this case, our predictions about significant variance components lead to parsing the sample into five groups (tarsioids, lorises, galagos, lemuroids, and anthropoids). We ran ANOVAs and non-parametric Kruskal-Wallis tests on these two ratios; both were highly significant (p<0.0001; Table 8). We also ran multiple post-hoc comparisons using Tukey's comparisons and Mann-Whitney tests. Both sets of tests yielded the same results, showing significant differences between groups for all pairwise comparisons (p<0.0001) with two exceptions: tarsioids and galagos were not significantly different for pp2/pp5 and galagos and lorises were not significantly different for pp3/pp4 (comparisons were considered significant at the Bonferroni adjusted alpha of 0.025). Plotting both ratios together (Fig. 16A) reveals interesting differences between different groups. *Notharctus* is close to the region of overlap between anthropoids and lemurids, whereas *Darwinius* is in a region of overlap of galagos with tarsiers and lemuroids. For the pp2/pp5 ratio, both *Notharctus* and *Darwinius* are outside the range of sampled anthropoids, reflecting shorter digit 2 phalanges than digit 5 phalanges in the fossils (a similarity to prosimians). For pp3/pp4, *Notharctus* is at the low end of the anthropoid range while *Darwinius* is well below it, reflecting shorter digit 3 phalanges compared to digit 4 phalanges for the fossils (again, a similarity to prosimians). When comparing the magnitudes of the *t*-statistics from two-sample *t*-tests, *Notharctus* is most similar to the lemuroid group for pp2/pp5 and to the anthropoid group for pp3/pp4, whereas *Darwinius* is most similar to the galago group for pp2/pp5 and to the loris group for pp3/pp4 (Table 8).

**Table 8:** Analyses of proximal phalanx indices.

Comparisons among extant groups												
			Lorises		Tarsioids		Galagos		Lemuroids		Anthropoids	
Variable	F*	H*	mean	var	mean	var	mean	var	mean	var	mean	var
pp2/pp5	429.500	239.100	0.717	0.002	0.863	0.002	0.888	0.001	0.975	0.002	1.074	0.003
pp3/pp4	76.1200	151.900	0.874	0.006	0.835	0.001	0.878	0.001	0.920	0.001	0.988	0.001

Fossil *t*-tests												
			Lorises		Tarsioids		Galagos		Lemuroids		Anthropoids	
Fossil	Variable	Value	t	p	t	p	t	p	t	p	t	p
*Notharctus*	pp2/pp5	0.960	5.157	0.000	2.031	0.055	1.914	0.061	−0.335	0.738	−2.130	0.041
	pp3/pp4	0.968	1.186	0.241	3.799	0.001	2.999	0.004	2.094	0.038	−1.033	0.309
*Darwinius*	pp2/pp5	0.894	3.757	0.000	0.647	0.525	0.170	0.866	−1.799	0.075	−3.359	0.002
	pp3/pp4	0.896	0.279	0.782	1.750	0.095	0.595	0.544	−1.063	0.290	−3.552	0.001

Results of ANOVAs (F-statistic) and Kruskal-Wallis tests (H-statistic) among extant proximal phalanx indices and *t*-tests comparing those of *Notharctus tenebrosus* (AMNH 143612/143640) and *Darwinius* to extant primate groups. See results for post hoc comparisons between extant groups. For the fossil analyses, the smallest absolute *t*-value indicates which extant group the fossil is most similar to. Comparisons are considered significant at the Bonferroni adjusted alpha of 0.025.

*F and H statistics are highly significant for all comparisons (p<0.0001).

### Cladistic analysis

We analyzed the significance of new and previously known morphology of *Notharctus* using the framework provided by Gingerich et al. [8]. Inclusion of this fossil taxon is appropriate for this framework because it 1) has fewer missing data, relative to Gingerich et al.'s matrix, than *Darwinius* itself (Table S6), and 2) is an accepted close-relative to *Darwinius*, but is likely more primitive given its older age >48.5 Ma [46] and more direct relationship to *Cantius* (*Pelycodus*), the common ancestor of European and North American adapiforms e.g., [44], [47]. While we attempted to adhere to Gingerich et al.'s “few-taxa, few-characters” philosophy of cladistic analysis, we were also compelled to add the taxon *Catopithecus browni*, (a definitive representative of the late Eocene Fayum anthropoids) due to the acknowledged importance of such fossil taxa for understanding anthropoid evolution [47].

Like *Notharctus*, *Catopithecus* is appropriate for Gingerich et al.'s matrix because, it too, is more complete relative to this matrix than *Darwinius*. These two species are included as it has been shown, both empirically and through simulation studies, that the addition of fossil taxa helps in the assessment of character state polarity and generally helps to increase accuracy during phylogenetic analysis e.g., [48], [49], [50], [51], [52], [53], [54]. Our analyses of this matrix are organized into three sections. Several iterations of analysis are necessary due to the need to (i) reproduce Gingerich et al.'s [8] results using their exact matrix with no taxon additions or character changes/additions; (ii) correct several inaccurate codings in the Gingerich et al. [8] matrix (Table 9); and (iii) add characters that were omitted from Gingerich et al.'s [8] original matrix, which have previously demonstrated significance for separating major phylogenetic groupings of primates (Table 10).

**Table 9:** Code and coding scheme changes to matrix of Gingerich et al. [8].

**Character 6: Olfactory Bulb Size**
Re-coded from a two-state character to a three state, ordered character: relatively small = 0; intermediate = 1; relatively large = 2, e.g., [69], [70]
**Character 9: Mandibular Corpus Depth**
*Darwinius* changed from “1” (deep) to “0” (shallow) (see Appendix S1)
**Character 11: Postorbital Closure**
Re-coded from a two-state character to a three state, ordered character: postorbital bar = 0; partial closure = 1; full closure = 2 [1], [6], [8]
*Tarsius* coding changed to “partial closure”
**Character 13: Mandibular Symphysis Fusion**
Re-coded from a two-state character to a three state, ordered character: open = 0; partially fused = 1; fully fused = 2 [58], [71]
*Darwinius* coding changed to “mandibular symphysis partially fused” [6]
“Lemuroidea” changed to “0/1” [58], [71]
“Tarsioidea” changed to “0/1” [58], [71]
**Character 19: Lower molar ** [**7**] ** ordered**
Re-coded from a two-state character to a three state, ordered character: paraconids present = 0; paraconids reduced = 1; paraconids absent = 2
*Darwinius* coding changed to “paraconids reduced”
Ceboidea coding changed to “1/2”
**Character 21: Fibular Facet of Astragalus Orientation**
*Darwinius* changed from “0” (vertical astragalar fibular facet) to “?,” e.g., [2], [59]
**Character 22: Pes condition**
*Tupaia* changed from “0” (tarsifulcrumating foot) to “1” (metatarsifulcrumating foot), e.g., [55], [57]
**Character 23: Mesocuneiform expansion**
*Tupaia* changed from “0” to “1” (expanded), e.g., [55], [56]
“Lorisoidea” changed from “0” to “1” [55]
**Character 24: Length of Pedal Digits**
*Tupaia* changed from “0” (third digit longest) to “1” (fourth digit longest), see Table 3 of [57]
*Darwinius* changed from “0” (third digit longest) to “1” (fourth digit longest), see appendices of [6]
“Ceboidea” changed from “0” (third digit longest) to “0/1,” see Table 3 of [57]
**Character 25: Pedal Digit II Form**
Re-coded from a two-state character to a three state, ordered character: Falculae = 0; Grooming claw = 1; Ungulae = 2, current study and [38]
*Darwinius* changed to “?” (results of current study)
*Tupaia* re-coded with “Falculae”
“Ceboidea” is coded as polymorphic “1/2,” current study and [38]

Summary of changes to the matrix of Gingerich et al. [8]. See Appendix S1 for further details.

**Table 10:** Characters added to corrected matrix of Gingerich et al. [8].

**Character 31: Flexor fibularis groove position, e.g., ** [**72**]
**0** in-line with medial tibial facet (tupaioids; tarsioids; all anthropoids)
**1** lateral to medial tibial facet (strepsirrhines; *Notharctus*)
**Character 32: Posterior aspect of astragalus trochlea, e.g., ** [**57**]
**0** unexpanded (tupaioids; some lorisoids; tarsioids; all anthropoids)
**1** expanded into shelf (lemuroids; some lorisoids; *Notharctus*)
**Character 33: Peroneal tuberosity on mt1 ** [**57**]
**0** reduced (tupaioids; all anthropoids)
**1** enlarged (strepsirrhines; *Notharctus*; tarsioids)
**Character 34: Medial tibial facet ** [**57**]
**0** shallow (tupaioids; all anthropoids)
**1** deep (strepsirrhines; *Notharctus*; tarsioids)
**Character 35: Hypoconulid lobe of M_3_, e.g., ** [**73**]
**0** abbreviated (tupaioids; *Catopithecus*; ceboids; some cercopithecoids; hominoids)
**1** developed (strepsirrhines; *Notharctus*; *Darwinius*; tarsioids; some cercopithecoids)
**Character 36: Cuboid facet of navicular contact ** [**10**]
**0** only contacts ectocuneiform facet (tupaioids; all anthropoids)
**1** contacts both ecto- and mesocuneiform facet (strepsirrhines; *Notharctus*)
**Character 37: Divergence of big toe ** [**74]**, [75****] ** ordered**
**0** not divergent (tupaioids)
**1** moderate divergence (all anthropoids; *Catopithecus*)
**2** extreme divergence (strepsirrhines; *Darwinius*; *Notharctus*)
**Character 38: Orbit diameter/Activity pattern ** [**76**] ** ordered**
**0** large/nocturnal (some tupaioids; some lemuroids; lorisoids; tarsioids; *Darwinius*)
**1** moderate/cathemeral (some lemuroids)
**2** small/diurnal (some tupaioids; some lemuroids; *Notharctus*; *Catopithecus*)
**Character 39: Tibia medial malleolus rotation, e.g., ** [**10]**, [12****] ** ordered**
**0** unrotated (some tupaioids)
**1** slightly rotated (some tupaioids, anthropoids)
**2** rotated (lorisoids; lemuroids; *Notharctus*)

Summary of characters added to the corrected matrix of Gingerich et al. [8]. See Appendix S1 for further details.

Reanalysis of Gingerich et al.'s [8] original matrix using exhaustive searches in PAUP 4.0b10 reproduced their result by achieving the same two most parsimonious trees. In these cladograms have he following statistics: Tree Lengths (TL) = 37 steps, Consistency index (CI) = 0.8378, Homoplasy index (HI) = 0.1622, Retention index (RI) = 0.9016, and Rescaled consistency index (RC) = 0.7554 (See Gingerich et al. [8] for original character descriptions and character state designations).

**Figure 17:**
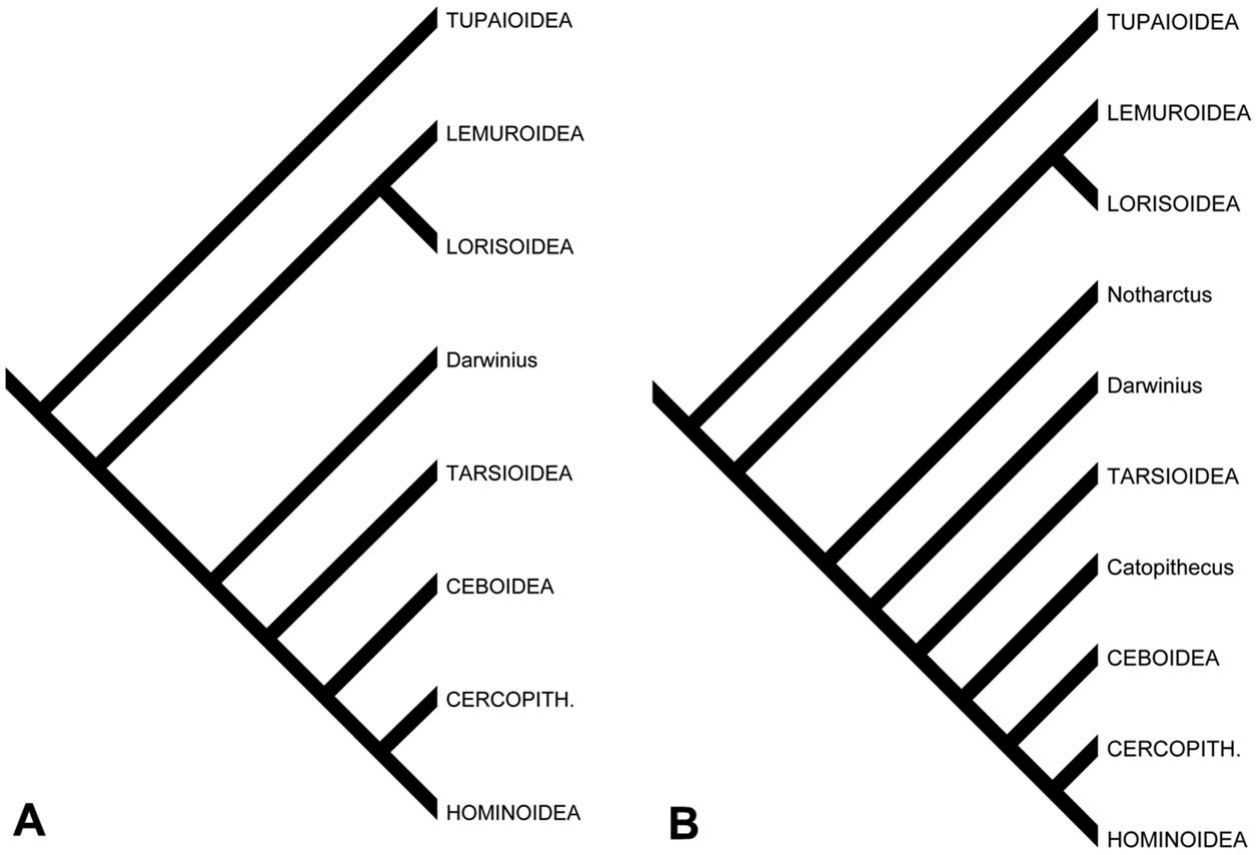
Trees resulting from analysis of corrected matrix of Gingerich et al. (2011). A, Single most parsimonious tree including extant taxa and *Darwinius* only. This tree topology was produced from an exhaustive search using the corrected version of the Gingerich et al. [8] matrix (corrections noted in Table 9;see also Appendix S1, section 10 for nexus file). This topology was also produced by analysis of the corrected matrix with additional characters noted in Table 10 (see Appendix S1, section 17 for nexus file). These trees differ only in statistics such as length, CI, HI, RI, and RC. See Results. B, Single most parsimonious tree resulting from analysis of the corrected matrix with additional fossil taxa added (see SI Appendix S1, sections 14–16 for nexus files).

We next ran a series of analyses, sequentially adding codings first for *Notharctus*, then *Catopithecus*, in order to establish where these taxa would have been placed if Gingerich et al. [8] had included them (see Appendix S1, section 1; Figure S1, S2). While we feel the results of these analyses are informative in certain ways, they are tangential to the focal analyses of this study which were based on a modified version of Gingerich et al.'s [8] original matrix. The need to modify character codings in the original character matrix of Gingerich et al. [8] was revealed by: 1) referencing data available in the literature e.g., [6], [9], [55], [56], [57], [58] and 2) a new quantitative analysis of mandibular depth presented here for the first time [see Table 9, Appendix S1 (section 2), Figure S3, and Table S5 for details on corrections and nexus file of corrected matrix (section 10)]. Analysis of this corrected matrix resulted in a single most parsimonious tree in which *Darwinius* is a stem-haplorhine (Fig. 17A) as opposed to a stem anthropoid as indicated by analysis of Gingerich et al.'s [8] original matrix (TL = 42, CI = 0.8571, HI = 0.1429, RI = 0.9016, RC = 0.7728). Next, we added additional fossil taxa *Notharctus* and *Catopithecus* and tested the effects of coding the presence/absence of a grooming claw in different ways, to reflect results of our comparative analyses [see Appendix S1 for a version in which only *Notharctus* is added (section 3)]. That is, based on results of our comparative analyses, one could argue for coding *Notharctus* as having a grooming claw, as justified by the overall closest resemblance of its dp2 to such bones (Fig. 10), as well as a distinctly inclined and tapering shaft and restricted volar process on the dp2 (Figs. 11, 12, 14). Other researchers may favor the unusually wide apical tuft as indicating the lack of a grooming claw for purpose of cladistic analysis. There are three important ways to test the effect of codings here. In the first, *Notharctus* is coded as having a grooming claw, while *Darwinius* is left as a “1/2”, because the anatomy necessary to decide whether *Darwinius* had an inclined and tapering shaft, and a restricted volar process has not been observed or quantified (see Appendix S1 section 14). The next also codes *Notharctus* as having a grooming claw, while *Darwinius* is coded as lacking one, based on Gingerich et al's [8] interpretation (see Appendix S1 section 15). Finally, an iteration in which both taxa are coded as lacking a grooming claw is run (see Appendix S1 section 16). All three iterations produce the same topology, a single most parsimonious tree (Fig. 17B), indicating that the form of the second distal phalanx was no longer directly influencing the outcome. In this single most parsimonious tree, *Catopithecus* is a stem-anthropoid, *Notharctus* and *Darwinius* are stem-haplorhines, and lemurs and lorises are monophyletic strepsirrhines (TL = 45–46, CI = 0.7826–0.8000, HI = 0.2174-0.2000, RI = 0.8667–0.8784, RC = 0.6783–0.7027).

Finally, we added a number of characters to the corrected version of Gingerich et al.'s [8] matrix (Table 10). Gingerich et al. [8] indicated that they chose the characters for their analysis to specifically limit non-independence and to reflect only those well-studied traits known to distinguish modern strepsirrhines and anthropoids. Accepting this mode of construction, for our final analyses we were still compelled to add nine additional characters previously recognized as being likely synapomorphies for strepsirrhines or anthropoids, but were not included in Gingerich et al.'s [8] original matrix [see Table 10 and Appendix S1 for details on additional characters (section 4) and nexus file of revised matrix (section 17)]. For example, Williams et al. [7] listed the contacts of the cuboid facet on the navicular as a strepsirrhine trait, as convincingly demonstrated by others [10]. Franzen et al. [6] described *Darwinius* as possessing the strepsirrhine state of this feature, but then did not include it among the characters of the matrix of Gingerich et al. [8], who concluded that *Darwinius* exhibited no strepsirrhine synapomorphies.

Exhaustive search of this matrix using only Gingerich et al.'s [8] taxon sample yielded no change in topology compared to analysis of the corrected matrix without additional characters (Fig. 17A). However, the tree statistics changed (TL = 58, CI = 0.8103, HI = 0.1897, RI = 0.8571, RC = 0.6946).

**Figure 18:**
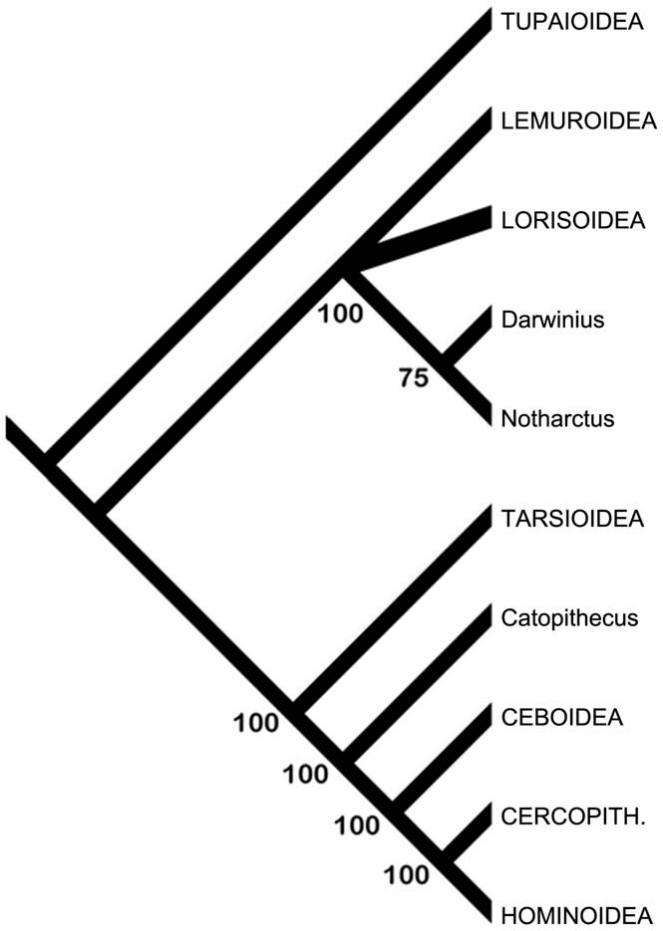
Majority rule consensus tree of extant taxa, *Darwinius*, *Notharctus*, and *Catopithecus*. Majority rule consensus tree of four equally most parsimonious trees resulting from analysis of the matrix of Gingerich et al. [8] subsequent to modifications listed in Tables 8–9, and the addition of codings for *Notharctus* and *Catopithecus* (see Appendix S1 section 4–18 for nexus files).

To the expanded character matrix we then added codings for *Notharctus* and *Catopithecus* [see Supplementary Information for revised matrix (section 5, Table S6) including codings for these taxa (section 18)]. An exhaustive search results in four most parsimonious trees (Fig 18; TL = 63, CI = 0.7460, 0.2540, RI = 0.8367, and RC = 0.6242). In all trees, *Notharctus* and *Darwinius* are members of a monophyletic strepsirrhine clade and *Catopithecus* is a stem anthropoid. Since one could argue for coding *Notharctus* as having a grooming claw, as justified by the results of this study (Figs. 10, 11, 12, 14), or lacking a grooming claw, based on the wide apical tuft, we again assigned both states to *Notharctus* and *Darwinius* in separate analyses. Alternative codings of dp2 form in *Notharctus* and *Darwinius* had no effect on the results.

Revisiting the issue of relative completeness of fossil taxa in this analysis, we note that the morphological state of *Darwinius* is unknown for 21 out of our 39 characters, whereas states are unknown for only nine characters in *Notharctus* and only 16 in *Catopithecus*. Additionally, at least three characters for which the morphology of *Darwinius* cannot yet be demonstrated include states constituting important strepsirrhine synapomorphies (character 21: slope of astragalar fibular facet; character 31, position of astragalar flexor fibularis groove; character 32, form of astragalus posterior to tibial trochlea; and character 36, contacts of navicular cuboid facet: see Appendix S1 sections 2–4). If further study reveals *Darwinius* to lack the strepsirrhine state for all of these three characters, analysis of the character matrix yields a single most parsimonious tree in which *Darwinius* is a basal stem-strepsirrhine while *Notharctus* is more nested. We think this is unlikely, given previously noted similarities between European adapids and strepsirrhines specifically e.g., [47]. Coding *Darwinius* as having the strepsirrhine condition for fibular facet slope (Char21), having a grooming claw (Char25), having a laterally placed flexor fibularis groove on the astragalus (Char31), but lacking a posterior trochlear shelf of the astragalus (similar to *Afradapis* [59]), results in three most parsimonious trees in which the consensus shows a monophyletic strepsirrhine clade where Lemuroidea, Lorisoidea, *Darwinius* and *Notharctus* form a polytomy. This is perhaps a reasonable reflection of the current state of knowledge on the inter-relationships among these taxa.

## Discussion

### Grooming claws

The foot of *Notharctus* AMNH 143612 is prosimian-like in having strong morphological evidence for a functional grooming claw on its second pedal digit. The distal phalanx has a similarly, physiologically dorsiflexed shaft (low FSA); a proximally restricted volar feature, indicating a shaft that projects far beyond the apical pad; and a smaller, more proximally positioned flexor tubercle, indicating reduced size and power of the flexor tendon (Figs. 6, 7, 8, 9). These features indicate a phalanx that was not critical for adding power to the grasp, but instead had a dorsally projecting unguis that could be used in grooming (e.g., scratching of the fur around the head and neck). Thus, using a diagnosis based on functional attributes; its morphology indicates the presence of a grooming claw. In contrast, the total morphological pattern in this bone is not identical to extant grooming phalanges, because the apical tuft is relatively very wide indicating the attachment of a keratinous structure that was flat and wide (at least at its base), more like the condition of extant ungular phalanges than extant grooming phalanges (Fig. 13).

This finding essentially obscures current hypotheses on the transformational sequence between primitive falcular phalanges and the ungular phalanges of living primates. Gingerich et al. [8] felt the sequence was clear to the degree that they coded falculae and grooming claws as equivalent structures in a character matrix for cladistic analysis. In this scenario, grooming claws would be essentially retained, and possibly slightly modified versions of, primitive falculae. In fact, *Notharctus* grooming phalanx morphology might suggest that this implied transformational sequence is correct, and that *Notharctus* represents a transitional step towards the anthropoid condition. Accepting this perspective justifies coding *Notharctus* as lacking a grooming claw, as we did in some iterations of our cladistic analyses presented above and in Appendix S1. The major issue with this interpretation is the recently demonstrated presence of grooming claws in certain ceboid primates [38], which is most parsimoniously interpreted as evidence that the earliest anthropoids had grooming claws. This would render the lack of a grooming claw in any adapiform a convergent similarity to anthropoids. Alternatively, the dp2 of *Notharctus*, with a grooming claw-like base, but a wide apical tuft, presents the possibility that the ancestral euprimate had ungular–like phalanges on all digits and that grooming claws were later evolved in various clades of primates e.g., [60], perhaps in settings where environments or social structure made mutualistic grooming by conspecifics insufficient. Assuming grooming claw morphology is generally primitive when encountered, one may take the position that grooming claws have re-evolved in platyrrhines, in order to allow for homology between the ungular form of the *Notharctus* dp2 apical tuft and that of catarrhine dp2's. Taking this position, however, actually adds plausibility to the interpretation that grooming claws in other primate groups also evolved from an ungular-type phalanx, and thereby, still presents a weak argument for dp2 similarities in any adapiform and anthropoids being synapomorphies to the exclusion of other major groups of primates.

Finally, it is not clear from published images that *Darwinius* lacked features characterizing a functional grooming claw. The mere presence of an apical tuft is not completely diagnostic of a nail-bearing distal phalanx; extant primate grooming phalanges, as well as tegulae, also possess apical tufts, albeit unexpanded apical tufts [38]. In order to truly diagnose the presence or absence of this condition in fossil adapiforms, a lateral view of the distal phalanx in question would be essential.

### Pedal proportions

For the most part, the new foot of *Notharctus* does not present any surprises relative to what would have been predicted from known specimens e.g., [9]. However, confirmed identification of bones belonging to particular digit rays and a broad comparative sample has allowed a more explicitly detailed view. Comparison to extant primates shows *Notharctus* to be most similar to prosimians, specifically lemuroids (Figs. 15, 16). On the other hand, *Darwinius* appears to be closer to the part of the distribution occupied by galagos (Fig. 16). Interestingly, although *Lepilemur* has often been the analogue of choice for the foot of *Notharctus* [9], [55], cheirogaleiids seem to show the closest overall similarity in our analyses.

The fourth digit would likely have been the longest of the foot, based on the size of pp4. Additionally, *Notharctus* exhibits relatively greater reduction of its pp2 compared to its pp5 than any anthropoid sampled (Fig. 15) and as compared to many individual lemuroids. This is also likely related to the co-option of the pedal digit two for grooming purposes more than grasping purposes. Notably, *Darwinius* exhibits even more pronounced reduction of pp2. This, to us, hints that a functional grooming phalanx was, in fact, present in *Darwinius* despite the wide apical tuft on its dp2. Reduction of the inner digits and increased size of the outer digits is a characteristic of what Morton [55] refers to as the “clinging or perching grasp,” but which also fits the description of the I–V grasp of Gebo [61]. Gebo [61] recognized adapiforms as possessing the I–V grasp based on their tarso-metatarsal pattern. Because our results indicate the greatest similarity of *Notharctus* to cheirogaleiids, instead of *Lepilemur*, it is interesting to note that cheirogaleiids exhibit the I–V grasp pattern, but *Lepilemur*, does not [61]. Additional features of the phalanges of the fifth digit of *Notharctus* suggest this digit was employed powerfully in grasping. These features include shafts that are robust; strongly curved with well-developed flexor-sheath ridges; and a large degree of lateral deviation in the shaft of the fifth intermediate phalanx.

### Modified version of Gingerich et al.'s character matrix

The result of analyzing this modified matrix prior to adding codings of *Notharctus* and *Catopithecus* already showed significant changes of topology (Fig. 17A), namely that *Darwinius* falls outside of crown Haplorhini. Adding fossil taxa to this modified matrix, results in trees in which both *Darwinius* and *Notharctus* occupy positions as stem-haplorhines and Anthropoidea including *Catopithecus* is monophyletic (Fig. 17B).

Reconstructing *Darwinius* and *Notharctus* as stem-haplorhines implies that omomyiforms (regarded as members of Tarsioidea) are closer to Anthropoidea than any adapiform. Most of the characters previously argued to support a close relationship between anthropoids and adapiforms would then be most parsimoniously interpreted as convergences. These convergent similarities include, specifically, fused/partially fused mandibular symphyses, spatulate vertical incisors, dimorphic canines, a short rostrum, and – if future studies confirm the lack of a grooming claw in *Darwinius*, this anthropoid similarity would also be convergent.

### Modified version of Gingerich et al.'s character matrix with additional characters

Results of our final analyses (Fig. 18) that include additional characters with states representing additional strepsirrhine, haplorhine, and anthropoid synapomorphies (Table 10), indicate that a stem haplorhine or stem anthropoid position for adapiforms does not best explain the data as we currently understand it. These analyses unambiguously suggest that adapiforms are closest to extant strepsirrhines (whereas tarsioids+anthropoids form the haplorhines) and they broadly support previously published hypotheses e.g., [2], [28], [29], [30], [31], [32], [33].

We hope that this cladistic analysis provides a step in the right direction for the controversy on adapiform affinities rekindled by *Darwinius*, as it represents a compromise in analytic framework. It has a minimum of missing data and follows the “few taxa, few characters” philosophy articulated by Gingerich et al. [8], but also includes critical information on other “transitional” fossils besides *Darwinius*. More fossils of early euprimates and new studies of morphological variation will continue to test the results and conclusions presented here. Finally, our results add new perspective on some of the current disagreements centered on the morphological evidence used to evaluate the phylogenetic position of adapiforms [2], [7], [8] by bringing to bear new anatomical detail within a methodological framework that addresses criticisms of both sides of the debate.

## Materials and Methods

### Preparation and documentation of associations

Prior to preparation of any kind, all blocks of AMNH 143612 from the original cabinet drawer (5.132B.02) and AMNH 143640 from cabinet drawer 5.132D.07 were CT-scanned at Stony Brook University Medical Center using a 64-source medical CT scanner (z = 0.625 mm). Observation of these scans allowed visualization of *in situ* skeletal elements, and confirmed that only two blocks among the assortment included pedal elements, or significantly complete elements of the skeleton. We feel it is likely that more of this specimen was preserved in the museum's collection. However, any such additional bones will not have strictly confirmable associations like those described here. The CT scan provided a permanent 3-dimensional record of original positions of what is a semi-articulated partial left pes (Figs. 3, 4). Physical preparation was conducted next. When all preserved elements were exposed within the matrix, a surface mold was made from a tin-based RTV silicone before disarticulation commenced. A polymer cast of the surface mold and a digital surface reconstruction (in 3d pdf format, Appendix S2, S3, and S4) of the CT scan data for both AMNH 143612 as well as AMNH 143640 were labeled with disarticulation numbers to document position and sequence of removal. Photographs were taken throughout the preparation process. Once physical specimens were removed, they were microCT-scanned at the AMNH. A final 3d pdf of elements in articulation is also included (Appendix S5).

### Comparative morphology methods

#### Comparisons of distal phalanx shape among extant and fossil euprimates

An extant comparative sample assembled previously for the purpose of assessing the degree to which the morphology of the second pedal digit of prosimians is distinguishable from that of anthropoids and non-primates [38] was employed here to assess morphological affinities of the distal phalanges of *Notharctus*. Forty-eight distal phalanx specimens from one individual of each of twenty-nine species were measured (Table S1). This sample includes falcular phalanges (unguals of non-primate mammals which bear falculae or claws), grooming phalanges (primate unguals which bear grooming claws), ungular phalanges (primate unguals which bear ungulae or nails), and tegular phalanges (the unguals of callitrichine primates which bear tegulae, medio-laterally compressed claw-like ungues [62], [63]). Some platyrrhine monkeys have been shown to possess grooming claws [1], [39], [40], [41], [64]. Therefore, that of *Aotus* is included in this analysis. The second pedal ungual of *Callicebus* is also included, but as it shares a unique set of similarities with both grooming and ungular phalanges, it is not placed in either group. Additionally, the unguals of several *Notharctus* specimens were also analyzed. One of these unguals, AMNH 11474 belonging to *Notharctus tenebrosus*, was already included in the study of Gregory [9]. Two other unguals (AMNH129382-A and AMNH 129382-B) were associated with a tray of pedal elements representing a largely undescribed partial skeleton of *Notharctus tenebrosus*. This is the first study to include the two pedal phalanges from AMNH 129382.

Ungual measurements were taken from microCT reconstructions or digital photographs of specimens using the software SigmaScan 5.0. Extant specimens that were microCT-scanned were scanned at Stony Brook University using a Scanco VivaCT75 MicroCT scanner at a resolution of 39 microns. The software Amira 5.2.0 was used to generate surface reconstructions and to export the images which were measured using SigmaScan. A set of nine measurements were taken on each ungual [38]. Ungual phalanges are comprised of two main parts: the base which houses the proximal articular facet, and the more distal shaft. Two measurements were taken on the base: base height (BH) and base width (BW). BH is taken perpendicular to the proximo-distal axis (the axis which runs from the inferior margin of the articular facet to the distal-most tip of the phalanx) and BW is taken parallel to the medio-lateral axis (perpendicular to the proximo-distal axis). Measures of width and height were also taken at two positions along the shaft: ¼ of its length (SH-1/4, SW-1/4) and ¾ of its length (SH-3/4, SW-3/4). The total length of the phalanx (TPL) along the proximo-distal axis was also recorded. Unguals vary in the degree to which the shaft is flexed or canted in relation to the base (i.e., the shaft may project dorsally or volarly with respect to the base). This cant of the shaft was quantified using a measurement of facet-shaft angle (FSA): the angle between the proximo-distal axis of the ungual and a line which runs from the superior margin of the articular facet to the inferior margin. Unguals also vary in the degree to which the apical pad extends along the shaft of the phalanx. The apical pad may extend for the entire length of the phalanx or it may only extend for a small portion of the phalanx's length, in which case the shaft of the phalanx projects distally beyond the digit tip. The extent of the apical pad along the volar surface of the distal phalanx is associated with a bony volar feature [38]. In falcular phalanges, this structure is a well-developed volar process that runs along a portion of the ungual's volar surface. In ungular phalanges, the extent of the apical pad is marked by an angle of the volar surface in which the volar surface faces more distally rather than volarly. Grooming and tegular phalanges also possess a structure similar to that of the volar process of falcular phalanges. The measurement, volar feature length (VFL), is the length of this feature along the proximo-distal axis of the ungual. Measurements of width are taken in dorsal view while all other measurements are taken in lateral view. For more details on these measurements see Maiolino et al. [38].

Non-angular measurements of unguals were converted to size-adjusted shape variables through division of the geometric mean [65]. One sample Kolmogorov-Smirnov tests show that data are normally distributed. Measurements were analyzed using a combination of principal component analysis (PCA) and multivariate and univariate statistics. MANOVA and ANOVA were used to assess significant differences among ungular groups, while a post hoc Hotelling's pairwise comparison and Tamhane's T2 tests were used to test for significant differences between the ungulae and grooming claw groups. Fossil specimens were compared to extant groups using a special case of the two-sample *t*-test in which a single specimen is compared with a sample. The formula for the *t*-statistic used in this test can be found on page 228 of Sokal and Rohlf [66]. ANOVAs, Tamhane's T2 tests, and *t*-tests were considered significant when p<0.0042 (Bonferroni corrected alpha of 0.05 for twelve sequential comparisons). Statistical analyses were performed using PAST v 2.03 and SPSS v17.0; the calculator functions of Microsoft Excel 2007 were used to calculate the aforementioned *t*-statistic.

#### Comparisons of foot proportions among extant and fossil euprimates

A dataset including maximum proximodistal lengths of all metatarsals, proximal phalanges and intermediate phalanges from 288 individuals of 39 species of extant primates was compiled (Table S2). Specimens were sampled from a number of institutions: AIZU, AMNH, BMNH, BC, CMNH, DUPC, FMNH, MCZH, MNHN, MNHU, NMNH, RMNH, SBU, and UNSM (See Table 1 for abbreviations). Prosimians and callitrichine measurements in this dataset were provided by P. Lemelin. Length measurements were taken on articulated specimens or, in some cases, on X-rays of skeletons preserved within skins (pers. comm. P. Lemelin). The length of all metatarsals, proximal, and intermediate phalanges were taken along the mid-line of each element. Data for *Notharctus* AMNH 143612 was obtained by caliper measurements on the physical specimens after preparation. Data for *Darwinius* was obtained from supplementary tables 16, 18, and 20 of Franzen et al. [6]. We found some discrepancies in these datasets that had to be dealt with and we cannot be 100% confident that we have chosen the correct adjustment. Primarily, there appears to be a problem with the length given for mt4 and mt5 of *Darwinius*. Mt4 is listed in the table as “12.3/12.3” whereas mt5 is listed as “-/15.0” We could find no explanation of the conventions used in Franzen et al.'s supplementary tables and do not know why the number “12.3” is repeated, yet “15.0” is not. No primate in our extant database has such a relatively short mt4. All individuals have an mt4 that is nearly as long as or longer than mt5, not dramatically shorter. We suspect some type of error, and conservatively replace “12.3” with a value of “14.55” (which was determined as the most conservative possible estimate given our comparative datasets; see Appendix S1, section 2) for our analyses.

We conducted both multivariate and univariate analyses comparing shape differences among extant animals and fossils. In any analysis, we used, at most, measurements from mt1-5, pp1-5, and ip2-3,5, as these are the only bones available in AMNH 143612 and 143640 (Table 2). Measurements were transformed in two ways to represent proportions. For multivariate analyses we standardized each measurement to the geometric mean of all measurements. Multivariate analyses of this study are represented by two discriminant function analyses (DFA) used to assess the overall phenetic affinities of the foot of *Notharctus* and *Darwinius* among primates. Extant primates were designated as belonging to one of nine groups for the DFA. These groups included Galagidae, Lorisidae, Daubentoniidae, Cheirogaleiidae, Lepilemuridae, Lemuridae, Indriidae, Tarsiidae, and Anthropoidea (Table S2). The first DFA used all thirteen variables (Table S3, S4). We computed classification success rates using a jack-knifing procedure in SPSS v17.0. The second DFA did not use any information on metatarsals and thus consisted of seven variables (a new geometric mean was calculated for this analysis). The purpose of the second analysis was to remove influence of proportions related to the possession of a tarsifulcrumating foot in *Notharctus* (in which it is prosimian-like) [9], [55].

Finally we computed and compared six indices. These include pp4/mt4, a measure of the prehensility e.g., [67], [68] of the foot and mt1/mt2, a measure of the hypertrophy of the hallux. These first two variables can be interpreted as distinguishing between tarsifulcrimating and metatarsifulcrumating feet (great prehensility and a larger hallux are expected in the tarsifulcrumating foot of prosimians). We additionally computed the ratio of mt3/mt4 and the ratio of mt4/mt5. The first metric is meant to reveal information about where the axis of the foot is. The second was computed to determine the typical proportion in primates, as values reported for *Darwinius*' mt4 seemed out of proportion to us. We also computed the ratio of pp2/pp5 positing that taxa with a grooming claw would rely on the outer digits more and thus have a lower value. Likewise in computing a sixth ratio (pp3/pp4), we posited that this value would also be affected by position of the foot axis, and in the case of tarsiers, the presence of a grooming claw on pp3. Thus, we predicted lower values in prosimians as compared to anthropoids. We used two-sample *t*-tests to compare prosimian and anthropoid distributions for the first four ratios (pp4/mt4, mt1/mt2, mt3/mt4, and mt4/mt5) after determining that tarsiers are subsumed within the strepsirrhine range of values. However the last two ratios (pp2/pp5 and pp3/pp4) showed distinctive differences among different prosimian clades leading us to use ANOVAs and Kruskal-Wallis tests to asses differences among 5 groups (tarsiers, lorises, galagos, lemuroids, and anthropoids). We then compared the values of *Notharctus* and *Darwinius* in each of these variables to the means of extant distributions, using two-sample *t*-tests as previously described. Significance of statistical tests was assessed using Bonferroni adjusted alphas for multiple comparisons.

### Cladistic analysis methods

Cladistic analyses were run in PAUP 4.0b10 on nexus files edited in Mesquite. We obtained the character matrix of Gingerich et al. [8] from their supplementary document as a starting point (see Results for details of checking, changing and adding to this matrix). For all analyses we recorded various tree statistics including tree length (or number of steps); Consistency index (CI); Homoplasy index (HI); Rescaled consistency index (RC); and Retention index (RI). All analyses employed an exhaustive search for the most parsimonious trees with Tupaioidea assigned as the outgroup and all characters considered ordered (except Char. #25; see Appendix S1).

## Supporting Information

Figure S1 **Trees from cladistic analysis using original matrix of Gingerich et al.** [**8**] **and additional taxa.** A, Consensus of three most parsimonious trees resulting from analysis with *Notharctus* scored as having a grooming claw. B, Single most parsimonious tree resulting from analysis with *Notharctus* scored as lacking a grooming claw. C, Consensus of three most parsimonious trees resulting from analysis with *Notharctus* scored as having a grooming claw. D, Majority Rules consensus of seven most parsimonious trees resulting from analysis with *Notharctus* scored as lacking a grooming claw. See Appendix S1, sections 6–9 for matrices used to generate these trees.
(TIF)

Figure S2 **Boxplot illustrating differences in mandibular corpus depth among anthropoid and prosimian taxa.** Note that *Darwinius*, *Notharctus*, and *Catopithecus* all plot within the extant prosimian (“shallow”) range.
(TIF)

Figure S3 **Trees from cladistic analysis using corrected matrix of Gingerich et al.** [**8**] **with** ***Notharctus*** **added.** A, Consensus of three most parsimonious trees resulting from analysis with *Notharctus* coded as having a grooming claw and with *Darwinius* coded as either unknown for this trait or as lacking a grooming claw (see Appendix S1, sections 11–12 for nexus files). B, Consensus of four most parsimonious trees resulting from analysis with *Notharctus* and *Darwinius* coded as lacking grooming claws (see Appendix S1, section 13 for nexus file).
(TIF)

Table S1 **Extant distal phalanx sample.** Extant sample analyzed in comparisons of distal phalanx shape. See Table 1 for abbreviations.
(DOC)

Table S2 **Extant phalangeal proportions sample.** Extant sample analyzed in comparisons of phalangeal proportions. Table A, Strepsirrhines; Table B, Haplorhines. See Table 1 for abbreviations.
(DOC)

Table S3 **Means and standard deviations from phalangeal proportions analyses.** Means (x) and standard deviations (s.d.) of shape variables used in two discriminant function analyses. Variables ending in ‘V’ were used in the first analysis; those ending in ‘Vv’ were used in the second (See Materials and Methods and Results). Table A, Means and standard deviations of groups discriminated among, along with fossil values; Table B, Means and standard deviations of strepsirrhine species used in the analyses, along with fossil values; Table C, Means and standard deviations of haplorhine species used in the analyses.
(DOC)

Table S4 **Standardized canonical discriminant function coefficients.** Coefficients from two discriminant function analyses. A, Discriminant function analysis of metatarsal and phalanx shape variables; B, Discriminant function analysis of phalanx shape variables only. See Table S3 for variable means and standard deviations.
(DOC)

Table S5 **Mandibular depth of fossil and extant primates.** Measurements are reported in millimeters. See Appendix S1 section 2 for details.
(DOC)

Table S6 **Gingerich et al. (2010) character matrix with our corrections and updates.** Missing data, ‘?’ are highlighted in yellow. See Appendix S1, sections 2–5 for details on corrections, additional characters, and additional fossil taxa; and section 16 for the text of the corresponding nexus file.
(DOC)

Appendix S1 **Supplementary documentation of phylogenetic analyses.**
(DOC)

Appendix S2 **3D pdf of AMNH 143612** ***in situ*** **elements.** Digital surface reconstruction of the CT scan data with labeled elements.
(PDF)

Appendix S3 **3D pdf of AMNH 143640** ***in situ*** **elements.** Digital surface reconstruction of the CT scan data with labeled elements.
(PDF)

Appendix S4 **High-resolution 3D pdf of all** ***in situ*** **elements of foot.** Reconstruction of *in situ* elements after MicroCT scanning individual bones and re-orienting them in their original positions.
(PDF)

Appendix S5 **High-resolution 3D pdf of all elements of foot in articulation.** Repositioning of MicroCT scans of individual bones into anatomical position.
(PDF)
