# Supplementary figures and images for: Evidence for a Grooming Claw in a North American Adapiform Primate: Implications for Anthropoid Origins
Source: PLoS One. 2012 Jan 10;7(1):e29135. doi: 10.1371/journal.pone.0029135 (PMC3254620; doi:10.1371/journal.pone.0029135)

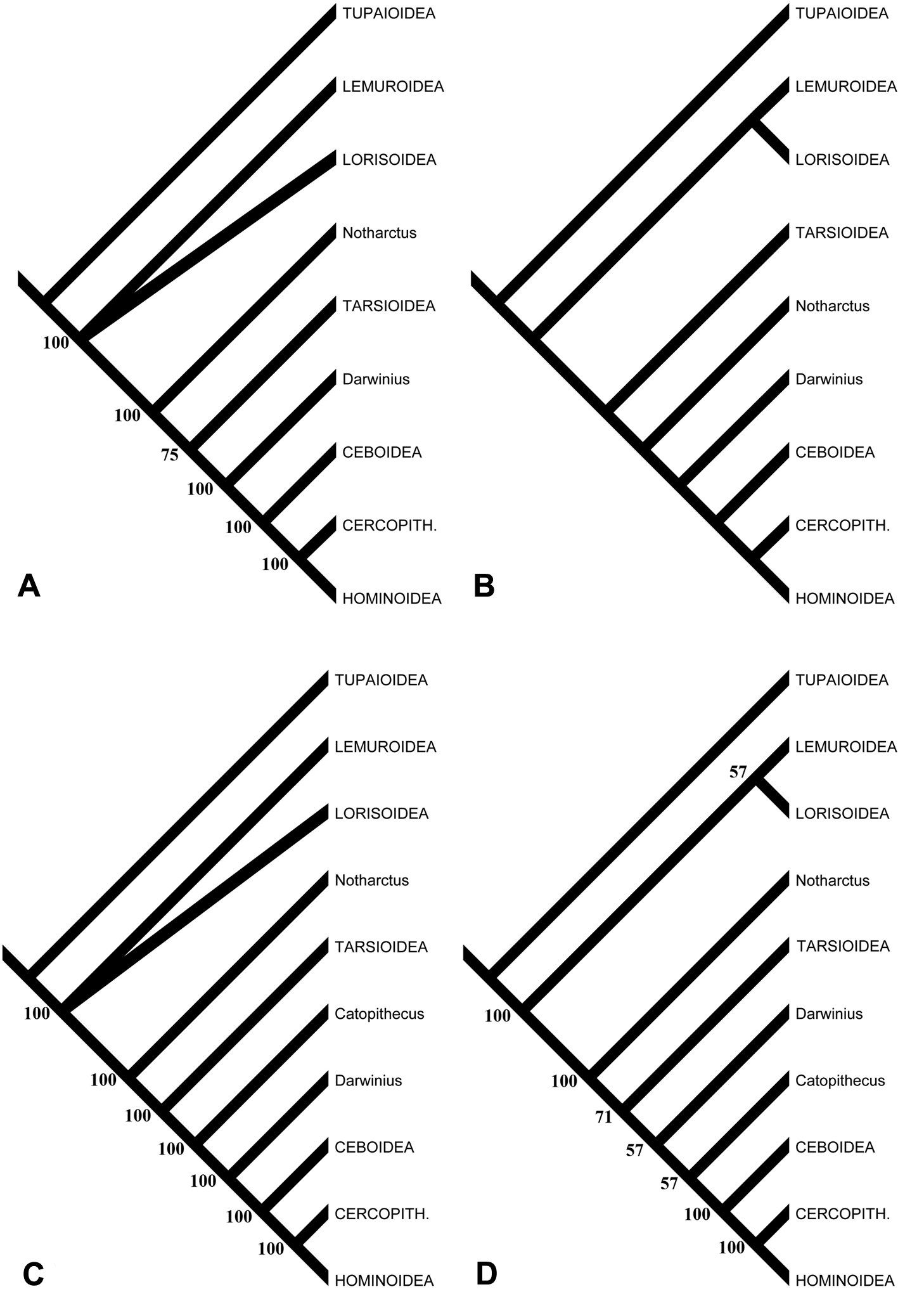

Supplement: Figure S1 — Trees from cladistic analysis using original matrix of Gingerich et al. [8] and additional taxa. A, Consensus of three most parsimonious trees resulting from analysis with Notharctus scored as having a grooming claw. B, Single most parsimonious tree resulting from analysis with Notharctus scored as lacking a grooming claw. C, Consensus of three most parsimonious trees resulting from analysis with Notharctus scored as having a grooming claw. D, Majority Rules consensus of seven most parsimonious trees resulting from analysis with Notharctus scored as lacking a grooming claw. See Appendix S1, sections 6–9 for matrices used to generate these trees. (TIF) [file pone.0029135.s001.tif]

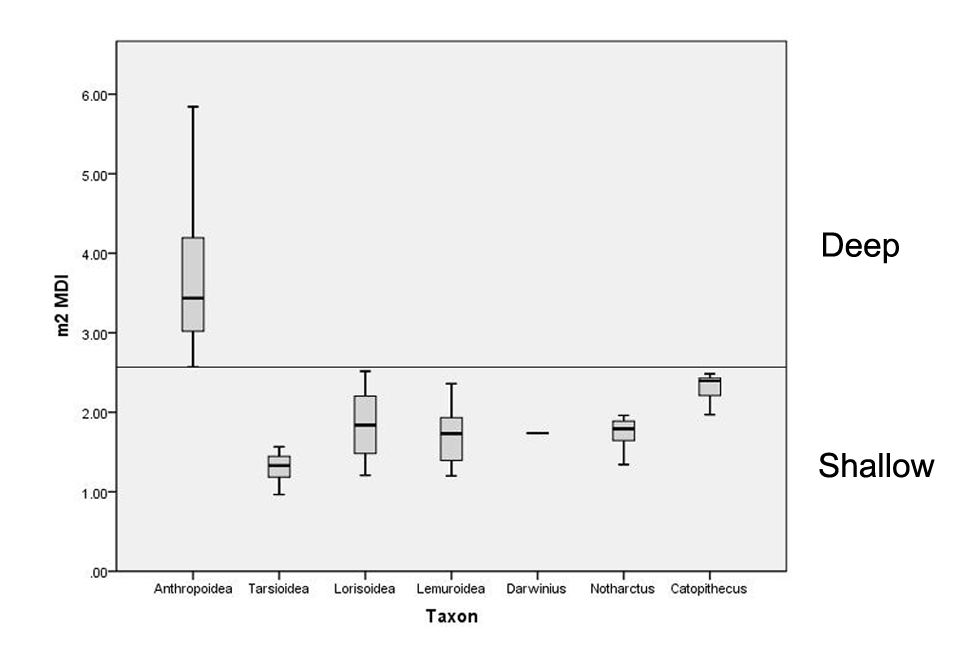

Supplement: Figure S2 — Boxplot illustrating differences in mandibular corpus depth among anthropoid and prosimian taxa. Note that Darwinius, Notharctus, and Catopithecus all plot within the extant prosimian (“shallow”) range. (TIF) [file pone.0029135.s002.tif]

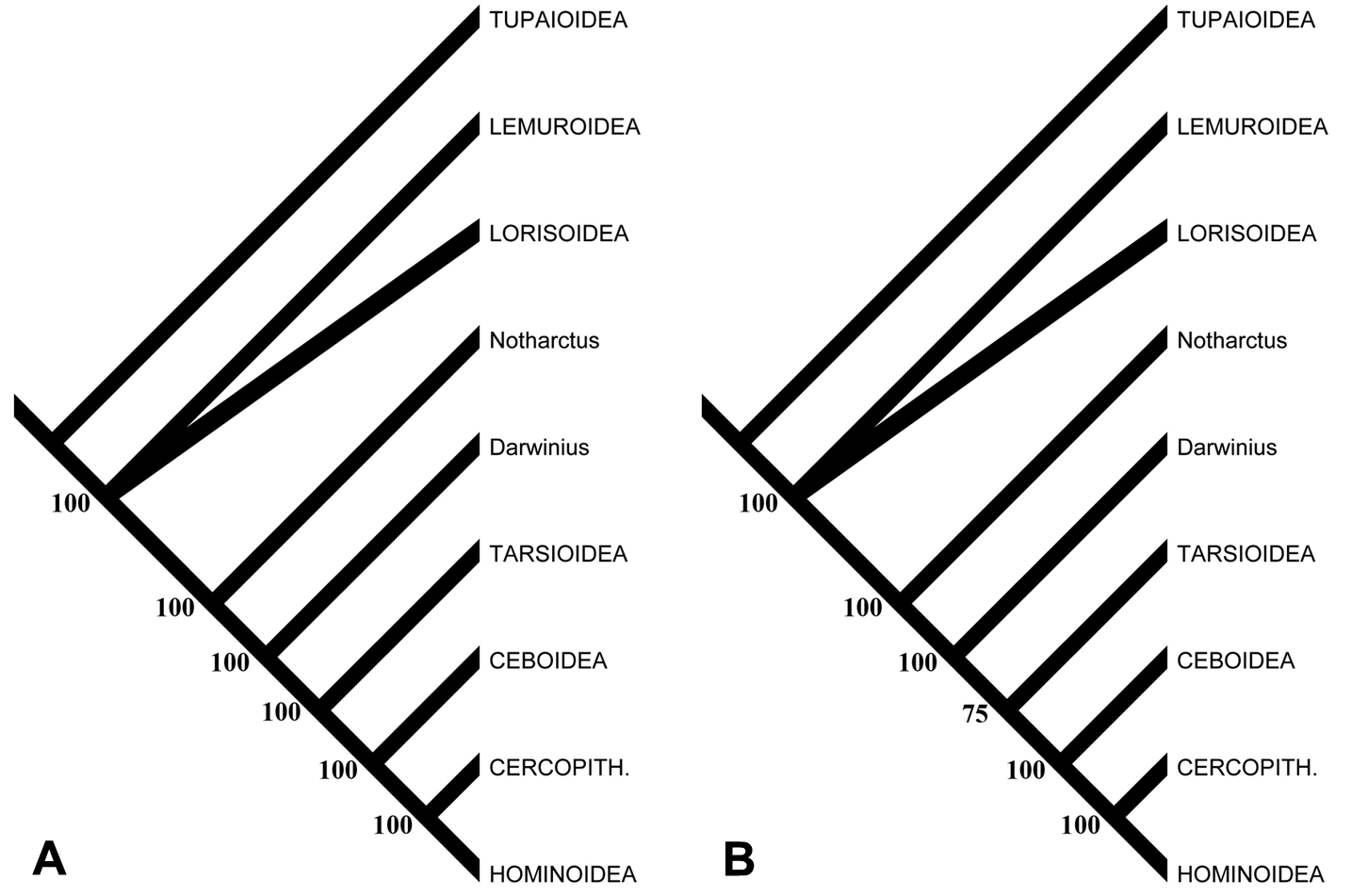

Supplement: Figure S3 — Trees from cladistic analysis using corrected matrix of Gingerich et al. [8] with Notharctus added. A, Consensus of three most parsimonious trees resulting from analysis with Notharctus coded as having a grooming claw and with Darwinius coded as either unknown for this trait or as lacking a grooming claw (see Appendix S1, sections 11–12 for nexus files). B, Consensus of four most parsimonious trees resulting from analysis with Notharctus and Darwinius coded as lacking grooming claws (see Appendix S1, section 13 for nexus file). (TIF) [file pone.0029135.s003.tif]

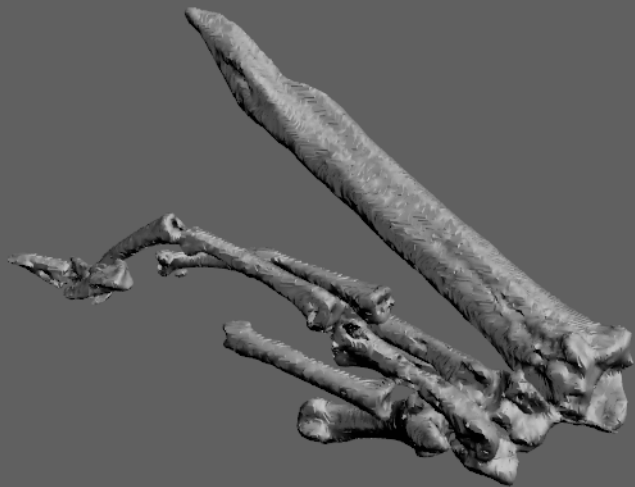

Supplement: Appendix S2 — 3D pdf of AMNH 143612 in situ elements. Digital surface reconstruction of the CT scan data with labeled elements. (PDF) [file pone.0029135.s011.pdf]

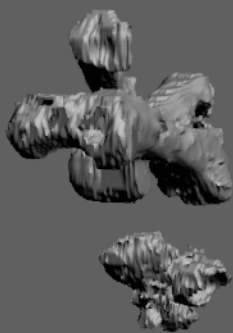

Supplement: Appendix S3 — 3D pdf of AMNH 143640 in situ elements. Digital surface reconstruction of the CT scan data with labeled elements. (PDF) [file pone.0029135.s012.pdf]

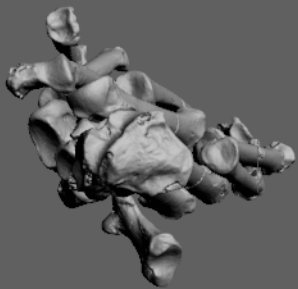

Supplement: Appendix S4 — High-resolution 3D pdf of all in situ elements of foot. Reconstruction of in situ elements after MicroCT scanning individual bones and re-orienting them in their original positions. (PDF) [file pone.0029135.s013.pdf]

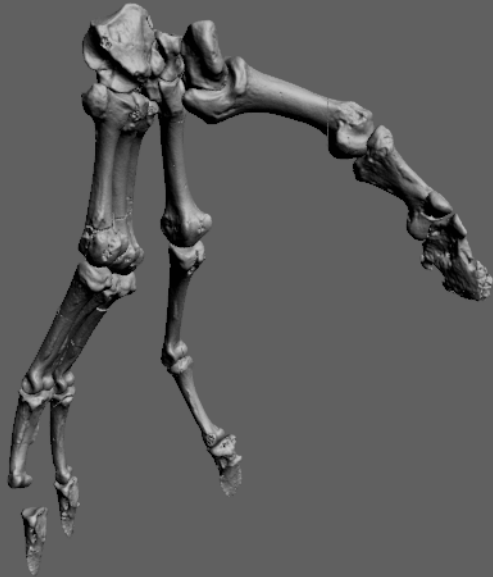

Supplement: Appendix S5 — High-resolution 3D pdf of all elements of foot in articulation. Repositioning of MicroCT scans of individual bones into anatomical position. (PDF) [file pone.0029135.s014.pdf]
