# Supplementary material for: Evidence for a Grooming Claw in a North American Adapiform Primate: Implications for Anthropoid Origins
Source: PLoS One. 2012 Jan 10;7(1):e29135. doi: 10.1371/journal.pone.0029135 (PMC3254620; doi:10.1371/journal.pone.0029135)
Supplement: Table S1 — Extant distal phalanx sample. Extant sample analyzed in comparisons of distal phalanx shape. See Table 1 for abbreviations. (DOC) [file pone.0029135.s004.doc]

**Table S1. Extant distal phalanx sample.**

| **Falculae** | | | |
| --- | --- | --- | --- |
| **Species (Taxon)** | **Specimen Number** | | **Ray** |
| *Didelphis sp.* (Didelphimorphia) | SBU* (3) | | 3^rd^ |
| *Phalanger orientalis* (Diprotodontia) | AMNH 79864 | | 4^th^ |
| *Tamandua sp.* (Pilosa) | SBU (1) | | 3^rd^ |
| *Suricata suricatta* (Carnivora) | AMNH 90441 | | 3^rd^ |
| *Sciurus sp.* (Rodentia) | SBU (2) | | 3^rd^ |
| *Galeopterus variegatus* (Dermoptera) | UNSM 15502 | | 3^rd^ |
| *Tupaia glis* (Scandentia) | AMNH 215175 | | 3^rd^ |
| **Grooming claws** | | | |
| *Eulemur fulvus* (Strepsirrhini - Lemuriformes) | SBU (13) | 2^nd^ | |
| *Hapalemur griseus* (Strepsirrhini - Lemuriformes) | SBU (12) | 2^nd^ | |
| *Lemur catta* (Strepsirrhini - Lemuriformes) | SBU (14) | 2^nd^ | |
| *Varecia variegata* (Strepsirrhini - Lemuriformes) | AMNH 201384 | 2^nd^ | |
| *Galago senegalensis* (Strepsirrhini - Lorisiformes) | SBU (15) | 2^nd^ | |
| *Nycticebus coucang* (Strepsirrhini - Lorisiformes) | AMNH 16615 | 2^nd^ | |
| *Tarsius bancanus* (Haplorhini - Tarsiiformes) | AMNH 106754 | 2^nd^, 3^rd^ | |
| *Tarsius spectrum* (Haplorhini - Tarsiiformes) | AMNH 109367 | 2^nd^, 3^rd^ | |
| *Aotus sp.* (Haplorhini - Platyrrhini) | SBU (11) | 2^nd^ | |
| **Ungulae** | | | |
| *Eulemur fulvus* (Strepsirrhini - Lemuriformes) | SBU (13) | 3^rd^ | |
| *Hapalemur griseus* (Strepsirrhini - Lemuriformes) | SBU (12) | 3^rd^ | |
| *Lemur catta* (Strepsirrhini - Lemuriformes) | SBU (14) | 3^rd^ | |
| *Varecia variegate* (Strepsirrhini - Lemuriformes) | AMNH 201384 | 3^rd^ | |
| *Galago senegalensis* (Strepsirrhini - Lorisiformes) | SBU (15) | 3^rd^ | |
| *Nycticebus coucang* (Strepsirrhini - Lorisiformes) | AMNH 16615 | 3^rd^ | |
| *Tarsius bancanus* (Haplorhini - Tarsiiformes) | AMNH 106754 | 4^th^ | |
| *Tarsius spectrum* (Haplorhini - Tarsiiformes) | AMNH 109367 | 4^th^ | |
| *Ateles sp.* (Haplorhini - Platyrrhini) | SBU (10) | 2^nd^, 3^rd^ | |
| *Brachyteles arachnoids* (Haplorhini - Platyrrhini) | AMNH 260 | 2^nd^, 3^rd^ | |
| *Pithecia pithecia* (Haplorhini - Platyrrhini) | SBU (8) | 2^nd^, 3^rd^ | |
| *Callicebus cupreus* (Haplorhini - Platyrrhini) | AMNH 130361 | 2^nd**^, 3^rd^ | |
| *Aotus sp.* (Haplorhini - Platyrrhini) | SBU (11) | 3^rd^ | |
| *Cebus sp.* (Haplorhini - Platyrrhini) | SBU (7) | 2^nd^, 3^rd^ | |
| *Saimiri sp.* (Haplorhini - Platyrrhini) | SBU (9) | 2^nd^, 3^rd^ | |
| *Chlorocebus aethiops* (Haplorhini - Catarrhini) | SBU (5) | 2^nd^, 3^rd^ | |
| *Macaca sp.* (Haplorhini - Catarrhini) | SBU (4) | 2^nd^, 3^rd^ | |
| *Hylobates sp.* (Haplorhini - Catarrhini) | SBU (6) | 2^nd^ | |
| **Tegulae** | | | |
| *Callithrix sp.* (Haplorhini - Platyrrhini) | AMNH 22994 | 3^rd^ | |
| *Cebuella pygmaea* (Haplorhini - Platyrrhini) | AMNH 244101 | 3^rd^ | |
| *Leontopithecus sp.* (Haplorhini - Platyrrhini) | AMNH 235275 | 3^rd^ | |
| *Saguinus fuscicollis* (Haplorhini - Platyrrhini) | SBU (16) | 3^rd^ | |

*SBU specimens are not associated with numbers; provisional numbers follow in parentheses.

**The second pedal ungual of *Callicebus* is morphologically unique in its combination of similarities with grooming claws and ungulae and, therefore, is not placed in either group.

**Table Legend**

Extant sample analyzed in comparisons of distal phalanx shape. See Table 1 for abbreviations.
