# Supplementary material for: Evidence for a Grooming Claw in a North American Adapiform Primate: Implications for Anthropoid Origins
Source: PLoS One. 2012 Jan 10;7(1):e29135. doi: 10.1371/journal.pone.0029135 (PMC3254620; doi:10.1371/journal.pone.0029135)
Supplement: Table S2 — Extant phalangeal proportions sample. Extant sample analyzed in comparisons of phalangeal proportions. Table A, Strepsirrhines; Table B, Haplorhines. See Table 1 for abbreviations. (DOC) [file pone.0029135.s005.doc]

**Table S2. Extant phalangeal proportions sample.**

A. Extant strepsirhine sample.

| **Species** | **DFA** | **n** | **Specimen Numbers** |
| --- | --- | --- | --- |
| *Galago senegalensis* | 1 | 13/12 | AMNH169426, 119521, 244100, 35445; CMNH B747; NMNH 397967, 397968; FMNH 121213, 58976, 135741, 127371, 127375; BMNH 1952-7284 |
| *Galago moholi* | 1 | 7 | AMNH 86065, 86502, 86503, 86504, 86505; BMNH 1973-19, 1963-571 |
| *Galagoides demidoff* | 1 | 9 | AMNH 215180, 150413, 212956; FMNH 104804; BMNH 1982-483, 1982-484; AIZU 7654, 7656, 7657 |
| *Euoticus elegantulus* | 1 | 9 | BMNH 1977-889, 1948-535, 1948-532, 1948-541, 1977-886; AIZU 7672, 7673, 7679, 7628 |
| *Galagoides alleni* | 1 | 3 | NMNH 49548; AIZU 7534, 7634 |
| *Otolemur crassicaudatus* | 1 | 9 | AMNH 201330, 245093; NMNH 397989, 399062, 399060, 267588, FMNH 53077; RMNH 1893(1), 1893(2) |
| *Otolemur garnettii* | 1 | 7 | BMNH 1990-526; RMNH 1895; CMNH B748; DUPC 1055, 1034, 1082, 418m |
| *Arctocebus calabarensis* | 2 | 11 | AMNH 212954, 212576, 207949; BMNH 1962-1151; AIZU 7669, 7704, 7303, 7761, 7680, 7665, 7633 |
| *Perodicticus potto* | 2 | 13/12 | AMNH 200232, 86898, 52685; CMNH B1047, B751; NMNH 49547, 481744; BMNH 1977-880, 1977-1052; RMNH 6; AIZU 6538, 6623, 7575 |
| *Loris tardigradus* | 2 | 10 | AMNH 34257; CMNH B750; FMNH 58979; BMNH 1848-10313, 1948-10206, 1951-111026; RMNH 4, 5, 1490(1926); MNHU 20838 |
| *Nycticebus coucang* | 2 | 16 | AMNH 16591, 102027; CMNH B1039, B749, B136; NMNH 271186, 300000; FMNH 89467; MCZH 5118; BMNH 1961-621, 1896-11294, 1948-5121, 1977-2868; RMNH 27121865(9), 1875(11); MNHU 2718 |
| *Nycticebus pygmaeus* | 2 | 4/2 | FMNH 46825, 46828, 145466, 140917 |
| *Daubentonia madagascariensis* | 3 | 9 | AMNH 185643; CMNH B198; NMNH 199694, 305066; MNHN 1901-647, 1911-136; RMNH 531; MNHU 84264; AIZU 1843 |
| *Microcebus murinus* | 4 | 11 | AMNH 174385, 174424, 174500, 174471, 174131, 185630, 185628; CMNH B758*; NMNH 83655, 83656; BMNH 1892-1162, 1897-9128 |
| *Cheirogaleus medius* | 4 | 6 | RMNH 81870(13), 81870(15), 81870(12); MNHN 1975-127; DUPC 618m, 031 |
| *Cheirogaleus major* | 4 | 6 | NMNH 83653, 397231; MCZH 5117; MNHN 1888-819, 1903-297, 1884-2442 |
| *Lepilemur mustelinus* | 5 | 5 | NMNH 49668; MCZH 5110, 5095; BMNH 1897-9123, 1871-4311 |
| *Avahi laniger* | 6 | 9 | NMNH 83652, 83651; FMNH 5654; MCZH 5119; MNHN 1901-519; RMNH 1871878(2)*, 1868(6), 1876-I, 1876-II; MNHU 44674 |
| *Propithecus verreauxi* | 6 | 11 | AMNH 170491, 31255, 170463; MNHN 1932-486bis, 1939-330, 1932-486, 1961-61, A2839; MNHU 5648; AIZU 7255, AHS1819 |
| *Propithecus diadema* | 6 | 6 | CMNH 1155*; MCZH 6070-76, 5016; RMNH 1877, 1876; MNHU 84294, 44771 |
| *Indri indri* | 6 | 7 | CMNH B343; MNHN 1938-525, 1901-518, 1934-537; A3913; RMNH 1878, 1877 |
| *Eulemur macaco* | 7 | 6 | AMNH 201318; FMNH 121545; BMNH 1948-10185, 1961-624; RMNH 1867; MNHN 1887-448 |
| *Eulemur mongoz* | 7 | 6 | NMNH 49947, 35260; BMNH 1870-557; RMNH 20111876*; MNHN 1893-428, 1907-275, 1901-521 |
| *Eulemur fulvus* | 7 | 7 | AMNH 31254, 35753, 17403, 170755; NMNH 542489; BMNH 1948-10204, 1948-5127 |
| *Varecia variegata* | 7 | 10/9 | AMNH 201384, 22897; CMNH 1382; MCZH 59276; MNHN 1896-286, 1934-596, 1908-215, A3918, 1948-13; RMNH 1867 |
| *Lemur catta* | 7 | 12/11 | CMNH B197, B1157, 1051, B756, B1029; MCZH 6010*; MNHN 1909-289, 1906-311, 1896-379; BMNH 1948-10184, 1968-711; AIZU 8598 |
| *Hapalemur griseus* | 7 | 12 | NMNH 83666, 83667, 83668; MCZH 44913, 44910, 44911; BMNH 1881-670, 1870-554; RMNH 1531878(20), 421878(19), C; AIZU 5055 |

B. Extant haplorhine sample.

| **Species** | **DFA** | **n** | **Specimen Numbers** |
| --- | --- | --- | --- |
| *Tarsius spectrum* | 8 | 2 | RMNH 5064, 2863 |
| *Tarsius syrichta* | 8 | 10/9 | AMNH 206757; NMNH 282761; FMNH 142007, 129379, 57281, 56728; MNHN 1884-411; BMNH 97522; AIZU AS1855, AS1840 |
| *Tarsius bancanus* | 8 | 10 | CMNH B135, B1229, B1156; NMNH 574133, 546331, 546332; FMNH 76859; MCZH 6023*; BMNH 1878-3045; RMNH 14556, 14557 |
| *Saguinus midas* | 9 | 6 | MCZH 7110; AIZU 7823, 8676, 7794, 7657, 7163 |
| *Saguinus oedipus* | 9 | 5 | RMNH 1867(5); AIZU 8520, AS1797, AS1070, AS1778 |
| *Callithrix jacchus* | 9 | 5 | RMNH 1876, 1877; AIZU 6439, 6438, 6437 |
| *Leontopithecus rosalia* | 9 | 3 | MCZH 3939; RMNH 9, 1867(8) |
| *Aotus sp.* | 9 | 1 | Mounted Carolina Biological Supply specimen (BC) |
| *Aotus trivirgatus* | 9 | 1 | Mounted Carolina Biological Supply specimen (SBU) |
| *Alouatta sp.* | 9 | 1 | Mounted Carolina Biological Supply specimen (BC) |
| *Saimiri sciureus* | 9 | 5/3 | SBU N SM 6, N SM 5, N SM 10, N SM 9, N SM 8 |
| *Saimiri sp.* | 9 | 3/2 | SBU no number, N SM 2, N SM 7 |
| *Cebus sp.* | 9 | 1 | SBU N Cb 11 |
| *Cebus albifrons* | 9 | 1 | SBU N Cb 10 |
| *Cacajao rubicundus* | 9 | 1 | SBU N Cj 1 |
| *Chiropotes sp.* | 9 | 1/0 | SBU N Ch 2 |

*Specimens removed from final analyses because they were extreme outliers relative to other members of their species. These specimens are not included in the number of individuals per species, ‘n.’

‘DFA’ refers to group codes used in discriminant function analyses.

‘n’ refers to the number of individuals per species. A number following a ‘/’ indicates the number of individuals used in the DFA as specimens lack the data required by the DFA.

**Table Legend**

Extant sample analyzed in comparisons of phalangeal proportions. Table A, Strepsirhines; Table B, Haplorhines. See Table 1 for abbreviations.
