# Supplementary material for: Evidence for a Grooming Claw in a North American Adapiform Primate: Implications for Anthropoid Origins
Source: PLoS One. 2012 Jan 10;7(1):e29135. doi: 10.1371/journal.pone.0029135 (PMC3254620; doi:10.1371/journal.pone.0029135)
Supplement: Table S3 — Means and standard deviations from phalangeal proportions analyses. Means (x) and standard deviations (s.d.) of shape variables used in two discriminant function analyses. Variables ending in ‘V’ were used in the first analysis; those ending in ‘Vv’ were used in the second (See Materials and Methods and Results). Table A, Means and standard deviations of groups discriminated among, along with fossil values; Table B, Means and standard deviations of strepsirrhine species used in the analyses, along with fossil values; Table C, Means and standard deviations of haplorhine species used in the analyses. (DOC) [file pone.0029135.s006.doc]

**Table S3. Means and standard deviations from phalangeal proportions analyses.**

A. Means and standard deviations of groups discriminated among, along with fossil values.

| **Taxon** | **value** | **Mt1V*** | **Mt2V** | **Mt3V** | **Mt4V** | **Mt5V** | **pp1V** | **pp2V** | **pp3V** | **pp4V** | **pp15V** | **ip2V** | **ip3V** | **ip5V** |
| --- | --- | --- | --- | --- | --- | --- | --- | --- | --- | --- | --- | --- | --- | --- |
| *Notharctus* 143612 |  | 1.179 | 1.181 | 1.315 | 1.270 | 1.140 | 0.735 | 0.994 | 1.164 | 1.202 | 1.036 | 0.669 | 0.760 | 0.700 |
| *Darwinius masilla*e |  | 1.224 | 1.232 | 1.292 | 1.099 | 1.133 | 1.096 | 1.020 | 1.171 | 1.307 | 1.141 | 0.506 | 0.657 | 0.635 |
| Galagids | x | 1.304 | 1.259 | 1.324 | 1.258 | 1.193 | 0.836 | 0.945 | 1.192 | 1.359 | 1.068 | 0.466 | 0.737 | 0.663 |
| (1)** N=56 | s.d. | 0.052 | 0.049 | 0.054 | 0.058 | 0.065 | 0.029 | 0.038 | 0.032 | 0.058 | 0.055 | 0.037 | 0.048 | 0.038 |
| Lorisids | x | 1.235 | 1.192 | 1.251 | 1.227 | 1.178 | 0.850 | 0.902 | 1.276 | 1.468 | 1.254 | 0.410 | 0.726 | 0.735 |
| (2) N=51 | s.d. | 0.160 | 0.104 | 0.053 | 0.059 | 0.059 | 0.081 | 0.067 | 0.089 | 0.066 | 0.050 | 0.073 | 0.083 | 0.043 |
| Daubentoniids | x | 1.149 | 1.315 | 1.371 | 1.354 | 1.293 | 0.580 | 1.043 | 1.126 | 1.205 | 1.079 | 0.573 | 0.739 | 0.743 |
| (3) N=9 | s.d. | 0.020 | 0.036 | 0.033 | 0.055 | 0.055 | 0.019 | 0.020 | 0.033 | 0.030 | 0.023 | 0.011 | 0.017 | 0.078 |
| Cheirogaleids | x | 1.304 | 1.437 | 1.408 | 1.361 | 1.198 | 0.734 | 0.963 | 1.097 | 1.203 | 1.002 | 0.531 | 0.712 | 0.667 |
| (4) N=23 | s.d. | 0.071 | 0.106 | 0.042 | 0.040 | 0.038 | 0.025 | 0.034 | 0.029 | 0.061 | 0.035 | 0.040 | 0.051 | 0.038 |
| Lepilemurids | x | 1.367 | 1.530 | 1.573 | 1.527 | 1.372 | 0.719 | 0.991 | 1.081 | 1.185 | 0.948 | 0.483 | 0.641 | 0.548 |
| (5) N=5 | s.d. | 0.057 | 0.046 | 0.049 | 0.045 | 0.044 | 0.044 | 0.044 | 0.016 | 0.036 | 0.026 | 0.022 | 0.050 | 0.049 |
| Indriids | x | 1.462 | 1.524 | 1.569 | 1.592 | 1.507 | 0.756 | 0.962 | 1.036 | 1.134 | 0.991 | 0.450 | 0.572 | 0.558 |
| (6) N=33 | s.d. | 0.057 | 0.063 | 0.072 | 0.070 | 0.056 | 0.040 | 0.029 | 0.027 | 0.036 | 0.037 | 0.059 | 0.029 | 0.025 |
| Lemurids | x | 1.359 | 1.560 | 1.564 | 1.569 | 1.463 | 0.717 | 0.943 | 1.050 | 1.134 | 0.964 | 0.471 | 0.626 | 0.580 |
| (7) N=51 | s.d. | 0.061 | 0.037 | 0.037 | 0.054 | 0.052 | 0.028 | 0.029 | 0.022 | 0.023 | 0.036 | 0.037 | 0.024 | 0.032 |
| Tarsiids | x | 1.312 | 1.210 | 1.423 | 1.384 | 1.196 | 0.808 | 0.938 | 1.128 | 1.353 | 1.090 | 0.503 | 0.603 | 0.712 |
| (8) N=21 | s.d. | 0.043 | 0.048 | 0.042 | 0.034 | 0.038 | 0.023 | 0.038 | 0.037 | 0.060 | 0.059 | 0.052 | 0.053 | 0.043 |
| Anthropoids | x | 1.013 | 1.710 | 1.855 | 1.961 | 1.849 | 0.595 | 0.923 | 1.000 | 1.000 | 0.862 | 0.534 | 0.645 | 0.541 |
| (9) N=30 | s.d. | 0.074 | 0.087 | 0.124 | 0.156 | 0.137 | 0.042 | 0.045 | 0.025 | 0.037 | 0.040 | 0.047 | 0.045 | 0.042 |
| **Taxon** | **value** | **pp2Vv** | **pp3Vv** | **pp4Vv** | **pp5Vv** | **ip2Vv** | **ip3Vv** | **ip5Vv** |  |  |  |  |  |  |
| *Notharctus* 143612 |  | 1.094 | 1.280 | 1.322 | 1.140 | 0.736 | 0.836 | 0.770 |  |  |  |  |  |  |
| *Darwinius masilla*e |  | 1.173 | 1.347 | 1.503 | 1.312 | 0.582 | 0.756 | 0.730 |  |  |  |  |  |  |
| Galagids | x | 1.089 | 1.375 | 1.567 | 1.231 | 0.538 | 0.850 | 0.764 |  |  |  |  |  |  |
| (1)** N=60 | s.d. | 0.036 | 0.041 | 0.057 | 0.056 | 0.039 | 0.049 | 0.037 |  |  |  |  |  |  |
| Lorisids | x | 1.009 | 1.428 | 1.648 | 1.407 | 0.458 | 0.812 | 0.824 |  |  |  |  |  |  |
| (2) N=54 | s.d. | 0.056 | 0.064 | 0.122 | 0.070 | 0.068 | 0.067 | 0.042 |  |  |  |  |  |  |
| Daubentoniids | x | 1.159 | 1.252 | 1.339 | 1.199 | 0.637 | 0.822 | 0.825 |  |  |  |  |  |  |
| (3) N=9 | s.d. | 0.024 | 0.042 | 0.035 | 0.031 | 0.011 | 0.024 | 0.078 |  |  |  |  |  |  |
| Cheirogaleids | x | 1.134 | 1.292 | 1.416 | 1.181 | 0.625 | 0.838 | 0.785 |  |  |  |  |  |  |
| (4) N=23 | s.d. | 0.061 | 0.028 | 0.052 | 0.048 | 0.043 | 0.043 | 0.031 |  |  |  |  |  |  |
| Lepilemurids | x | 1.245 | 1.358 | 1.488 | 1.191 | 0.607 | 0.805 | 0.689 |  |  |  |  |  |  |
| (5) N=5 | s.d. | 0.051 | 0.029 | 0.051 | 0.043 | 0.026 | 0.052 | 0.058 |  |  |  |  |  |  |
| Indriids | x | 1.252 | 1.348 | 1.476 | 1.289 | 0.584 | 0.743 | 0.726 |  |  |  |  |  |  |
| (6) N=33 | s.d. | 0.050 | 0.051 | 0.062 | 0.045 | 0.069 | 0.031 | 0.026 |  |  |  |  |  |  |
| Lemurids | x | 1.200 | 1.337 | 1.445 | 1.227 | 0.600 | 0.798 | 0.738 |  |  |  |  |  |  |
| (7) N=55 | s.d. | 0.026 | 0.036 | 0.044 | 0.039 | 0.042 | 0.028 | 0.032 |  |  |  |  |  |  |
| Tarsiids | x | 1.098 | 1.321 | 1.584 | 1.276 | 0.588 | 0.706 | 0.833 |  |  |  |  |  |  |
| (8) N=22 | s.d. | 0.054 | 0.052 | 0.069 | 0.067 | 0.057 | 0.058 | 0.047 |  |  |  |  |  |  |
| Anthropoids | x | 1.214 | 1.316 | 1.316 | 1.134 | 0.701 | 0.847 | 0.712 |  |  |  |  |  |  |
| (9) N=30 | s.d. | 0.050 | 0.036 | 0.041 | 0.048 | 0.053 | 0.048 | 0.053 |  |  |  |  |  |  |

B. Means and standard deviations of strepsirhine species used in the analyses, along with fossil values.

| **Taxon** | **value** | **Mt1V** | **Mt2V** | **Mt3V** | **Mt4V** | **Mt5V** | **pp1V** | **pp2V** | **pp3V** | **pp4V** | **pp5V** | **ip2V** | **ip3V** | **ip5V** |
| --- | --- | --- | --- | --- | --- | --- | --- | --- | --- | --- | --- | --- | --- | --- |
| *Notharctus* 143612 |  | 1.179 | 1.181 | 1.315 | 1.270 | 1.140 | 0.735 | 0.994 | 1.164 | 1.202 | 1.036 | 0.669 | 0.760 | 0.700 |
| *Darwinius masillae* |  | 1.224 | 1.232 | 1.292 | 1.099 | 1.133 | 1.096 | 1.020 | 1.171 | 1.307 | 1.141 | 0.506 | 0.657 | 0.635 |
| *Galago senegalensis* | x | 1.314 | 1.303 | 1.349 | 1.296 | 1.198 | 0.819 | 0.926 | 1.171 | 1.320 | 1.016 | 0.479 | 0.739 | 0.665 |
| N=12 | s.d. | 0.033 | 0.046 | 0.047 | 0.036 | 0.049 | 0.023 | 0.018 | 0.029 | 0.041 | 0.020 | 0.028 | 0.034 | 0.031 |
| *Galago moholi* | x | 1.294 | 1.300 | 1.347 | 1.252 | 1.186 | 0.824 | 0.912 | 1.163 | 1.312 | 1.002 | 0.497 | 0.783 | 0.669 |
| N=7 | s.d. | 0.030 | 0.017 | 0.033 | 0.039 | 0.023 | 0.030 | 0.015 | 0.013 | 0.023 | 0.015 | 0.024 | 0.048 | 0.022 |
| *Galagoides demidoff* | x | 1.354 | 1.206 | 1.258 | 1.167 | 1.106 | 0.868 | 0.961 | 1.209 | 1.446 | 1.101 | 0.451 | 0.771 | 0.684 |
| N=9 | s.d. | 0.031 | 0.043 | 0.041 | 0.038 | 0.030 | 0.030 | 0.034 | 0.030 | 0.035 | 0.039 | 0.035 | 0.045 | 0.065 |
| *Euoticus elegantulus* | x | 1.233 | 1.231 | 1.303 | 1.243 | 1.168 | 0.828 | 1.007 | 1.212 | 1.370 | 1.141 | 0.473 | 0.716 | 0.657 |
| N=9 | s.d. | 0.044 | 0.020 | 0.036 | 0.031 | 0.040 | 0.025 | 0.011 | 0.015 | 0.028 | 0.037 | 0.044 | 0.041 | 0.018 |
| *Galagoides alleni* | x | 1.277 | 1.218 | 1.277 | 1.223 | 1.142 | 0.813 | 0.935 | 1.211 | 1.416 | 1.070 | 0.473 | 0.770 | 0.713 |
| N=3 | s.d. | 0.018 | 0.025 | 0.038 | 0.039 | 0.020 | 0.018 | 0.020 | 0.020 | 0.042 | 0.042 | 0.021 | 0.043 | 0.035 |
| *Otolemur crassicaudatus* | x | 1.323 | 1.270 | 1.381 | 1.310 | 1.269 | 0.841 | 0.928 | 1.193 | 1.326 | 1.056 | 0.455 | 0.694 | 0.635 |
| N=9 | s.d. | 0.051 | 0.038 | 0.029 | 0.032 | 0.051 | 0.015 | 0.030 | 0.028 | 0.045 | 0.021 | 0.044 | 0.018 | 0.022 |
| *Otolemur garnettii* | x | 1.310 | 1.253 | 1.320 | 1.285 | 1.256 | 0.850 | 0.935 | 1.204 | 1.364 | 1.097 | 0.434 | 0.711 | 0.649 |
| N=7 | s.d. | 0.040 | 0.024 | 0.024 | 0.022 | 0.019 | 0.020 | 0.025 | 0.042 | 0.031 | 0.030 | 0.010 | 0.038 | 0.021 |
| *Arctocebus calabarensis* | x | 1.471 | 1.225 | 1.270 | 1.291 | 1.224 | 0.976 | 0.816 | 1.138 | 1.512 | 1.209 | 0.393 | 0.619 | 0.698 |
| N=11 | s.d. | 0.075 | 0.034 | 0.037 | 0.050 | 0.081 | 0.036 | 0.033 | 0.041 | 0.027 | 0.039 | 0.035 | 0.050 | 0.055 |
| *Perodicticus potto* | x | 1.104 | 1.134 | 1.242 | 1.222 | 1.169 | 0.871 | 0.898 | 1.297 | 1.531 | 1.305 | 0.413 | 0.739 | 0.751 |
| N=12 | s.d. | 0.046 | 0.044 | 0.041 | 0.048 | 0.046 | 0.021 | 0.051 | 0.047 | 0.061 | 0.041 | 0.091 | 0.073 | 0.033 |
| *Loris tardigradus* | x | 1.343 | 1.351 | 1.308 | 1.232 | 1.190 | 0.819 | 0.892 | 1.262 | 1.414 | 1.240 | 0.344 | 0.710 | 0.737 |
| N=10 | s.d. | 0.035 | 0.049 | 0.037 | 0.037 | 0.038 | 0.028 | 0.034 | 0.030 | 0.042 | 0.036 | 0.045 | 0.019 | 0.030 |
| *Nycticebus coucang* | x | 1.119 | 1.122 | 1.224 | 1.198 | 1.158 | 0.775 | 0.962 | 1.353 | 1.431 | 1.258 | 0.449 | 0.789 | 0.744 |
| N=16 | s.d. | 0.028 | 0.077 | 0.037 | 0.035 | 0.038 | 0.035 | 0.044 | 0.037 | 0.042 | 0.040 | 0.062 | 0.051 | 0.036 |
| *Nycticebus pygmaeus* | x | 1.118 | 1.109 | 1.142 | 1.101 | 1.084 | 0.797 | 0.968 | 1.366 | 1.406 | 1.244 | 0.506 | 0.830 | 0.765 |
| N=2 | s.d. | 0.002 | 0.044 | 0.005 | 0.004 | 0.009 | 0.003 | 0.004 | 0.025 | 0.037 | 0.020 | 0.009 | 0.019 | 0.027 |
| *Daubentonia mad.* | x | 1.149 | 1.315 | 1.371 | 1.354 | 1.293 | 0.580 | 1.043 | 1.126 | 1.205 | 1.079 | 0.573 | 0.739 | 0.743 |
| N=9 | s.d. | 0.020 | 0.036 | 0.033 | 0.055 | 0.055 | 0.019 | 0.020 | 0.033 | 0.030 | 0.023 | 0.011 | 0.017 | 0.078 |
| *Microcebus murinus* | x | 1.250 | 1.353 | 1.388 | 1.339 | 1.178 | 0.734 | 0.935 | 1.116 | 1.256 | 0.996 | 0.539 | 0.761 | 0.695 |
| N=11 | s.d. | 0.055 | 0.086 | 0.039 | 0.043 | 0.037 | 0.023 | 0.022 | 0.026 | 0.040 | 0.030 | 0.053 | 0.023 | 0.025 |
| *Cheirogaleus medius* | x | 1.370 | 1.537 | 1.437 | 1.387 | 1.213 | 0.724 | 0.974 | 1.079 | 1.161 | 0.994 | 0.526 | 0.666 | 0.641 |
| N=6 | s.d. | 0.037 | 0.032 | 0.020 | 0.022 | 0.032 | 0.030 | 0.019 | 0.012 | 0.023 | 0.034 | 0.015 | 0.009 | 0.032 |
| *Cheirogaleus major* | x | 1.336 | 1.492 | 1.417 | 1.378 | 1.219 | 0.745 | 1.001 | 1.079 | 1.150 | 1.023 | 0.521 | 0.669 | 0.642 |
| N=6 | s.d. | 0.041 | 0.042 | 0.049 | 0.027 | 0.028 | 0.022 | 0.017 | 0.023 | 0.030 | 0.041 | 0.028 | 0.017 | 0.029 |
| *Lepilemur mustelinus* | x | 1.367 | 1.530 | 1.573 | 1.527 | 1.372 | 0.719 | 0.991 | 1.081 | 1.185 | 0.948 | 0.483 | 0.641 | 0.548 |
| N=5 | s.d. | 0.057 | 0.046 | 0.049 | 0.045 | 0.044 | 0.044 | 0.044 | 0.016 | 0.036 | 0.026 | 0.022 | 0.050 | 0.049 |
| *Avahi laniger* | x | 1.529 | 1.597 | 1.668 | 1.678 | 1.558 | 0.707 | 0.976 | 1.043 | 1.150 | 0.961 | 0.393 | 0.569 | 0.537 |
| N=9 | s.d. | 0.045 | 0.049 | 0.034 | 0.036 | 0.049 | 0.025 | 0.020 | 0.029 | 0.047 | 0.034 | 0.066 | 0.022 | 0.023 |
| *Propithecus verreauxi* | x | 1.442 | 1.497 | 1.531 | 1.569 | 1.479 | 0.759 | 0.965 | 1.029 | 1.129 | 1.001 | 0.476 | 0.574 | 0.566 |
| N=11 | s.d. | 0.031 | 0.042 | 0.044 | 0.050 | 0.045 | 0.018 | 0.022 | 0.029 | 0.022 | 0.018 | 0.045 | 0.037 | 0.016 |
| *Propithecus diadema* | x | 1.427 | 1.482 | 1.541 | 1.552 | 1.497 | 0.771 | 0.965 | 1.034 | 1.148 | 1.031 | 0.442 | 0.569 | 0.579 |
| N=6 | s.d. | 0.052 | 0.042 | 0.024 | 0.028 | 0.033 | 0.024 | 0.011 | 0.026 | 0.022 | 0.030 | 0.024 | 0.019 | 0.022 |
| *Indri indri* | x | 1.440 | 1.508 | 1.526 | 1.550 | 1.496 | 0.800 | 0.937 | 1.038 | 1.109 | 0.979 | 0.487 | 0.575 | 0.556 |
| N=7 | s.d. | 0.039 | 0.048 | 0.045 | 0.060 | 0.058 | 0.024 | 0.046 | 0.024 | 0.036 | 0.035 | 0.035 | 0.033 | 0.026 |
| *Eulemur macaco* | x | 1.390 | 1.560 | 1.574 | 1.581 | 1.460 | 0.737 | 0.952 | 1.047 | 1.142 | 0.975 | 0.461 | 0.595 | 0.567 |
| N=7 | s.d. | 0.036 | 0.025 | 0.018 | 0.018 | 0.018 | 0.011 | 0.030 | 0.017 | 0.018 | 0.022 | 0.026 | 0.019 | 0.026 |
| *Eulemur mongoz* | x | 1.355 | 1.514 | 1.538 | 1.531 | 1.441 | 0.730 | 0.952 | 1.053 | 1.140 | 0.986 | 0.469 | 0.632 | 0.591 |
| N=5 | s.d. | 0.033 | 0.042 | 0.035 | 0.040 | 0.022 | 0.015 | 0.011 | 0.020 | 0.012 | 0.034 | 0.016 | 0.015 | 0.013 |
| *Eulemur fulvus* | x | 1.370 | 1.549 | 1.582 | 1.579 | 1.472 | 0.730 | 0.935 | 1.049 | 1.133 | 0.970 | 0.475 | 0.610 | 0.565 |
| N=7 | s.d. | 0.035 | 0.038 | 0.049 | 0.041 | 0.022 | 0.013 | 0.022 | 0.010 | 0.027 | 0.019 | 0.029 | 0.016 | 0.017 |
| *Varecia variegata* | x | 1.256 | 1.562 | 1.524 | 1.488 | 1.388 | 0.681 | 0.979 | 1.062 | 1.131 | 1.002 | 0.500 | 0.649 | 0.624 |
| N=9 | s.d. | 0.017 | 0.023 | 0.022 | 0.020 | 0.032 | 0.012 | 0.018 | 0.022 | 0.023 | 0.025 | 0.017 | 0.026 | 0.013 |
| *Lemur catta* | x | 1.378 | 1.565 | 1.563 | 1.574 | 1.465 | 0.729 | 0.938 | 1.029 | 1.121 | 0.937 | 0.484 | 0.625 | 0.583 |
| N=11 | s.d. | 0.036 | 0.047 | 0.026 | 0.029 | 0.033 | 0.028 | 0.022 | 0.025 | 0.026 | 0.039 | 0.058 | 0.016 | 0.026 |
| *Hapalemur griseus* | x | 1.397 | 1.580 | 1.591 | 1.627 | 1.524 | 0.708 | 0.915 | 1.061 | 1.144 | 0.940 | 0.442 | 0.636 | 0.555 |
| N=12 | s.d. | 0.049 | 0.026 | 0.027 | 0.023 | 0.031 | 0.029 | 0.017 | 0.015 | 0.021 | 0.019 | 0.017 | 0.016 | 0.025 |
| **Taxon** | **value** | **pp2Vv** | **pp3Vv** | **pp4Vv** | pp5Vv | **ip2Vv** | **ip3Vv** | **ip5Vv** |  |  |  |  |  |  |
| *Notharctus* 143612 |  | 1.094 | 1.280 | 1.322 | 1.140 | 0.736 | 0.836 | 0.770 |  |  |  |  |  |  |
| *Darwinius masillae* |  | 1.173 | 1.347 | 1.503 | 1.312 | 0.582 | 0.756 | 0.730 |  |  |  |  |  |  |
| *Galago senegalensis* | x | 1.080 | 1.366 | 1.539 | 1.185 | 0.559 | 0.862 | 0.775 |  |  |  |  |  |  |
| N=12 | s.d. | 0.024 | 0.032 | 0.054 | 0.025 | 0.024 | 0.029 | 0.030 |  |  |  |  |  |  |
| *Galago moholi* | x | 1.055 | 1.345 | 1.517 | 1.159 | 0.574 | 0.905 | 0.773 |  |  |  |  |  |  |
| N=7 | s.d. | 0.019 | 0.022 | 0.034 | 0.022 | 0.026 | 0.052 | 0.025 |  |  |  |  |  |  |
| *Galagoides demidoff* | x | 1.082 | 1.362 | 1.627 | 1.240 | 0.508 | 0.868 | 0.770 |  |  |  |  |  |  |
| N=9 | s.d. | 0.037 | 0.034 | 0.028 | 0.039 | 0.036 | 0.053 | 0.070 |  |  |  |  |  |  |
| *Euoticus elegantulus* | x | 1.139 | 1.371 | 1.549 | 1.291 | 0.535 | 0.811 | 0.743 |  |  |  |  |  |  |
| N=9 | s.d. | 0.017 | 0.019 | 0.034 | 0.046 | 0.047 | 0.045 | 0.018 |  |  |  |  |  |  |
| *Galagoides alleni* | x | 1.050 | 1.360 | 1.591 | 1.202 | 0.531 | 0.864 | 0.800 |  |  |  |  |  |  |
| N=3 | s.d. | 0.033 | 0.041 | 0.076 | 0.033 | 0.015 | 0.034 | 0.027 |  |  |  |  |  |  |
| *Otolemur crassicaudatus* | x | 1.097 | 1.411 | 1.569 | 1.249 | 0.538 | 0.821 | 0.751 |  |  |  |  |  |  |
| N=9 | s.d. | 0.022 | 0.049 | 0.067 | 0.036 | 0.045 | 0.021 | 0.020 |  |  |  |  |  |  |
| *Otolemur garnettii* | x | 1.092 | 1.406 | 1.594 | 1.282 | 0.508 | 0.830 | 0.759 |  |  |  |  |  |  |
| N=7 | s.d. | 0.031 | 0.049 | 0.035 | 0.029 | 0.012 | 0.045 | 0.021 |  |  |  |  |  |  |
| *Arctocebus calabarensis* | x | 0.977 | 1.362 | 1.809 | 1.447 | 0.470 | 0.739 | 0.834 |  |  |  |  |  |  |
| N=11 | s.d. | 0.055 | 0.050 | 0.043 | 0.062 | 0.032 | 0.045 | 0.048 |  |  |  |  |  |  |
| *Perodicticus potto* | x | 0.987 | 1.425 | 1.683 | 1.434 | 0.452 | 0.811 | 0.825 |  |  |  |  |  |  |
| N=12 | s.d. | 0.064 | 0.065 | 0.087 | 0.056 | 0.092 | 0.081 | 0.037 |  |  |  |  |  |  |
| *Loris tardigradus* | x | 1.036 | 1.466 | 1.642 | 1.440 | 0.399 | 0.825 | 0.855 |  |  |  |  |  |  |
| N=10 | s.d. | 0.047 | 0.048 | 0.057 | 0.050 | 0.048 | 0.024 | 0.026 |  |  |  |  |  |  |
| *Nycticebus coucang* | x | 1.032 | 1.453 | 1.537 | 1.351 | 0.481 | 0.846 | 0.798 |  |  |  |  |  |  |
| N=16 | s.d. | 0.042 | 0.051 | 0.059 | 0.052 | 0.061 | 0.052 | 0.038 |  |  |  |  |  |  |
| *Nycticebus pygmaeus* | x | 1.010 | 1.425 | 1.467 | 1.298 | 0.528 | 0.866 | 0.799 |  |  |  |  |  |  |
| N=2 | s.d. | 0.001 | 0.021 | 0.034 | 0.017 | 0.007 | 0.023 | 0.031 |  |  |  |  |  |  |
| *Daubentonia mad.* | x | 1.159 | 1.252 | 1.339 | 1.199 | 0.637 | 0.822 | 0.825 |  |  |  |  |  |  |
| N=9 | s.d. | 0.024 | 0.042 | 0.035 | 0.031 | 0.011 | 0.024 | 0.078 |  |  |  |  |  |  |
| *Microcebus murinus* | x | 1.078 | 1.287 | 1.447 | 1.148 | 0.621 | 0.877 | 0.801 |  |  |  |  |  |  |
| N=11 | s.d. | 0.032 | 0.038 | 0.053 | 0.033 | 0.057 | 0.022 | 0.024 |  |  |  |  |  |  |
| *Cheirogaleus medius* | x | 1.174 | 1.300 | 1.398 | 1.197 | 0.633 | 0.802 | 0.772 |  |  |  |  |  |  |
| N=6 | s.d. | 0.016 | 0.015 | 0.029 | 0.044 | 0.017 | 0.011 | 0.037 |  |  |  |  |  |  |
| *Cheirogaleus major* | x | 1.198 | 1.292 | 1.377 | 1.224 | 0.624 | 0.801 | 0.769 |  |  |  |  |  |  |
| N=6 | s.d. | 0.021 | 0.017 | 0.029 | 0.032 | 0.034 | 0.023 | 0.026 |  |  |  |  |  |  |
| *Lepilemur mustelinus* | x | 1.245 | 1.358 | 1.488 | 1.191 | 0.607 | 0.805 | 0.689 |  |  |  |  |  |  |
| N=5 | s.d. | 0.051 | 0.029 | 0.051 | 0.043 | 0.026 | 0.052 | 0.058 |  |  |  |  |  |  |
| *Avahi laniger* | x | 1.302 | 1.392 | 1.534 | 1.282 | 0.524 | 0.759 | 0.717 |  |  |  |  |  |  |
| N=9 | s.d. | 0.033 | 0.048 | 0.071 | 0.048 | 0.084 | 0.030 | 0.036 |  |  |  |  |  |  |
| *Propithecus verreauxi* | x | 1.240 | 1.324 | 1.452 | 1.287 | 0.611 | 0.737 | 0.728 |  |  |  |  |  |  |
| N=11 | s.d. | 0.036 | 0.052 | 0.035 | 0.029 | 0.052 | 0.037 | 0.020 |  |  |  |  |  |  |
| *Propithecus diadema* | x | 1.241 | 1.330 | 1.476 | 1.326 | 0.569 | 0.731 | 0.745 |  |  |  |  |  |  |
| N=6 | s.d. | 0.027 | 0.021 | 0.024 | 0.040 | 0.029 | 0.016 | 0.019 |  |  |  |  |  |  |
| *Indri indri* | x | 1.214 | 1.345 | 1.437 | 1.269 | 0.631 | 0.744 | 0.720 |  |  |  |  |  |  |
| N=7 | s.d. | 0.058 | 0.041 | 0.055 | 0.054 | 0.038 | 0.031 | 0.020 |  |  |  |  |  |  |
| *Eulemur macaco* | x | 1.224 | 1.346 | 1.468 | 1.254 | 0.593 | 0.765 | 0.730 |  |  |  |  |  |  |
| N=7 | s.d. | 0.034 | 0.028 | 0.030 | 0.029 | 0.030 | 0.023 | 0.036 |  |  |  |  |  |  |
| *Eulemur mongoz* | x | 1.201 | 1.328 | 1.438 | 1.243 | 0.591 | 0.797 | 0.745 |  |  |  |  |  |  |
| N=5 | s.d. | 0.012 | 0.017 | 0.010 | 0.030 | 0.021 | 0.016 | 0.018 |  |  |  |  |  |  |
| *Eulemur fulvus* | x | 1.198 | 1.344 | 1.452 | 1.244 | 0.608 | 0.783 | 0.724 |  |  |  |  |  |  |
| N=7 | s.d. | 0.022 | 0.014 | 0.037 | 0.026 | 0.035 | 0.019 | 0.024 |  |  |  |  |  |  |
| *Varecia variegata* | x | 1.202 | 1.304 | 1.389 | 1.230 | 0.613 | 0.797 | 0.767 |  |  |  |  |  |  |
| N=9 | s.d. | 0.025 | 0.024 | 0.021 | 0.031 | 0.020 | 0.031 | 0.015 |  |  |  |  |  |  |
| *Lemur catta* | x | 1.201 | 1.318 | 1.436 | 1.201 | 0.620 | 0.800 | 0.746 |  |  |  |  |  |  |
| N=11 | s.d. | 0.029 | 0.038 | 0.044 | 0.055 | 0.071 | 0.020 | 0.035 |  |  |  |  |  |  |
| *Hapalemur griseus* | x | 1.186 | 1.376 | 1.483 | 1.218 | 0.573 | 0.825 | 0.719 |  |  |  |  |  |  |
| N=12 | s.d. | 0.019 | 0.022 | 0.031 | 0.027 | 0.018 | 0.017 | 0.029 |  |  |  |  |  |  |

C. Means and standard deviations of haplorhine species used in the analyses.

| **Taxon** | **value** | **Mt1V** | **Mt2V** | **Mt3V** | **Mt4V** | **Mt5V** | **pp1V** | **pp2V** | **pp3V** | **pp4V** | **pp5V** | **ip2V** | **ip3V** | **ip5V** |
| --- | --- | --- | --- | --- | --- | --- | --- | --- | --- | --- | --- | --- | --- | --- |
| *Tarsius spectrum* | x | 1.327 | 1.228 | 1.400 | 1.374 | 1.196 | 0.773 | 0.951 | 1.117 | 1.386 | 1.090 | 0.515 | 0.564 | 0.744 |
| N=2 | s.d. | 0.037 | 0.025 | 0.056 | 0.028 | 0.030 | 0.029 | 0.007 | 0.014 | 0.041 | 0.001 | 0.037 | 0.019 | 0.011 |
| *Tarsius syrichta* | x | 1.310 | 1.191 | 1.426 | 1.385 | 1.186 | 0.820 | 0.924 | 1.124 | 1.330 | 1.069 | 0.504 | 0.654 | 0.690 |
| N=9 | s.d. | 0.043 | 0.046 | 0.038 | 0.031 | 0.031 | 0.018 | 0.017 | 0.039 | 0.040 | 0.055 | 0.035 | 0.025 | 0.026 |
| *Tarsius bancanus* | x | 1.310 | 1.224 | 1.425 | 1.386 | 1.205 | 0.804 | 0.947 | 1.134 | 1.368 | 1.109 | 0.500 | 0.565 | 0.725 |
| N=10 | s.d. | 0.047 | 0.051 | 0.046 | 0.041 | 0.045 | 0.017 | 0.052 | 0.038 | 0.074 | 0.064 | 0.069 | 0.031 | 0.050 |
| *Saguinus midas* | x | 1.003 | 1.762 | 1.911 | 2.034 | 1.898 | 0.590 | 0.924 | 0.995 | 0.991 | 0.874 | 0.503 | 0.617 | 0.528 |
| N=6 | s.d. | 0.022 | 0.060 | 0.043 | 0.036 | 0.075 | 0.021 | 0.008 | 0.011 | 0.016 | 0.020 | 0.015 | 0.017 | 0.024 |
| *Saguinus oedipus* | x | 0.950 | 1.693 | 1.923 | 2.059 | 1.931 | 0.613 | 0.957 | 1.027 | 1.032 | 0.884 | 0.491 | 0.602 | 0.505 |
| N=5 | s.d. | 0.068 | 0.044 | 0.066 | 0.081 | 0.043 | 0.022 | 0.033 | 0.026 | 0.035 | 0.030 | 0.025 | 0.026 | 0.016 |
| *Callithrix jacchus* | x | 0.964 | 1.770 | 1.937 | 2.055 | 1.936 | 0.554 | 0.891 | 0.996 | 0.971 | 0.865 | 0.518 | 0.621 | 0.576 |
| N=5 | s.d. | 0.040 | 0.043 | 0.058 | 0.066 | 0.066 | 0.028 | 0.019 | 0.008 | 0.005 | 0.010 | 0.027 | 0.021 | 0.012 |
| *Leontopithecus rosalia* | x | 0.980 | 1.795 | 1.982 | 2.105 | 1.991 | 0.526 | 0.866 | 0.978 | 0.987 | 0.796 | 0.532 | 0.635 | 0.580 |
| N=3 | s.d. | 0.027 | 0.006 | 0.012 | 0.016 | 0.013 | 0.011 | 0.021 | 0.018 | 0.006 | 0.011 | 0.004 | 0.012 | 0.025 |
| *Aotus sp.* | x | 1.041 | 1.640 | 1.651 | 1.778 | 1.696 | 0.646 | 0.949 | 1.010 | 1.018 | 0.855 | 0.605 | 0.680 | 0.531 |
| N=2 | s.d. | 0.003 | 0.021 | 0.007 | 0.022 | 0.035 | 0.017 | 0.014 | 0.015 | 0.056 | 0.001 | 0.009 | 0.015 | 0.036 |
| *Alouatta sp*. (N=1) |  | 1.103 | 1.550 | 1.548 | 1.518 | 1.487 | 0.645 | 1.040 | 1.051 | 1.083 | 0.897 | 0.592 | 0.676 | 0.612 |
| *Saimiri sp.* | x | 1.073 | 1.664 | 1.794 | 1.904 | 1.793 | 0.606 | 0.891 | 0.985 | 0.987 | 0.848 | 0.538 | 0.716 | 0.538 |
| N=5 | s.d. | 0.053 | 0.098 | 0.057 | 0.030 | 0.051 | 0.024 | 0.019 | 0.023 | 0.042 | 0.027 | 0.032 | 0.015 | 0.045 |
| *Cebus sp.* | x | 1.155 | 1.675 | 1.755 | 1.766 | 1.638 | 0.643 | 0.988 | 1.003 | 0.998 | 0.845 | 0.627 | 0.642 | 0.477 |
| N=2 | s.d. | 0.112 | 0.122 | 0.023 | 0.002 | 0.063 | 0.007 | 0.033 | 0.047 | 0.043 | 0.113 | 0.055 | 0.024 | 0.000 |
| *Cacajao rubicundus* (N=1) |  | 1.004 | 1.545 | 1.618 | 1.623 | 1.645 | 0.641 | 0.927 | 0.988 | 1.039 | 0.935 | 0.599 | 0.715 | 0.611 |
| **Taxon** | **value** | **pp2Vv** | **pp3Vv** | **pp4Vv** | **pp5Vv** | **ip2Vv** | **ip3Vv** | **ip5Vv** |  |  |  |  |  |  |
| *Tarsius spectrum* | x | 1.107 | 1.300 | 1.613 | 1.268 | 0.599 | 0.656 | 0.865 |  |  |  |  |  |  |
| N=2 | s.d. | 0.014 | 0.024 | 0.057 | 0.008 | 0.039 | 0.019 | 0.018 |  |  |  |  |  |  |
| *Tarsius syrichta* | x | 1.081 | 1.314 | 1.556 | 1.250 | 0.589 | 0.765 | 0.807 |  |  |  |  |  |  |
| N=9 | s.d. | 0.017 | 0.055 | 0.032 | 0.051 | 0.038 | 0.026 | 0.035 |  |  |  |  |  |  |
| *Tarsius bancanus* | x | 1.111 | 1.330 | 1.604 | 1.300 | 0.586 | 0.662 | 0.850 |  |  |  |  |  |  |
| N=10 | s.d. | 0.076 | 0.055 | 0.089 | 0.079 | 0.075 | 0.028 | 0.051 |  |  |  |  |  |  |
| *Saguinus midas* | x | 1.236 | 1.331 | 1.326 | 1.169 | 0.673 | 0.826 | 0.707 |  |  |  |  |  |  |
| N=6 | s.d. | 0.009 | 0.007 | 0.016 | 0.022 | 0.018 | 0.020 | 0.034 |  |  |  |  |  |  |
| *Saguinus oedipus* | x | 1.276 | 1.369 | 1.375 | 1.179 | 0.656 | 0.802 | 0.673 |  |  |  |  |  |  |
| N=5 | s.d. | 0.028 | 0.021 | 0.027 | 0.031 | 0.040 | 0.028 | 0.026 |  |  |  |  |  |  |
| *Callithrix jacchus* | x | 1.183 | 1.322 | 1.289 | 1.149 | 0.687 | 0.824 | 0.764 |  |  |  |  |  |  |
| N=5 | s.d. | 0.026 | 0.011 | 0.021 | 0.007 | 0.027 | 0.015 | 0.017 |  |  |  |  |  |  |
| *Leontopithecus rosalia* | x | 1.159 | 1.309 | 1.320 | 1.065 | 0.712 | 0.850 | 0.775 |  |  |  |  |  |  |
| N=3 | s.d. | 0.028 | 0.026 | 0.010 | 0.015 | 0.005 | 0.014 | 0.033 |  |  |  |  |  |  |
| *Aotus sp.* | x | 1.210 | 1.288 | 1.299 | 1.090 | 0.772 | 0.868 | 0.678 |  |  |  |  |  |  |
| N=2 | s.d. | 0.014 | 0.015 | 0.067 | 0.005 | 0.015 | 0.016 | 0.048 |  |  |  |  |  |  |
| *Alouatta sp*. (N=1) |  | 1.261 | 1.275 | 1.313 | 1.087 | 0.717 | 0.820 | 0.741 |  |  |  |  |  |  |
| *Saimiri sp.* | x | 1.167 | 1.290 | 1.293 | 1.110 | 0.705 | 0.938 | 0.704 |  |  |  |  |  |  |
| N=5 | s.d. | 0.014 | 0.015 | 0.054 | 0.020 | 0.044 | 0.016 | 0.054 |  |  |  |  |  |  |
| *Cebus sp.* | x | 1.284 | 1.304 | 1.298 | 1.097 | 0.817 | 0.834 | 0.620 |  |  |  |  |  |  |
| N=2 | s.d. | 0.005 | 0.023 | 0.018 | 0.115 | 0.096 | 0.006 | 0.019 |  |  |  |  |  |  |
| *Cacajao rubicundus* (N=1) |  | 1.142 | 1.216 | 1.280 | 1.151 | 0.737 | 0.880 | 0.753 |  |  |  |  |  |  |

*Abbreviations: Mt, metatarsal; proximal phalanx; ip, intermediate phalanx; Gm, geometric mean.

**Group codes used in discriminant function analyses.

**Table Legend**

Means (x) and standard deviations (s.d.) of shape variables used in two discriminant function analyses. Variables ending in ‘V’ were used in the first analysis; those ending in ‘Vv’ were used in the second (See Materials and Methods, and Results). Table A, Means and standard deviations of groups discriminated among, along with fossil values; Table B, Means and standard deviations of strepsirhine species used in the analyses, along with fossil values; Table C, Means and standard deviations of haplorhine species used in the analyses.
