# Supplementary material for: Evidence for a Grooming Claw in a North American Adapiform Primate: Implications for Anthropoid Origins
Source: PLoS One. 2012 Jan 10;7(1):e29135. doi: 10.1371/journal.pone.0029135 (PMC3254620; doi:10.1371/journal.pone.0029135)
Supplement: Table S4 — Standardized canonical discriminant function coefficients. Coefficients from two discriminant function analyses. A, Discriminant function analysis of metatarsal and phalanx shape variables; B, Discriminant function analysis of phalanx shape variables only. See Table S3 for variable means and standard deviations. (DOC) [file pone.0029135.s007.doc]

**Table S4. Standardized canonical discriminant function coefficients.**

A. Discriminant function analysis of metatarsal and phalanx shape variables.

|  | **Function # (eigenvalue/percent of variance explained)** | | | | | | | |
| --- | --- | --- | --- | --- | --- | --- | --- | --- |
|  | **1 (28.9/69.8)** | **2 (6.5/15.7)** | **3 (2.1/5.0)** | **4 (1.8/4.4)** | **5 (1.2/2.8)** | **6 (0.7/1.7)** | **7 (0.2/0.5)** | **8 (0.1/0.2)** |
| **Mt1V*** | 0.567 | 0.875 | 0.889 | 0.571 | 0.072 | 0.536 | 1.694 | 0.437 |
| **Mt2V** | -0.054 | 1.218 | 0.270 | -0.237 | 0.582 | -0.767 | 0.896 | 0.365 |
| **Mt3V** | -0.037 | 0.336 | -0.723 | -0.067 | -0.623 | 0.348 | 0.818 | 0.801 |
| **Mt4V** | 0.074 | 0.796 | 0.078 | 0.977 | -0.249 | -0.885 | 1.193 | -0.095 |
| **Mt5V** | -0.438 | 1.511 | 0.721 | -0.342 | 0.342 | 1.387 | 0.308 | -0.147 |
| **pp1V** | 0.386 | 1.493 | 0.108 | -0.482 | -0.515 | -0.081 | 1.684 | -0.203 |
| **pp2V** | -0.420 | 0.485 | 0.092 | 0.011 | 0.258 | 0.700 | 1.411 | 0.384 |
| **pp3V** | 0.850 | 1.041 | 0.239 | -0.160 | -0.347 | -0.247 | 0.652 | 0.261 |
| **pp4V** | 0.032 | 0.478 | -0.628 | -0.103 | 0.237 | 0.428 | 0.141 | 0.643 |
| **pp5V** | 0.369 | 1.105 | 0.734 | 0.824 | 0.045 | -0.193 | 0.826 | 0.392 |
| **ip2V** | 0.055 | 2.010 | -0.023 | 0.560 | 0.096 | 0.278 | 2.061 | 0.611 |
| **ip3V** | -0.033 | 1.578 | 0.253 | -0.259 | 0.540 | 0.306 | 1.621 | 0.271 |
| **ip5V** | 0.305 | 1.068 | -0.070 | 0.539 | 0.219 | 0.177 | 1.210 | -0.341 |
|  |  | | | | | | | |
|  |  |  |  |  |  |  |  |  |
|  |  |  |  |  |  |  |  |  |
|  |  |  |  |  |  |  |  |  |
|  |  |  |  |  |  |  |  |  |
|  |  |  |  |  |  |  |  |  |
|  |  |  |  |  |  |  |  |  |
|  |  |  |  |  |  |  |  |  |
|  |  |  |  |  |  |  |  |  |
|  |  |  |  |  |  |  |  |  |
|  |  |  |  |  |  |  |  |  |

B. Discriminant function analysis of phalanx shape variables only.

| **Function # (eigenvalue/percent variance explained)** | | | | | | | |
| --- | --- | --- | --- | --- | --- | --- | --- |
|  | **1 (8.4/69.9)** | **2 (1.6/13.3)** | **3 (1.0/8.4)** | **4 (0.7/5.6)** | **5 (0.3/2.8)** | **6 (0.0/0.2)** | **7(0.0/0.0)** |
| **pp2Vv** | -0.869 | 1.259 | 0.137 | 0.125 | 0.473 | 1.279 | 0.427 |
| **pp3Vv** | 0.571 | 1.356 | 0.614 | 0.076 | 1.127 | 0.058 | 0.708 |
| **pp4Vv** | 0.127 | 0.628 | 0.466 | 0.517 | 1.212 | 1.549 | -0.067 |
| **pp5Vv** | 0.533 | 2.173 | -0.066 | -0.138 | 0.148 | 0.677 | -0.350 |
| **ip2Vv** | 0.080 | 3.466 | 0.922 | 0.978 | 1.954 | 1.535 | -0.058 |
| **ip3Vv** | 0.076 | 2.011 | 1.145 | 0.157 | 0.363 | 1.540 | 0.041 |
| **ip5Vv** | 0.183 | 1.837 | 0.328 | 1.015 | 0.552 | 1.160 | 0.734 |

*Abbreviations: Mt, metatarsal; proximal phalanx; ip, intermediate phalanx

**Table Legend**

Coefficients from two discriminant function analyses. A, Discriminant function analysis of metatarsal and phalanx shape variables; B, Discriminant function analysis of phalanx shape variables only. See Table S3 for variable means and standard deviations.
