# Supplementary material for: Evidence for a Grooming Claw in a North American Adapiform Primate: Implications for Anthropoid Origins
Source: PLoS One. 2012 Jan 10;7(1):e29135. doi: 10.1371/journal.pone.0029135 (PMC3254620; doi:10.1371/journal.pone.0029135)
Supplement: Table S5 — Mandibular depth of fossil and extant primates. Measurements are reported in millimeters. See Appendix S1 section 2 for details. (DOC) [file pone.0029135.s008.doc]

Table S5. Mandibular depth of fossil and extant primates.

| **Anthropoidea** | | | | |
| --- | --- | --- | --- | --- |
| **Taxon** | **Mandibular Depth** | **M_2_ Length** | **MDI*** | ***n*** |
| *Ateles belzebuth* | 19.2 | 5.1 | 3.8 | 6 |
| *Alouatta belzebul* | 23.0 | 7.6 | 3.0 | 6 |
| *Cebus paella* | 15.2 | 4.5 | 3.4 | 6 |
| *Saimiri boliviensis* | 7.3 | 2.4 | 3.0 | 6 |
| *Callicebus torquatus* | 14.8 | 3.6 | 4.1 | 6 |
| *Callicebus cupreus* | 14.4 | 3.5 | 4.2 | 6 |
| *Pithecia monachus* | 18.1 | 3.9 | 4.7 | 6 |
| *Chiropotes albinasus* | 20.9 | 3.8 | 5.5 | 6 |
| *Aotus lemurinus* | 10.2 | 3.2 | 3.2 | 6 |
| *Aotus nigriceps* | 10.9 | 3.2 | 3.4 | 6 |
| *Callithrix argentata* | 6.5 | 2.1 | 3.1 | 6 |
| *Saguinus fuscicollis* | 6.3 | 2.1 | 2.9 | 6 |
| **Mean** | **13.9** | **3.7** | **3.7** | **72** |
| **Tarsioidea** | | | | |
| *Tarsius spectrum* | 3.3 | 2.3 | 1.5 | 6 |
| *Tarsius syrichta* | 3.2 | 2.7 | 1.2 | 6 |
| **Mean** | **3.2** | **2.5** | **1.3** | **12** |
| **Lorisoidea** | | | | |
| *Otolemur crassicaudatus* | 6.6 | 3.7 | 1.8 | 10 |
| *Galago senegalensis* | 3.2 | 2.2 | 1.5 | 12 |
| *Nycticebus coucang* | 7.2 | 3.2 | 2.2 | 6 |
| *Perodicticus potto* | 7.6 | 3.3 | 2.3 | 7 |
| **Mean** | **6.2** | **3.1** | **1.9** | **35** |
| **Lemuroidea** | | | | |
| *Eulemur fulvus rufus* | 8.0 | 5.9 | 1.4 | 9 |
| *Propithecus verreauxi* | 12.4 | 6.4 | 1.9 | 11 |
| *Cheirogeleus major* | 4.7 | 3.0 | 1.6 | 3 |
| *Microcebus murinus* | 2.6 | 1.5 | 1.8 | 9 |
| **Mean** | **6.9** | **4.2** | **1.7** | **32** |
| **Fossil Species** | | | | |
| *Darwinius masillae* | 7.3 | 4.2 | 1.7 | 1 |
| *Notharctus tenebrosus* | 10.1 | 5.9 | 1.7 | 7 |
| *Catopithecus browni* | 6.9 | 3.0 | 2.3 | 8 |

*MDI, Mandibular depth index; mandibular depth divided by M_2_ length.

**Table Legend**

Measurements are reported in millimeters. See Appendix S1 section 1 for details.
