# Supplementary material for: Evidence for a Grooming Claw in a North American Adapiform Primate: Implications for Anthropoid Origins
Source: PLoS One. 2012 Jan 10;7(1):e29135. doi: 10.1371/journal.pone.0029135 (PMC3254620; doi:10.1371/journal.pone.0029135)
Supplement: Table S6 — Gingerich et al. (2010) character matrix with our corrections and updates. Missing data, ‘?’ are highlighted in yellow. See Appendix S1, sections 2–5 for details on corrections, additional characters, and additional fossil taxa; and section 16 for the text of the corresponding nexus file. (DOC) [file pone.0029135.s009.doc]

**Table S6. Gingerich et al. (2010) character matrix with our corrections and additions.**

|  | **0** | **0** | **0** | **0** | **0** | **0** | **0** | **0** | **0** | **1** | **1** | **1** | **1** | **1** | **1** | **1** | **1** | **1** | **1** | **2** | **2** | **2** | **2** | **2** | **2** | **2** | **2** | **2** | **2** | **3** | **3** | **3** | **3** | **3** | **3** | **3** | **3** | **3** | **3** |
| --- | --- | --- | --- | --- | --- | --- | --- | --- | --- | --- | --- | --- | --- | --- | --- | --- | --- | --- | --- | --- | --- | --- | --- | --- | --- | --- | --- | --- | --- | --- | --- | --- | --- | --- | --- | --- | --- | --- | --- |
| **Taxon** | **1** | **2** | **3** | **4** | **5** | **6** | **7** | **8** | **9** | **0** | **1** | **2** | **3** | **4** | **5** | **6** | **7** | **8** | **9** | **0** | **1** | **2** | **3** | **4** | **5** | **6** | **7** | **8** | **9** | **0** | **1** | **2** | **3** | **4** | **5** | **6** | **7** | **8** | **9** |
| TUPAIOIDEA | 0 | 0 | 0 | 0 | 0 | 0 | 0 | 0 | 0 | 0 | 0 | 0 | 0 | 0 | 0 | 0 | 1 | 0 | 0 | 0 | 1 | 1 | 1 | 1 | 0 | 0 | 0 | 0 | 0 | 0 | 0 | 0 | 0 | 0 | 0 | 0 | 0 | & | * |
| LEMUROIDEA | 0 | 0 | 0 | 0 | 0 | 0 | 0 | 0 | 0 | 0 | 0 | 0 | * | 0 | 0 | 0 | 1 | 0 | 2 | 0 | 0 | 0 | 0 | 1 | 1 | 0 | 0 | 0 | 0 | 0 | 1 | 1 | 1 | 1 | 1 | 1 | 2 | # | 2 |
| LORISOIDEA | 0 | 0 | 0 | 0 | 0 | 0 | 0 | 0 | 0 | 0 | 0 | 1 | 0 | 0 | 0 | 0 | 1 | 0 | 2 | 0 | 0 | 0 | 1 | 1 | 1 | 0 | 0 | 0 | 0 | 0 | 1 | * | 1 | 1 | 1 | 1 | 2 | 0 | 2 |
| TARSIOIDEA | 1 | 0 | 1 | 1 | 0 | 0 | 1 | 1 | 0 | 0 | 1 | 2 | * | 0 | 1 | 0 | 0 | 0 | 0 | 1 | 1 | 0 | 1 | 1 | 1 | 0 | 0 | 1 | 0 | 1 | 0 | 0 | 1 | 1 | 1 | 0 | 1 | 0 | 1 |
| CEBOIDEA | 1 | 0 | 1 | 1 | 1 | 2 | 1 | 1 | 1 | 1 | 2 | 1 | 2 | 1 | 1 | 1 | 1 | 1 | + | 1 | 1 | 1 | 1 | * | + | 1 | 1 | 1 | 0 | 1 | 0 | 0 | 0 | 0 | 0 | 0 | 1 | 2 | 1 |
| CERCOPITH. | 1 | 1 | 1 | 1 | 1 | 2 | 1 | 1 | 1 | 1 | 2 | 2 | 2 | 1 | 1 | 1 | 1 | 1 | 2 | 1 | 1 | 1 | 1 | 0 | 2 | 1 | 1 | 1 | 1 | 1 | 0 | 0 | 0 | 0 | * | 0 | 1 | 2 | 1 |
| HOMINOIDEA | 1 | 1 | 1 | 1 | 1 | 2 | 1 | 1 | 1 | 1 | 2 | 2 | 2 | 1 | 1 | 1 | 1 | 1 | 2 | 1 | 1 | 1 | 1 | 0 | 2 | 1 | 1 | 1 | 1 | 1 | 0 | 0 | 0 | 0 | 0 | 0 | 1 | 2 | 1 |
| *Darwinius* | ? | ? | ? | ? | 0 | ? | ? | 1 | 0 | 0 | 0 | 0 | 1 | 1 | 1 | ? | 1 | ? | 2 | ? | ? | 0 | 1 | 1 | ? | ? | ? | ? | ? | ? | ? | ? | ? | ? | 1 | 1 | 2 | 0 | ? |
| *Notharctus* | ? | ? | ? | ? | 0 | 0 | 0 | 0 | 0 | 0 | 0 | 0 | + | 1 | 1 | 1 | 1 | 0 | 1 | 0 | 0 | 0 | 1 | 1 | 1 | ? | ? | ? | ? | ? | 1 | 1 | 1 | 1 | 1 | 1 | 2 | 2 | 2 |
| *Catopithecus* | ? | ? | ? | ? | 0 | 1 | 1 | 1 | 0 | 1 | 2 | 1 | 1 | 1 | 1 | 1 | 1 | 1 | 1 | ? | 1 | ? | ? | ? | ? | ? | ? | ? | ? | ? | 0 | 0 | 0 | 0 | 0 | ? | 1 | 2 | ? |

Polymorphism symbol definitions: *, 0/1; +, 1/2; &, 0/2; #, 0/1/2

**Table Legend**

Missing data, ‘?’ are highlighted in yellow. See Appendix S1 sections 1-3 for details on corrections, additional characters, and additional fossil taxa; and section 6 for the text of the corresponding nexus file.
