# Supplementary material for: Evidence for a Grooming Claw in a North American Adapiform Primate: Implications for Anthropoid Origins
Source: PLoS One. 2012 Jan 10;7(1):e29135. doi: 10.1371/journal.pone.0029135 (PMC3254620; doi:10.1371/journal.pone.0029135)
Supplement: Appendix S1 — Supplementary documentation of phylogenetic analyses. (DOC) [file pone.0029135.s010.doc]

**Supplementary documentation of phylogenetic analysis.**

In this supplementary document we provide additional iterations of cladistic analyses; and detailed explanations for our modifications to the character matrix of Gingerich et al. [S1] including coding changes and character additions. We begin by by presenting additional iterations of analysis of Gingerich et al.’s [S1] original matrix with additional fossil taxa added (Section 1). Next we explain the corrections we made to codings of the original matrix (Section 2). We then present results of adding only *Notharctus* to corrected character matrix (Section 3). We follow with explanations of our character additions (Section 4). Next, we give codings used for newly added fossil taxa (Section 5). Finally, we provide the text of the three nexus files we analyzed (Sections 6-18).

**Section 1. Adding fossil taxa to the original Gingerich et al. [S1] matrix**

*Results* -- In these analyses we did *not* correct or modify codings in Gingerich et al.’s [S1] original matrix before adding codings for additional taxa. First, we added codings for *Notharctus* only, and based on the comparative morphological results described in the main text, ran the analysis in two different ways. In one analysis we coded *Notharctus* as having a grooming claw, and in a second, we coded it as lacking one due to its unusually wide apical tuft. If *Notharctus* is scored as having a grooming claw, as justified by its overall closest resemblance to such bones (Fig. 10) and a distinctly inclined, tapering shaft and a restricted volar process (Figs. 11-12, 14), four most parsimonious trees result. *Darwinius* is always reconstructed as a stem-anthropoid, but *Notharctus* is reconstructed as a stem-haplorhine (the two adapiforms are separated by Tarsioidea) in three of the four trees (Fig. S1A; TL = 41, CI = 0.7561, HI = 0.2439, RI = 0.8529, RC = 0.6449). On the other hand, if the wide-apical tuft on the dp2 is acknowledged as the criterion for the coding (Fig. 13), and *Notharctus* is coded as lacking a grooming claw, the result is a single most-parsimonious tree in which both *Notharctus* and *Darwinius* are stem-anthropoids (Fig. S1B; TL = 41, CI = 0.7561, HI = 0.2439, RI = 0.8529, RC = 0.6449).

Next, we added *Catopithecus* and ran two more analyses. Again, one with *Notharctus* coded as having a grooming claw and one without. The first analysis, in which *Notharctus* is coded as having a grooming claw, results in three most parsimonious trees. In all of these trees, *Darwinius* is a stem-anthropoid, *Catopithecus* is the sister taxon to that group, and *Notharctus* is a stem-haplorhine (again separated from the other groups by Tarsioidea; Fig. S1C; TL = 42, CI = 0.7381, HI = 0.2619, RI = 0.8493, RC = 0.6269). Coding *Notharctus* as lacking a grooming claw complicates the picture (Fig S1D), resulting in seven most-parsimonious trees. The main volatility in the tree comes now from *Darwinius* occupying three different positions, either a stem-haplorhine position, next to *Notharctus*, a stem-anthropoid position distal to *Catopithecus*, and a stem-anthropoid position proximal to *Catopithecus* (TL = 43, CI = 0.7209, HI = 0.2791, RI = 0.8356, RC = 0.6204; see sections 6-9 for nexus files).

*Discussion*.- The results of reanalyzing Gingerich et al’s [S1] original matrix after adding codings of *Notharctus*, support the adapiform-anthropoid hypothesis fully, only if *Notharctus* is coded as lacking a grooming claw (Fig. S1B). This is not consistent with our interpretation of the new morphology, as discussed in the main text. Coding *Notharctus* as lacking a grooming claw results in a polyphyletic adapiforms separated by Tarsioidea, which we see as highly unlikely. Adding *Catopithecus* to the original matrix creates an even more untenable result in which adapiforms and anthropoids are polyphyletic, due to *Notharctus* taking a stem-haplorhine position in all resulting trees, and *Catopithecus* being separated from other anthropoids by *Darwinius* in six out of seven most parsimonious trees resulting from the two versions of this analysis (Fig. S1C-D). Given that *Darwinius* lacks key anthropoid characteristics that *Catopithecus* exhibits (like a postorbital septum), we suspect the original matrix of Gingerich et al. [S1] is insufficient for resolving the phylogenetic position of *Darwinius*.

**Section 2. Character Corrections.**

**Corrections implemented in the file: original matrix corrected.nex (see Section 8 below)**

**Character 6: Olfactory bulb size**

After consulting the literature, it is evident that some early anthropoid taxa possess olfactory bulbs which are intermediate in size compared to extant prosimian and extant anthropoid taxa (e.g., [S2-3]). Therefore, olfactory bulb size is best recognized as an ordered three-state character; relatively small = 0; intermediate = 1; relatively large = 2. *Catopithecus*, an early anthropoid taxon included in some of the phylogenetic analyses here, displays intermediate-sized olfactory bulbs [S3].

**Character 9: Mandibular corpus depth**

We changed the coding for *Darwinius* from “1” to “0” based on results of the analysis presented below.

A large sample of extant and fossil prosimian and anthropoid taxa was studied at the American Museum of Natural History (AMNH), the Field Museum (FMNH), Duke University Lemur Center Division of Fossil Primates (DLC), and the Stony Brook University Museum of Comparative Anatomy (SBU). Original specimens were measured wherever possible. When original specimens were not available, measurements were taken from casts, published measurements, or published photographs. Measurements were taken on 22 extant prosimian and platyrrhine species and 48 fossil prosimian and anthropoid taxa (Table S5). Platyrrhine primates were used as the extant anthropoid group in this study for two reasons. First, platyrrhine primates represent the most primitive extant anthropoid group and are, therefore, more likely to retain the ancestral condition exhibited by stem anthropoids than are catarrhine primates. Second, compared with catarrhines, the body size range of platyrrhines is relatively small and closer to the range exhibited by extant and fossil prosimian and stem anthropoid taxa. In addition, there is little sexual dimorphism exhibited among platyrrhine taxa. These factors help to naturally control for the confounding effects of allometry while investigating mandibular depth over a wide range of prosimian and anthropoid taxa.

Two measurements were taken for each specimen: mandibular depth under M_2_ (or under M_1_ where depth under the M_2_ was unavailable) and the maximum mesiodistal diameter of M_2_. All mandibular and dental measurements were taken with digital calipers and recorded to the nearest 0.1mm.

In order to compare taxa of different sizes, a size-adjusted index was created. Because molar tooth length is highly correlated with body size (e.g., [S4-6]), the mandibular depth measurement was divided by M_2_ length, creating a size-adjusted mandibular depth index (MDI) for each specimen. Using M_2_ length as a size-adjustment has the distinct advantage of being easily applied to the fossil record, most of which is comprised of teeth. In addition, because previous studies on mandibular depth have also used M_2_ length as a relative size measure (e.g., [S7-9], the results of our study are directly comparable to previous ones.

The MDI’s were analyzed by creating box-and-whisker plots of extant anthropoids, strepsirhines, tarsiers, and fossil taxa (Figure S2). Relative to using vaguely defined qualitative characters, we took the non-overlapping box-plots of anthropoids and prosimians and coded those values as 0 = “shallow”, MDI < 2.7; and 1 = “deep”, MDI > 2.7. *Darwinius*, *Notharctus*, and *Catopithecus* all fall within the extant prosimian range and were therefore coded as “0” along with all extant prosimian taxa. In addition, 2-tailed *t*-tests reveal that the mandibular depth of *Darwinius* (MDI = 1.7; see Table S5) is significantly shorter than that of anthropoids (*p* = 0.020), but not significantly different from that of Lorisoidea (*p* = 0.769) or Lemuroidea (*p* = 0.886).

**Character 11: Postorbital closure**

Both Franzen et al. ([S10]; their Table 3) and Gingerich et al. ([S1]; their supporting documents) distinguish “partial” closure and “complete” closure as separate conditions, yet combine them into one character state. Given that “partial” closure (i.e., the condition seen in *Tarsius*) and “complete” closure (as seen in modern anthropoids) are most commonly recognized as distinct character states (e.g., [S11]), the “partial” and “complete” closure states should be separated. Therefore, we have re-coded “postorbital closure” as an ordered character with three states: postorbital bar = 0; partial closure (*Tarsius* condition) = 1; full closure = 2 (anthropoid condition).

**Character 13: Mandibular symphysis fusion**

Ravosa (e.g., [S12-13]) has analyzed this character extensively among living and fossil primates. Similar to the situation with post-orbital closure, he recognizes that this appears to be an ordered character with three states: mandibular symphysis open = 0; mandibular symphysis partially fused = 1; mandibular symphysis fully fused = 2. Again, in the initial description of *Darwinius*, Franzen et al. ([S10]; their Table 3) recognize “partial” symphyseal fusion as a distinct condition and note that *Darwinius* possesses a partially fused mandibular symphysis. Gingerich et al. [S1], instead, only recognize two states: open = 0; fused = 1. This coding scheme ignores both the earlier studies by Ravosa [S12-13] as well as the initial description of *Darwinius* by Franzen et al. [S10]. Here, we follow the work of Ravosa [S12-13] and recognize mandibular symphysis fusion as an ordered three-state character.

Because symphyseal fusion is related to allometry, Ravosa [S12-13] has demonstrated that large prosimian taxa (e.g., large extant lemurs, subfossil lemurs, adapoids, and omomyoids) display varying degrees of symphyseal fusion. To reflect this reality, we have coded Lemuroidea and Tarsioidea (including omomyoids) with the multistate condition (“0/1”). *Darwinius* and *Catopithecus* display the partial fusion condition (“1”), while *Notharctus* displays both the partial fusion and full fusion condition (“1/2”) [S10, S12].

**Character 19: Lower molars**

This character was previously imprecisely defined. We re-define it here to explicitly refer to the presence and development of a paraconid on the trigonid of the lower molars. This character is ordered. Recognized states now include “present” = 0; “reduced” = 1; “absent” = 2. Extant strepsirhines, catarrhines, and *Darwinius* are recognized as lacking a paraconid on the lower molars. *Catopithecus* and *Notharctus* both display reduced paraconids. Ceboids display either reduced paraconids (some early fossil forms) or lack paraconids and were therefore coded as “1/2”. Tupaioids and tarsiioids exhibit unreduced paraconids on their lower molars (e.g., [S14-15]).

**Character 21: Fibular facet of astragalus slope**

*Darwinius* was changed from “0” to “?” based on results of Boyer et al. [S16]. These authors argue that *Darwinius* has not been quantitatively demonstrated to have the anthropoid or strepsirhine condition of this trait. Furthermore, they showed that accepted close relative of *Darwinius*, *Afradapis* [S17] has the strepsirhine condition of a sloping facet.

**Character 22: Pes condition**

*Tupaia* changed from “0” to “1” because it has a metatarsifulcrimating foot [S18, S19].

**Character 23: Mesocuneiform form**

Gingerich et al. [S1] claim that in lacking the compressed form of the mesocuneiform exhibited by strepsirhines and treeshrews, *Darwinius* is haplorhine-like. However, going back to Morton [S19] one finds reference to the compressed form of the mesocuneiform as a specifically lemurid trait. Further, Morton explicitly acknowledges the lack of this trait in *Notharctus* and likens *Notharctus* instead to “Lorises and Pottos” ([S19]: p. 25). Thus, we recode several taxa. *Tupaia* is changed from “0” to “1” to reflect that it lacks the compressed mesocuneiform of lemurids – as in *Darwinius*, in certain tupaioids, the mesocunieform is actually wider than the ectocuneiform (e.g., *Ptilocercus lowii* USNM 488055: mesocuneiform mediolateral width at distal end on dorsal surface = 0.97mm; ectocuneiform mediolateral width at equivalent position = 0.90mm). Lorisoidea is also changed from “0” to “1” again, to indicate its divergence from Lemuroidea in this trait. The expanded mesocuneiform is not a haplorhine synapomorphy.

**Character 24: Length of pedal digits**

*Tupaia* changed from “0” to “1” because its fourth digit is longest (Table 3 of Dagosto [S18]); *Darwinius* changed to “1” because its fourth digit cannot be the longest (see Appendix tables 15, 16, 18, and 20 in Franzen et al. [S10]). We presume Gingerich et al. [S1] gave this coding because the sum of the metatarsals and phalanx length from the cited tables adds to 45.8 mm for digit III and 45.5mm for digit IV. Morton [S19] seems to have considered this total length the defining character in his work. However, other authors have since considered the toe lengths only [S18]. In this case, toe III is only 28.7mm while toe IV is 33.2mm. This reveals that the difference is made up by a metatarsal IV measured at only 12.3mm long in *Darwinius*, which is shorter than its other metatarsals by 2.7mm(MtV) to 4.8mm(MtIII). This condition of having the MtIV so dramatically shortened compared to either MTIII and MtV is never seen in any primate of our 290 specimen, 39 species sample.

1. MtIII/MtIV: 1.39 (*Darwinius*) vs. 0.90-1.18 (Range for extant sample, n=290
2. MtIV/MtV: 0.82 (*Darwinius*) vs. 0.98-1.21 (Range for extant sample, n=290

Thus, there is some error in the reported measurements. Taking the most conservative estimate of the actual length of MtIV using the extant range it should be at least 14.5mm long (for an MtIII/MtIV ratio of 1.18) or 14.6mm (for an MtIV/MtV ratio of 0.975). In these most conservative cases, the total estimated length of digit IV comes to 47.7mm and 47.8mm, respectively – which is roughly 2mm longer than the pedal digit III. However, in order to avoid using erroneous or unknown measurements on MtIV, we conservatively use only the published toe lengths, as in Dagosto [S18]. Thus, *Darwinius* is scored as having state 1, “fourth digit longest”.

Ceboidea is also changed to “0/1” because their fourth digit is often, but not always, the longest (Table 3 of Dagosto [S18]).

**Character 25: Pedal digit II distal phalanx form**

Based on results of current study (see main text), the coding scheme of 25 was changed to an unordered three-state character: Falculae = 0; Grooming claw = 1; Ungulae = 2; *Darwinius* changed to “?”; all taxa originally coded with “0” except for *Tupaia* re-coded as “1”; all taxa originally coded with “1” recoded as “2”. Ceboidea is coded as polymorphic “1/2” due to the recent finding that *Aotus* and possibly some other ceboids have a grooming claw on pedal digit two [S20]. We refrain from ordering this character as the presence of wide apical tuft in the grooming claw of *Notharctus* renders the presumed morphocline linking claws, grooming claws and nails ambiguous.

**Section 3. Adding only *Notharctus* to the corrected matrix**

The result of the first analysis (*Notharctus* coded as having a grooming claw, *Darwinius* with “1/2” reflecting uncertainty: see section 11) is three most parsimonious trees, all of which put *Notharctus* followed by *Darwinius* as stem-haplorhines (Fig. S3A; TL = 45, CI = 0.8000, HI = 0.2000, RI = 0.8676, RC = 0.6941). The next analysis (*Notharctus* coded as having a grooming claw, *Darwinius* as lacking one: see section 12 for nexus file) yields four equally parsimonious trees. Three out of the four trees are identical to the three most parsimonious topologies from the first analysis. However, the fourth topology puts Tarsioidea in between *Notharctus* and *Darwinius* (Fig. S3B). The resulting trees also have an additional step (TL = 46 , CI = 0.7826, HI =0.2174, RI = 0.8551, RC = 0.6692). The third analysis (*Notharctus* and *Darwinius* coded as lacking grooming claws: see section 13 for nexus file) produces the same three topologies as in the first analysis, with tree stats identical to the second analysis (Fig. S3B: TL = 46, CI = 0.7826, HI =0.2174, RI = 0.8551, RC = 0.6692).

**Section 4. Character additions.**

**Characters added in file: original matrix corrected-pluschar31-39.nex (see Section 15 below)**

**Character 31: Flexor fibularis groove position (e.g., [S21])**

States for this character are “in-line with medial tibial facet” = 0; “lateral to medial tibial facet” = 1. *Tupaia* clearly has the “in-line” state, making it the primitive condition relative to this analysis. It is necessary to note that other euarchontans, including plesiadapiforms and dermopterans [S21] have a laterally positioned groove. Thus matrices with better outgroup sampling may resolve the polarity and evolution of this trait differently.

**Character 32: Posterior aspect of astragalus trochlea (e.g., [S18])**

States for this character are “unexpanded” = 0; “expanded into shelf” = 1. The derived state of this feature usually referred to as the “posterior trochlear shelf” is associated with leaping behaviors. Notably it is lacking in non-leaping lorisiforms and the *Darwinius* close-relative *Afradapis* [S16].

**Character 33: Peroneal tuberosity on mt1 [S18]**

States for this character are “reduced” = 0; “enlarged” = 1. The peroneus longus tendon attaches to this feature. All extant prosimians have been demonstrated to have the “enlarged” state [S22]. References in the literature to certain prosimian taxa such as tarsiers and lorises as having a relatively small process are in reference to other prosimians, not anthropoids. No extant prosimians have a relatively smaller process than any extant anthropoid. Patel et al. [S23] described the Mt1 of *Catopithecus* as having a small process.

**Character 34: Medial tibial facet [S18]**

States for this character are “shallow” = 0; “deep” = 1. Prosimians are recognized as having the “deep” state which is recognizable by the presence of two features: 1) the medial facet must come close to or succeed in touching the plantar facets (sustentacular facet or ribbon-like accessory extension of the sustentacular facet); 2) the dorsoventral depth of the medial side of the trochlea must approach or exceed that of the lateral side. Certain taxa exhibit the first feature without the second (e.g., tupaioids). Theoretically the second feature without the first could also be present, but this would likely be scored as “shallow” because the character refers to the facet specifically.

**Character 35: Hypoconulid lobe of M_3_ (e.g., [S24])**

The states for this character are “abbreviated” = 0; “developed” = 1. Tupaioids lack a well-developed hypoconulid lobe on m3. In this way they are like many anthropoids as well as dermopterans and various “insectivorans.” However, the presence of a well-developed lobe in plesidapiforms (as in strepsirhines, adapiforms, tarsioids, etc.) gives the opposite impression that the “developed” state is primitive for primates.

**Character 36: Cuboid facet of navicular contact [S25]**

States for this character are “only ectocuneiform facet” = 0; “both ecto- and mesocuneiform” facet = 1. To state the alternative conditions more explicitly, the cuboid facet of the navicular is usually adjacent to the ectocuneiform facet, which intercedes between the former facet and that for the mesocuneiform. This condition is present in anthropoids and *Tupaia* [S25]. However, strepsirhines and *Notharctus* are known to exhibit a configuration in which both them ectocuneiform and mesocuneiform facet touch the cuboid facet on the navicular. In AMNH 143612, the navicular is lacking but the fact that, when in articulation (main text Fig. 4), the mesocuneiform reaches around behind the ectocuneiform (on the plantar side) to reach the cuboid, dictates the presence of the derived state in this individual too. *Darwinius* was described as exhibiting this trait as well.

“The navicular is situated between the talus proximally and the ecto- and mesocuneiform distally. It is a long bone compared to that in lorisines, indriids and anthropoids [[34]](http://www.plosone.org/article/info:doi/10.1371/journal.pone.0005723#pone.0005723-Gebo1%23pone.0005723-Gebo1), and it is more like that of Hapalemur and Eulemur, although it is not as wide. The naviculocuboid articulation is broad and contiguous with both the ectocuneiform and mesocuneiform facets shaped like those of living lemuriforms and all known notharctines” (Franzen et al. [S10])

**Character 37: Divergence of big toe [S22]**

This character is ordered. Recognized states include “not divergent” = 0; “moderate divergence” = 1; “extreme divergence” =2. We code *Catopithecus* on the basis of Patel et al. [S23].

**Character 38: Orbit diameter/Activity pattern- [S26]**

This character is ordered. Recognized states include large/nocturnal = 0; moderate/cathemeral = 1; small/diurnal = 2. As described by Franzen et al. [S10], *Darwinius* is considered to have large orbits and a nocturnal activity pattern.

**Character 39: Medial malleolus rotation- [S25]**

This character is ordered with three states: no rotation = 0, slight rotation = 1, marked rotation = 2. A high degree of rotation is a derived strepsirhine trait, with haplorhines only being slightly rotated. Gregory ([S27]: p.93) states that “the tibia of *Notharctus* is essentially lemurine,” and further on (p.94), that in part, *Cebus* differs from *Notharctus* in that “the internal malleolus (of C*ebus*) is more produced downward, the distal end as seen from the rear is wider and flatter at the lower edge.” Dagosto ([S28]; her Table 2) notes that the primitive eutherian mammal condition (including insectivores, creodonts and carnivores) is no rotation, and then states this: "Some archontans approach the conditions observed in euprimates. *Tupaia*, but not *Ptilocercus*, *Lyonogale*, or *Urogale*, also has a long, triangular medial malleolus which is distally convex and slightly rotated.”

**Section 5. Taxon additions.**

***Notharctus* and *Catopithecus* added in file: Original matrix corrected-pluschar31-39-noth-cato-added.nex (see Section 18 below)**

We added the fossil taxa *Notharctus* and *Catopithecus*. We provide the matrix below (Table S6) for quick reference. Highlighted cells show missing data. Note that *Darwinius* has more missing data than the other fossils in this matrix.

**Section 6. darwinius-original_nothadded_gclaw.nex**

#NEXUS

BEGIN TAXA;

TITLE Taxa;

DIMENSIONS NTAX=9;

TAXLABELS

TUPAIOIDEA LEMUROIDEA LORISOIDEA TARSIOIDEA CEBOIDEA CERCOPITH. HOMINOIDEA Darwinius Notharctus

;

END;

BEGIN CHARACTERS;

TITLE Character_Matrix;

DIMENSIONS NCHAR=30;

FORMAT DATATYPE = STANDARD GAP = - MISSING = ? SYMBOLS = " 0 1 2";

CHARSTATELABELS

1 Nose_and_upper_lip / Wet_cleft Dry_continuous, 2 Jacobsons_vomeronasal_organ / Present Absent, 3 Sphenoidal_recess / Substantial Reduced, 4 Eye / Reflecting '-' tapetum '-' lucida Retinal_fovea, 5 Brain_and_braincase / Rel_small Rel_large, 6 Olfactory_bulbs / Rel_large Rel_small, 7 Blood_supply_to_brain / Complex Promontory_artery, 8 Cranial_rostrum / Rel_long Rel_short, 9 Mandibular_ramus / Shallow Deep, 10 Metopic_suture_uniting_frontals / Open Fused, 11 Postorbital_closure / None Partial_to_complete, 12 Ectotympanic / Free Lateral_wall Tubular, 13 Mandibular_symphysis / Open Fused, 14 Incisors / Pointed_procumbent Vertical_spatulate, 15 Canines / Procumbent Vertical_interlocking, 16 Canine_dimorphism / Absent Present, 17 Upper_molars / Tritubercular Quadrate_hypocone, 18 'Premolar P/4 ' / Elongated Transverse_pad '-' mcd, 19 Lower_molars / Tritubercular Quadrate, 20 'Capitate (os magnum)' / Compressed Uncompressed, 21 Fibular_facet_on_astragalus / Sloping Rel_steep, 22 Pes / Tarsi_fulcr. Metatar_fulcr., 23 Mesocuneiform / Compressed Uncompressed, 24 Longest_toe_of_pes / Third Fourth, 25 Claws_or_grooming_claws / Present Absent, 26 Mammary_glands / Multiple Pair, 27 Uterus / Bicornate Simplex, 28 Placenta / Epitheliochorial Hemochorial, 29 'Precociality (teeth at birth)' / More Less, 30 'SINE human Alu transpositions C7, C9, C12' / Absent Present ;

MATRIX

TUPAIOIDEA 000000000000000010001000000000

LEMUROIDEA 000000000000000010100001000000

LORISOIDEA 000000000001000010100001000000

TARSIOIDEA 101100110012001000011011000101

CEBOIDEA 101111111111111111111110111101

CERCOPITH. 111111111112111111111110111111

HOMINOIDEA 111111111112111111111110111111

Darwinius ????0??11000111?1?1?10101?????

Notharctus ????000000001111101000110?????

;

END;

BEGIN ASSUMPTIONS;

TYPESET * UNTITLED = unord: 1 - 30;

EXSET * UNTITLED = ;

WTSET * UNTITLED = 1: 1 - 30 ;

END;

**Section 7. darwinius-original_nothadded_gclawabsent.nex**

#NEXUS

BEGIN TAXA;

TITLE Taxa;

DIMENSIONS NTAX=9;

TAXLABELS

TUPAIOIDEA LEMUROIDEA LORISOIDEA TARSIOIDEA CEBOIDEA CERCOPITH. HOMINOIDEA Darwinius Notharctus

;

END;

BEGIN CHARACTERS;

TITLE Character_Matrix;

DIMENSIONS NCHAR=30;

FORMAT DATATYPE = STANDARD GAP = - MISSING = ? SYMBOLS = " 0 1 2";

CHARSTATELABELS

1 Nose_and_upper_lip / Wet_cleft Dry_continuous, 2 Jacobsons_vomeronasal_organ / Present Absent, 3 Sphenoidal_recess / Substantial Reduced, 4 Eye / Reflecting '-' tapetum '-' lucida Retinal_fovea, 5 Brain_and_braincase / Rel_small Rel_large, 6 Olfactory_bulbs / Rel_large Rel_small, 7 Blood_supply_to_brain / Complex Promontory_artery, 8 Cranial_rostrum / Rel_long Rel_short, 9 Mandibular_ramus / Shallow Deep, 10 Metopic_suture_uniting_frontals / Open Fused, 11 Postorbital_closure / None Partial_to_complete, 12 Ectotympanic / Free Lateral_wall Tubular, 13 Mandibular_symphysis / Open Fused, 14 Incisors / Pointed_procumbent Vertical_spatulate, 15 Canines / Procumbent Vertical_interlocking, 16 Canine_dimorphism / Absent Present, 17 Upper_molars / Tritubercular Quadrate_hypocone, 18 'Premolar P/4 ' / Elongated Transverse_pad '-' mcd, 19 Lower_molars / Tritubercular Quadrate, 20 'Capitate (os magnum)' / Compressed Uncompressed, 21 Fibular_facet_on_astragalus / Sloping Rel_steep, 22 Pes / Tarsi_fulcr. Metatar_fulcr., 23 Mesocuneiform / Compressed Uncompressed, 24 Longest_toe_of_pes / Third Fourth, 25 Claws_or_grooming_claws / Present Absent, 26 Mammary_glands / Multiple Pair, 27 Uterus / Bicornate Simplex, 28 Placenta / Epitheliochorial Hemochorial, 29 'Precociality (teeth at birth)' / More Less, 30 'SINE human Alu transpositions C7, C9, C12' / Absent Present ;

MATRIX

TUPAIOIDEA 000000000000000010001000000000

LEMUROIDEA 000000000000000010100001000000

LORISOIDEA 000000000001000010100001000000

TARSIOIDEA 101100110012001000011011000101

CEBOIDEA 101111111111111111111110111101

CERCOPITH. 111111111112111111111110111111

HOMINOIDEA 111111111112111111111110111111

Darwinius ????0??11000111?1?1?10101?????

Notharctus ????000000001111101000111?????

;

END;

BEGIN ASSUMPTIONS;

TYPESET * UNTITLED = unord: 1 - 30;

EXSET * UNTITLED = ;

WTSET * UNTITLED = 1: 1 - 30 ;

END;

**Section 8. darwinius-original_noth&catoadded_gclaw.nex**

#NEXUS

BEGIN TAXA;

TITLE Taxa;

DIMENSIONS NTAX=10;

TAXLABELS

TUPAIOIDEA LEMUROIDEA LORISOIDEA TARSIOIDEA CEBOIDEA CERCOPITH. HOMINOIDEA Darwinius Notharctus Catopithecus

;

END;

BEGIN CHARACTERS;

TITLE Character_Matrix;

DIMENSIONS NCHAR=30;

FORMAT DATATYPE = STANDARD GAP = - MISSING = ? SYMBOLS = " 0 1 2";

CHARSTATELABELS

1 Nose_and_upper_lip / Wet_cleft Dry_continuous, 2 Jacobsons_vomeronasal_organ / Present Absent, 3 Sphenoidal_recess / Substantial Reduced, 4 Eye / Reflecting '-' tapetum '-' lucida Retinal_fovea, 5 Brain_and_braincase / Rel_small Rel_large, 6 Olfactory_bulbs / Rel_large Rel_small, 7 Blood_supply_to_brain / Complex Promontory_artery, 8 Cranial_rostrum / Rel_long Rel_short, 9 Mandibular_ramus / Shallow Deep, 10 Metopic_suture_uniting_frontals / Open Fused, 11 Postorbital_closure / None Partial_to_complete, 12 Ectotympanic / Free Lateral_wall Tubular, 13 Mandibular_symphysis / Open Fused, 14 Incisors / Pointed_procumbent Vertical_spatulate, 15 Canines / Procumbent Vertical_interlocking, 16 Canine_dimorphism / Absent Present, 17 Upper_molars / Tritubercular Quadrate_hypocone, 18 'Premolar P/4 ' / Elongated Transverse_pad '-' mcd, 19 Lower_molars / Tritubercular Quadrate, 20 'Capitate (os magnum)' / Compressed Uncompressed, 21 Fibular_facet_on_astragalus / Sloping Rel_steep, 22 Pes / Tarsi_fulcr. Metatar_fulcr., 23 Mesocuneiform / Compressed Uncompressed, 24 Longest_toe_of_pes / Third Fourth, 25 Claws_or_grooming_claws / Present Absent, 26 Mammary_glands / Multiple Pair, 27 Uterus / Bicornate Simplex, 28 Placenta / Epitheliochorial Hemochorial, 29 'Precociality (teeth at birth)' / More Less, 30 'SINE human Alu transpositions C7, C9, C12' / Absent Present ;

MATRIX

TUPAIOIDEA 000000000000000010001000000000

LEMUROIDEA 000000000000000010100001000000

LORISOIDEA 000000000001000010100001000000

TARSIOIDEA 101100110012001000011011000101

CEBOIDEA 101111111111111111111110111101

CERCOPITH. 111111111112111111111110111111

HOMINOIDEA 111111111112111111111110111111

Darwinius ????0??11000111?1?1?10101?????

Notharctus ????000000001111101000110?????

Catopithecus ????011101110111111?1?????????

;

END;

BEGIN ASSUMPTIONS;

TYPESET * UNTITLED = unord: 1 - 30;

EXSET * UNTITLED = ;

WTSET * UNTITLED = 1: 1 - 30 ;

END;

**Section 9. darwinius-original_noth&catoadded_gclawabsent.nex**

#NEXUS

BEGIN TAXA;

TITLE Taxa;

DIMENSIONS NTAX=10;

TAXLABELS

TUPAIOIDEA LEMUROIDEA LORISOIDEA TARSIOIDEA CEBOIDEA CERCOPITH. HOMINOIDEA Darwinius Notharctus Catopithecus

;

END;

BEGIN CHARACTERS;

TITLE Character_Matrix;

DIMENSIONS NCHAR=30;

FORMAT DATATYPE = STANDARD GAP = - MISSING = ? SYMBOLS = " 0 1 2";

CHARSTATELABELS

1 Nose_and_upper_lip / Wet_cleft Dry_continuous, 2 Jacobsons_vomeronasal_organ / Present Absent, 3 Sphenoidal_recess / Substantial Reduced, 4 Eye / Reflecting '-' tapetum '-' lucida Retinal_fovea, 5 Brain_and_braincase / Rel_small Rel_large, 6 Olfactory_bulbs / Rel_large Rel_small, 7 Blood_supply_to_brain / Complex Promontory_artery, 8 Cranial_rostrum / Rel_long Rel_short, 9 Mandibular_ramus / Shallow Deep, 10 Metopic_suture_uniting_frontals / Open Fused, 11 Postorbital_closure / None Partial_to_complete, 12 Ectotympanic / Free Lateral_wall Tubular, 13 Mandibular_symphysis / Open Fused, 14 Incisors / Pointed_procumbent Vertical_spatulate, 15 Canines / Procumbent Vertical_interlocking, 16 Canine_dimorphism / Absent Present, 17 Upper_molars / Tritubercular Quadrate_hypocone, 18 'Premolar P/4 ' / Elongated Transverse_pad '-' mcd, 19 Lower_molars / Tritubercular Quadrate, 20 'Capitate (os magnum)' / Compressed Uncompressed, 21 Fibular_facet_on_astragalus / Sloping Rel_steep, 22 Pes / Tarsi_fulcr. Metatar_fulcr., 23 Mesocuneiform / Compressed Uncompressed, 24 Longest_toe_of_pes / Third Fourth, 25 Claws_or_grooming_claws / Present Absent, 26 Mammary_glands / Multiple Pair, 27 Uterus / Bicornate Simplex, 28 Placenta / Epitheliochorial Hemochorial, 29 'Precociality (teeth at birth)' / More Less, 30 'SINE human Alu transpositions C7, C9, C12' / Absent Present ;

MATRIX

TUPAIOIDEA 000000000000000010001000000000

LEMUROIDEA 000000000000000010100001000000

LORISOIDEA 000000000001000010100001000000

TARSIOIDEA 101100110012001000011011000101

CEBOIDEA 101111111111111111111110111101

CERCOPITH. 111111111112111111111110111111

HOMINOIDEA 111111111112111111111110111111

Darwinius ????0??11000111?1?1?10101?????

Notharctus ????000000001111101000111?????

Catopithecus ????011101110111111?1?????????

;

END;

BEGIN ASSUMPTIONS;

TYPESET * UNTITLED = unord: 1 - 30;

EXSET * UNTITLED = ;

WTSET * UNTITLED = 1: 1 - 30 ;

END;

**Section 10. original matrix corrected.nex**

#NEXUS

BEGIN TAXA;

TITLE Taxa;

DIMENSIONS NTAX=8;

TAXLABELS

TUPAIOIDEA LEMUROIDEA LORISOIDEA TARSIOIDEA CEBOIDEA CERCOPITH. HOMINOIDEA Darwinius

;

END;

BEGIN CHARACTERS;

TITLE Character_Matrix;

DIMENSIONS NCHAR=30;

FORMAT DATATYPE = STANDARD GAP = - MISSING = ? SYMBOLS = " 0 1 2";

CHARSTATELABELS

1 Nose_and_upper_lip / Wet_cleft Dry_continuous, 2 Jacobsons_vomeronasal_organ / Present Absent, 3 Sphenoidal_recess / Substantial Reduced, 4 Eye / Reflecting '-' tapetum '-' lucida Retinal_fovea, 5 Brain_and_braincase / Rel_small Rel_large, 6 Olfactory_bulbs / Rel_large Intermediate Rel_small, 7 Blood_supply_to_brain / Complex Promontory_artery, 8 Cranial_rostrum / Rel_long Rel_short, 9 Mandibular_ramus / Shallow Deep, 10 Metopic_suture_uniting_frontals / Open Fused, 11 Postorbital_closure / None Partial Complete, 12 Ectotympanic / Free Lateral_wall Tubular, 13 Mandibular_symphysis / Open Partial Fused, 14 Incisors / Pointed_procumbent Vertical_spatulate, 15 Canines / Procumbent Vertical_interlocking, 16 Canine_dimorphism / Absent Present, 17 Upper_molars / Tritubercular Quadrate_hypocone, 18 'Premolar P/4 ' / Elongated Transverse_pad '-' mcd, 19 Paraconid_on_lower_molars / Present Reduced Absent, 20 'Capitate (os magnum)' / Compressed Uncompressed, 21 Fibular_facet_on_astragalus / Sloping Rel_steep, 22 Pes / Tarsi_fulcr. Metatar_fulcr., 23 Mesocuneiform / Compressed Uncompressed, 24 Longest_toe_of_pes / Third Fourth, 25 Claws_or_grooming_claws / Claws Grooming_claws Absent, 26 Mammary_glands / Multiple Pair, 27 Uterus / Bicornate Simplex, 28 Placenta / Epitheliochorial Hemochorial, 29 'Precociality (teeth at birth)' / More Less, 30 'SINE human Alu transpositions C7, C9, C12' / Absent Present ;

MATRIX

TUPAIOIDEA 000000000000000010001111000000

LEMUROIDEA 000000000000{0 1}00010200001100000

LORISOIDEA 000000000001000010200011100000

TARSIOIDEA 101100110012{0 1}01000011011100101

CEBOIDEA 101112111121211111{1 2}1111{0 1}{1 2}11101

CERCOPITH. 111112111122211111211110211111

HOMINOIDEA 111112111122211111211110211111

Darwinius ????0??10000111?1?2??011{1 2}?????

;

END;

BEGIN ASSUMPTIONS;

TYPESET * UNTITLED = unord: 25, ord: 1 - 24 26 - 30;

EXSET * UNTITLED = ;

WTSET * UNTITLED = 1: 1 - 30 ;

END;

**Section 11. original matrix corrected_noth-added_v1.nex**

#NEXUS

BEGIN TAXA;

TITLE Taxa;

DIMENSIONS NTAX=9;

TAXLABELS

TUPAIOIDEA LEMUROIDEA LORISOIDEA TARSIOIDEA CEBOIDEA CERCOPITH. HOMINOIDEA Darwinius Notharctus

;

END;

BEGIN CHARACTERS;

TITLE Character_Matrix;

DIMENSIONS NCHAR=30;

FORMAT DATATYPE = STANDARD GAP = - MISSING = ? SYMBOLS = " 0 1 2";

CHARSTATELABELS

1 Nose_and_upper_lip / Wet_cleft Dry_continuous, 2 Jacobsons_vomeronasal_organ / Present Absent, 3 Sphenoidal_recess / Substantial Reduced, 4 Eye / Reflecting '-' tapetum '-' lucida Retinal_fovea, 5 Brain_and_braincase / Rel_small Rel_large, 6 Olfactory_bulbs / Rel_large Intermediate Rel_small, 7 Blood_supply_to_brain / Complex Promontory_artery, 8 Cranial_rostrum / Rel_long Rel_short, 9 Mandibular_ramus / Shallow Deep, 10 Metopic_suture_uniting_frontals / Open Fused, 11 Postorbital_closure / None Partial Complete, 12 Ectotympanic / Free Lateral_wall Tubular, 13 Mandibular_symphysis / Open Partial Fused, 14 Incisors / Pointed_procumbent Vertical_spatulate, 15 Canines / Procumbent Vertical_interlocking, 16 Canine_dimorphism / Absent Present, 17 Upper_molars / Tritubercular Quadrate_hypocone, 18 'Premolar P/4 ' / Elongated Transverse_pad '-' mcd, 19 Paraconid_on_lower_molars / present reduced absent, 20 'Capitate (os magnum)' / Compressed Uncompressed, 21 Fibular_facet_on_astragalus / Sloping Rel_steep, 22 Pes / Tarsi_fulcr. Metatar_fulcr., 23 Mesocuneiform / Compressed Uncompressed, 24 Longest_toe_of_pes / Third Fourth, 25 Claws_or_grooming_claws / Claws Grooming_claws Absent, 26 Mammary_glands / Multiple Pair, 27 Uterus / Bicornate Simplex, 28 Placenta / Epitheliochorial Hemochorial, 29 'Precociality (teeth at birth)' / More Less, 30 'SINE human Alu transpositions C7, C9, C12' / Absent Present ;

MATRIX

TUPAIOIDEA 000000000000000010001111000000

LEMUROIDEA 000000000000{0 1}00010200001100000

LORISOIDEA 000000000001000010200011100000

TARSIOIDEA 101100110012{0 1}01000011011100101

CEBOIDEA 101112111121211111{1 2}1111{0 1}{1 2}11101

CERCOPITH. 111112111122211111211110211111

HOMINOIDEA 111112111122211111211110211111

Darwinius ????0??10000111?1?2??011{1 2}?????

Notharctus ????00000000{1 2}111101000111?????

;

END;

BEGIN ASSUMPTIONS;

TYPESET * UNTITLED = unord: 25, ord: 1 - 24 26 - 30;

EXSET * UNTITLED = ;

WTSET * UNTITLED = 1: 1 - 30 ;

END;

**Section 12. original matrix corrected_noth-added_v2.nex**

#NEXUS

BEGIN TAXA;

TITLE Taxa;

DIMENSIONS NTAX=9;

TAXLABELS

TUPAIOIDEA LEMUROIDEA LORISOIDEA TARSIOIDEA CEBOIDEA CERCOPITH. HOMINOIDEA Darwinius Notharctus

;

END;

BEGIN CHARACTERS;

TITLE Character_Matrix;

DIMENSIONS NCHAR=30;

FORMAT DATATYPE = STANDARD GAP = - MISSING = ? SYMBOLS = " 0 1 2";

CHARSTATELABELS

1 Nose_and_upper_lip / Wet_cleft Dry_continuous, 2 Jacobsons_vomeronasal_organ / Present Absent, 3 Sphenoidal_recess / Substantial Reduced, 4 Eye / Reflecting '-' tapetum '-' lucida Retinal_fovea, 5 Brain_and_braincase / Rel_small Rel_large, 6 Olfactory_bulbs / Rel_large Intermediate Rel_small, 7 Blood_supply_to_brain / Complex Promontory_artery, 8 Cranial_rostrum / Rel_long Rel_short, 9 Mandibular_ramus / Shallow Deep, 10 Metopic_suture_uniting_frontals / Open Fused, 11 Postorbital_closure / None Partial Complete, 12 Ectotympanic / Free Lateral_wall Tubular, 13 Mandibular_symphysis / Open Partial Fused, 14 Incisors / Pointed_procumbent Vertical_spatulate, 15 Canines / Procumbent Vertical_interlocking, 16 Canine_dimorphism / Absent Present, 17 Upper_molars / Tritubercular Quadrate_hypocone, 18 'Premolar P/4 ' / Elongated Transverse_pad '-' mcd, 19 Paraconid_on_lower_molars / present reduced absent, 20 'Capitate (os magnum)' / Compressed Uncompressed, 21 Fibular_facet_on_astragalus / Sloping Rel_steep, 22 Pes / Tarsi_fulcr. Metatar_fulcr., 23 Mesocuneiform / Compressed Uncompressed, 24 Longest_toe_of_pes / Third Fourth, 25 Claws_or_grooming_claws / Claws Grooming_claws Absent, 26 Mammary_glands / Multiple Pair, 27 Uterus / Bicornate Simplex, 28 Placenta / Epitheliochorial Hemochorial, 29 'Precociality (teeth at birth)' / More Less, 30 'SINE human Alu transpositions C7, C9, C12' / Absent Present ;

MATRIX

TUPAIOIDEA 000000000000000010001111000000

LEMUROIDEA 000000000000{0 1}00010200001100000

LORISOIDEA 000000000001000010200011100000

TARSIOIDEA 101100110012{0 1}01000011011100101

CEBOIDEA 101112111121211111{1 2}1111{0 1}{1 2}11101

CERCOPITH. 111112111122211111211110211111

HOMINOIDEA 111112111122211111211110211111

Darwinius ????0??10000111?1?2??0112?????

Notharctus ????00000000{1 2}111101000111?????

;

END;

BEGIN ASSUMPTIONS;

TYPESET * UNTITLED = unord: 25, ord: 1 - 24 26 - 30;

EXSET * UNTITLED = ;

WTSET * UNTITLED = 1: 1 - 30 ;

END;

**Section 13. original matrix corrected_noth-added_v3.nex**

#NEXUS

BEGIN TAXA;

TITLE Taxa;

DIMENSIONS NTAX=9;

TAXLABELS

TUPAIOIDEA LEMUROIDEA LORISOIDEA TARSIOIDEA CEBOIDEA CERCOPITH. HOMINOIDEA Darwinius Notharctus

;

END;

BEGIN CHARACTERS;

TITLE Character_Matrix;

DIMENSIONS NCHAR=30;

FORMAT DATATYPE = STANDARD GAP = - MISSING = ? SYMBOLS = " 0 1 2";

CHARSTATELABELS

1 Nose_and_upper_lip / Wet_cleft Dry_continuous, 2 Jacobsons_vomeronasal_organ / Present Absent, 3 Sphenoidal_recess / Substantial Reduced, 4 Eye / Reflecting '-' tapetum '-' lucida Retinal_fovea, 5 Brain_and_braincase / Rel_small Rel_large, 6 Olfactory_bulbs / Rel_large Intermediate Rel_small, 7 Blood_supply_to_brain / Complex Promontory_artery, 8 Cranial_rostrum / Rel_long Rel_short, 9 Mandibular_ramus / Shallow Deep, 10 Metopic_suture_uniting_frontals / Open Fused, 11 Postorbital_closure / None Partial Complete, 12 Ectotympanic / Free Lateral_wall Tubular, 13 Mandibular_symphysis / Open Partial Fused, 14 Incisors / Pointed_procumbent Vertical_spatulate, 15 Canines / Procumbent Vertical_interlocking, 16 Canine_dimorphism / Absent Present, 17 Upper_molars / Tritubercular Quadrate_hypocone, 18 'Premolar P/4 ' / Elongated Transverse_pad '-' mcd, 19 Paraconid_on_lower_molars / present reduced absent, 20 'Capitate (os magnum)' / Compressed Uncompressed, 21 Fibular_facet_on_astragalus / Sloping Rel_steep, 22 Pes / Tarsi_fulcr. Metatar_fulcr., 23 Mesocuneiform / Compressed Uncompressed, 24 Longest_toe_of_pes / Third Fourth, 25 Claws_or_grooming_claws / Claws Grooming_claws Absent, 26 Mammary_glands / Multiple Pair, 27 Uterus / Bicornate Simplex, 28 Placenta / Epitheliochorial Hemochorial, 29 'Precociality (teeth at birth)' / More Less, 30 'SINE human Alu transpositions C7, C9, C12' / Absent Present ;

MATRIX

TUPAIOIDEA 000000000000000010001111000000

LEMUROIDEA 000000000000{0 1}00010200001100000

LORISOIDEA 000000000001000010200011100000

TARSIOIDEA 101100110012{0 1}01000011011100101

CEBOIDEA 101112111121211111{1 2}1111{0 1}{1 2}11101

CERCOPITH. 111112111122211111211110211111

HOMINOIDEA 111112111122211111211110211111

Darwinius ????0??10000111?1?2??0112?????

Notharctus ????00000000{1 2}111101000112?????

;

END;

BEGIN ASSUMPTIONS;

TYPESET * UNTITLED = unord: 25, ord: 1 - 24 26 - 30;

EXSET * UNTITLED = ;

WTSET * UNTITLED = 1: 1 - 30 ;

END;

**Section 14. original matrix corrected_noth&cato-added_v4.nex**

#NEXUS

BEGIN TAXA;

TITLE Taxa;

DIMENSIONS NTAX=10;

TAXLABELS

TUPAIOIDEA LEMUROIDEA LORISOIDEA TARSIOIDEA CEBOIDEA CERCOPITH. HOMINOIDEA Darwinius Notharctus Catopithecus

;

END;

BEGIN CHARACTERS;

TITLE Character_Matrix;

DIMENSIONS NCHAR=30;

FORMAT DATATYPE = STANDARD GAP = - MISSING = ? SYMBOLS = " 0 1 2";

CHARSTATELABELS

1 Nose_and_upper_lip / Wet_cleft Dry_continuous, 2 Jacobsons_vomeronasal_organ / Present Absent, 3 Sphenoidal_recess / Substantial Reduced, 4 Eye / Reflecting '-' tapetum '-' lucida Retinal_fovea, 5 Brain_and_braincase / Rel_small Rel_large, 6 Olfactory_bulbs / Rel_large Intermediate Rel_small, 7 Blood_supply_to_brain / Complex Promontory_artery, 8 Cranial_rostrum / Rel_long Rel_short, 9 Mandibular_ramus / Shallow Deep, 10 Metopic_suture_uniting_frontals / Open Fused, 11 Postorbital_closure / None Partial Complete, 12 Ectotympanic / Free Lateral_wall Tubular, 13 Mandibular_symphysis / Open Partial Fused, 14 Incisors / Pointed_procumbent Vertical_spatulate, 15 Canines / Procumbent Vertical_interlocking, 16 Canine_dimorphism / Absent Present, 17 Upper_molars / Tritubercular Quadrate_hypocone, 18 'Premolar P/4 ' / Elongated Transverse_pad '-' mcd, 19 Paraconid_on_lower_molars / present reduced absent, 20 'Capitate (os magnum)' / Compressed Uncompressed, 21 Fibular_facet_on_astragalus / Sloping Rel_steep, 22 Pes / Tarsi_fulcr. Metatar_fulcr., 23 Mesocuneiform / Compressed Uncompressed, 24 Longest_toe_of_pes / Third Fourth, 25 Claws_or_grooming_claws / Claws Grooming_claws Absent, 26 Mammary_glands / Multiple Pair, 27 Uterus / Bicornate Simplex, 28 Placenta / Epitheliochorial Hemochorial, 29 'Precociality (teeth at birth)' / More Less, 30 'SINE human Alu transpositions C7, C9, C12' / Absent Present ;

MATRIX

TUPAIOIDEA 000000000000000010001111000000

LEMUROIDEA 000000000000{0 1}00010200001100000

LORISOIDEA 000000000001000010200011100000

TARSIOIDEA 101100110012{0 1}01000011011100101

CEBOIDEA 101112111121211111{1 2}1111{0 1}{1 2}11101

CERCOPITH. 111112111122211111211110211111

HOMINOIDEA 111112111122211111211110211111

Darwinius ????0??10000111?1?2??011{1 2}?????

Notharctus ????00000000{1 2}111101000111?????

Catopithecus ????011101211111111?1?????????

;

END;

BEGIN ASSUMPTIONS;

TYPESET * UNTITLED = unord: 25, ord: 1 - 24 26 - 30;

EXSET * UNTITLED = ;

WTSET * UNTITLED = 1: 1 - 30 ;

END;

**Section 15. original matrix corrected_noth&cato-added_v5.nex**

#NEXUS

BEGIN TAXA;

TITLE Taxa;

DIMENSIONS NTAX=10;

TAXLABELS

TUPAIOIDEA LEMUROIDEA LORISOIDEA TARSIOIDEA CEBOIDEA CERCOPITH. HOMINOIDEA Darwinius Notharctus Catopithecus

;

END;

BEGIN CHARACTERS;

TITLE Character_Matrix;

DIMENSIONS NCHAR=30;

FORMAT DATATYPE = STANDARD GAP = - MISSING = ? SYMBOLS = " 0 1 2";

CHARSTATELABELS

1 Nose_and_upper_lip / Wet_cleft Dry_continuous, 2 Jacobsons_vomeronasal_organ / Present Absent, 3 Sphenoidal_recess / Substantial Reduced, 4 Eye / Reflecting '-' tapetum '-' lucida Retinal_fovea, 5 Brain_and_braincase / Rel_small Rel_large, 6 Olfactory_bulbs / Rel_large Intermediate Rel_small, 7 Blood_supply_to_brain / Complex Promontory_artery, 8 Cranial_rostrum / Rel_long Rel_short, 9 Mandibular_ramus / Shallow Deep, 10 Metopic_suture_uniting_frontals / Open Fused, 11 Postorbital_closure / None Partial Complete, 12 Ectotympanic / Free Lateral_wall Tubular, 13 Mandibular_symphysis / Open Partial Fused, 14 Incisors / Pointed_procumbent Vertical_spatulate, 15 Canines / Procumbent Vertical_interlocking, 16 Canine_dimorphism / Absent Present, 17 Upper_molars / Tritubercular Quadrate_hypocone, 18 'Premolar P/4 ' / Elongated Transverse_pad '-' mcd, 19 Paraconid_on_lower_molars / present reduced absent, 20 'Capitate (os magnum)' / Compressed Uncompressed, 21 Fibular_facet_on_astragalus / Sloping Rel_steep, 22 Pes / Tarsi_fulcr. Metatar_fulcr., 23 Mesocuneiform / Compressed Uncompressed, 24 Longest_toe_of_pes / Third Fourth, 25 Claws_or_grooming_claws / Claws Grooming_claws Absent, 26 Mammary_glands / Multiple Pair, 27 Uterus / Bicornate Simplex, 28 Placenta / Epitheliochorial Hemochorial, 29 'Precociality (teeth at birth)' / More Less, 30 'SINE human Alu transpositions C7, C9, C12' / Absent Present ;

MATRIX

TUPAIOIDEA 000000000000000010001111000000

LEMUROIDEA 000000000000{0 1}00010200001100000

LORISOIDEA 000000000001000010200011100000

TARSIOIDEA 101100110012{0 1}01000011011100101

CEBOIDEA 101112111121211111{1 2}1111{0 1}{1 2}11101

CERCOPITH. 111112111122211111211110211111

HOMINOIDEA 111112111122211111211110211111

Darwinius ????0??10000111?1?2??0112?????

Notharctus ????00000000{1 2}111101000111?????

Catopithecus ????011101211111111?1?????????

;

END;

BEGIN ASSUMPTIONS;

TYPESET * UNTITLED = unord: 25, ord: 1 - 24 26 - 30;

EXSET * UNTITLED = ;

WTSET * UNTITLED = 1: 1 - 30 ;

END;

**Section 16. original matrix corrected_noth&cato-added_v6.nex**

#NEXUS

BEGIN TAXA;

TITLE Taxa;

DIMENSIONS NTAX=10;

TAXLABELS

TUPAIOIDEA LEMUROIDEA LORISOIDEA TARSIOIDEA CEBOIDEA CERCOPITH. HOMINOIDEA Darwinius Notharctus Catopithecus

;

END;

BEGIN CHARACTERS;

TITLE Character_Matrix;

DIMENSIONS NCHAR=30;

FORMAT DATATYPE = STANDARD GAP = - MISSING = ? SYMBOLS = " 0 1 2";

CHARSTATELABELS

1 Nose_and_upper_lip / Wet_cleft Dry_continuous, 2 Jacobsons_vomeronasal_organ / Present Absent, 3 Sphenoidal_recess / Substantial Reduced, 4 Eye / Reflecting '-' tapetum '-' lucida Retinal_fovea, 5 Brain_and_braincase / Rel_small Rel_large, 6 Olfactory_bulbs / Rel_large Intermediate Rel_small, 7 Blood_supply_to_brain / Complex Promontory_artery, 8 Cranial_rostrum / Rel_long Rel_short, 9 Mandibular_ramus / Shallow Deep, 10 Metopic_suture_uniting_frontals / Open Fused, 11 Postorbital_closure / None Partial Complete, 12 Ectotympanic / Free Lateral_wall Tubular, 13 Mandibular_symphysis / Open Partial Fused, 14 Incisors / Pointed_procumbent Vertical_spatulate, 15 Canines / Procumbent Vertical_interlocking, 16 Canine_dimorphism / Absent Present, 17 Upper_molars / Tritubercular Quadrate_hypocone, 18 'Premolar P/4 ' / Elongated Transverse_pad '-' mcd, 19 Paraconid_on_lower_molars / present reduced absent, 20 'Capitate (os magnum)' / Compressed Uncompressed, 21 Fibular_facet_on_astragalus / Sloping Rel_steep, 22 Pes / Tarsi_fulcr. Metatar_fulcr., 23 Mesocuneiform / Compressed Uncompressed, 24 Longest_toe_of_pes / Third Fourth, 25 Claws_or_grooming_claws / Claws Grooming_claws Absent, 26 Mammary_glands / Multiple Pair, 27 Uterus / Bicornate Simplex, 28 Placenta / Epitheliochorial Hemochorial, 29 'Precociality (teeth at birth)' / More Less, 30 'SINE human Alu transpositions C7, C9, C12' / Absent Present ;

MATRIX

TUPAIOIDEA 000000000000000010001111000000

LEMUROIDEA 000000000000{0 1}00010200001100000

LORISOIDEA 000000000001000010200011100000

TARSIOIDEA 101100110012{0 1}01000011011100101

CEBOIDEA 101112111121211111{1 2}1111{0 1}{1 2}11101

CERCOPITH. 111112111122211111211110211111

HOMINOIDEA 111112111122211111211110211111

Darwinius ????0??10000111?1?2??0112?????

Notharctus ????00000000{1 2}111101000112?????

Catopithecus ????011101211111111?1?????????

;

END;

BEGIN ASSUMPTIONS;

TYPESET * UNTITLED = unord: 25, ord: 1 - 24 26 - 30;

EXSET * UNTITLED = ;

WTSET * UNTITLED = 1: 1 - 30 ;

END;

**Section 17. original matrix corrected-pluschar31-39.nex**

#NEXUS

BEGIN TAXA;

TITLE Taxa;

DIMENSIONS NTAX=8;

TAXLABELS

TUPAIOIDEA LEMUROIDEA LORISOIDEA TARSIOIDEA CEBOIDEA CERCOPITH. HOMINOIDEA Darwinius

;

END;

BEGIN CHARACTERS;

TITLE Character_Matrix;

DIMENSIONS NCHAR=39;

FORMAT DATATYPE = STANDARD GAP = - MISSING = ? SYMBOLS = " 0 1 2";

CHARSTATELABELS

1 Nose_and_upper_lip / Wet_cleft Dry_continuous, 2 Jacobsons_vomeronasal_organ / Present Absent, 3 Sphenoidal_recess / Substantial Reduced, 4 Eye / Reflecting '-' tapetum '-' lucida Retinal_fovea, 5 Brain_and_braincase / Rel_small Rel_large, 6 Olfactory_bulbs / Rel_large Intermediate Rel_small, 7 Blood_supply_to_brain / Complex Promontory_artery, 8 Cranial_rostrum / Rel_long Rel_short, 9 Mandibular_ramus / Shallow Deep, 10 Metopic_suture_uniting_frontals / Open Fused, 11 Postorbital_closure / None Partial Complete, 12 Ectotympanic / Free Lateral_wall Tubular, 13 Mandibular_symphysis / Open Partial Fused, 14 Incisors / Pointed_procumbent Vertical_spatulate, 15 Canines / Procumbent Vertical_interlocking, 16 Canine_dimorphism / Absent Present, 17 Upper_molars / Tritubercular Quadrate_hypocone, 18 'Premolar P/4 ' / Elongated Transverse_pad '-' mcd, 19 Paraconid_on_lower_molars / present reduced absent, 20 'Capitate (os magnum)' / Compressed Uncompressed, 21 Fibular_facet_on_astragalus / Sloping Rel_steep, 22 Pes / Tarsi_fulcr. Metatar_fulcr., 23 Mesocuneiform / Compressed Uncompressed, 24 Longest_toe_of_pes / Third Fourth, 25 Claws_or_grooming_claws / Claws Grooming_claws Absent, 26 Mammary_glands / Multiple Pair, 27 Uterus / Bicornate Simplex, 28 Placenta / Epitheliochorial Hemochorial, 29 'Precociality (teeth at birth)' / More Less, 30 'SINE human Alu transpositions C7, C9, C12' / Absent Present, 31 Flexor_fibularis_groove_positionon_astragalus / 'in-line with medial tibial facet' lateral_to_medial_tibial_facet, 32 Posterior_aspect_of_astragalar_trochlea / unexpanded expanded_into_shelf, 33 peroneal_tuberosity_on_mt1 / reduced enlarged, 34 depth_of_medial_tibial_facet / shallow deep, 35 hypoconulid_lobe_on_m3 / abbreviated 'well-developed', 36 Cuboid_facet_of_navicular_contact / only_ectocuneiform_facet 'both ecto- and mesocuneiform facet ', 37 divergence_of_big_toe / not_divergent moderate_divergence extreme_divergence, 38 'Orbit Size/Activity Pattern' / 'Large/Nocturnal' 'Moderate/Cathemeral' 'Small/Diurnal', 39 Tibial_medial_malleolus_rotation / No_rotation Slight_rotation Marked_rotation ;

MATRIX

TUPAIOIDEA 0000000000000000100011110000000000000{0 2}{0 1}

LEMUROIDEA 000000000000{0 1}000102000011000001111112{0 1 2}2

LORISOIDEA 0000000000010000102000111000001{0 1}1111202

TARSIOIDEA 101100110012{0 1}01000011011100101001110101

CEBOIDEA 101112111121211111{1 2}1111{0 1}{1 2}11101000000121

CERCOPITH. 1111121111222111112111102111110000{0 1}0121

HOMINOIDEA 111112111122211111211110211111000000121

Darwinius ????0??10000111?1?2??011{1 2}?????????1120?

;

END;

BEGIN ASSUMPTIONS;

TYPESET * UNTITLED = unord: 25, ord: 1 - 24 26 - 39;

EXSET * UNTITLED = ;

WTSET * UNTITLED = 1: 1 - 39 ;

END;

**Section 18. Original matrix corrected-pluschar31-39-noth-cato-added.nex**

#NEXUS

BEGIN TAXA;

TITLE Taxa;

DIMENSIONS NTAX=10;

TAXLABELS

TUPAIOIDEA LEMUROIDEA LORISOIDEA TARSIOIDEA CEBOIDEA CERCOPITH. HOMINOIDEA Darwinius Notharctus Catopithecus

;

END;

BEGIN CHARACTERS;

TITLE Character_Matrix;

DIMENSIONS NCHAR=39;

FORMAT DATATYPE = STANDARD GAP = - MISSING = ? SYMBOLS = " 0 1 2";

CHARSTATELABELS

1 Nose_and_upper_lip / Wet_cleft Dry_continuous, 2 Jacobsons_vomeronasal_organ / Present Absent, 3 Sphenoidal_recess / Substantial Reduced, 4 Eye / Reflecting '-' tapetum '-' lucida Retinal_fovea, 5 Brain_and_braincase / Rel_small Rel_large, 6 Olfactory_bulbs / Rel_large Intermediate Rel_small, 7 Blood_supply_to_brain / Complex Promontory_artery, 8 Cranial_rostrum / Rel_long Rel_short, 9 Mandibular_ramus / Shallow Deep, 10 Metopic_suture_uniting_frontals / Open Fused, 11 Postorbital_closure / None Partial Complete, 12 Ectotympanic / Free Lateral_wall Tubular, 13 Mandibular_symphysis / Open Partial Fused, 14 Incisors / Pointed_procumbent Vertical_spatulate, 15 Canines / Procumbent Vertical_interlocking, 16 Canine_dimorphism / Absent Present, 17 Upper_molars / Tritubercular Quadrate_hypocone, 18 'Premolar P/4 ' / Elongated Transverse_pad '-' mcd, 19 Paraconid_on_lower_molars / present reduced absent, 20 'Capitate (os magnum)' / Compressed Uncompressed, 21 Fibular_facet_on_astragalus / Sloping Rel_steep, 22 Pes / Tarsi_fulcr. Metatar_fulcr., 23 Mesocuneiform / Compressed Uncompressed, 24 Longest_toe_of_pes / Third Fourth, 25 Claws_or_grooming_claws / Claws Grooming_claws Absent, 26 Mammary_glands / Multiple Pair, 27 Uterus / Bicornate Simplex, 28 Placenta / Epitheliochorial Hemochorial, 29 'Precociality (teeth at birth)' / More Less, 30 'SINE human Alu transpositions C7, C9, C12' / Absent Present, 31 Flexor_fibularis_groove_positionon_astragalus / 'in-line with medial tibial facet' lateral_to_medial_tibial_facet, 32 Posterior_aspect_of_astragalar_trochlea / unexpanded expanded_into_shelf, 33 peroneal_tuberosity_on_mt1 / reduced enlarged, 34 depth_of_medial_tibial_facet / shallow deep, 35 hypoconulid_lobe_on_m3 / abbreviated 'well-developed', 36 Cuboid_facet_of_navicular_contact / only_ectocuneiform_facet 'both ecto- and mesocuneiform facet ', 37 divergence_of_big_toe / not_divergent moderate_divergence extreme_divergence, 38 'Orbit Size/Activity Pattern' / 'Large/Nocturnal' 'Moderate/Cathemeral' 'Small/Diurnal', 39 Tibial_medial_malleolus_rotation / no_rotation slight_rotation marked_rotation ;

MATRIX

TUPAIOIDEA 0000000000000000100011110000000000000{0 2}{0 1}

LEMUROIDEA 000000000000{0 1}000102000011000001111112{0 1 2}2

LORISOIDEA 0000000000010000102000111000001{0 1}1111202

TARSIOIDEA 101100110012{0 1}01000011011100101001110101

CEBOIDEA 101112111121211111{1 2}1111{0 1}{1 2}11101000000121

CERCOPITH. 1111121111222111112111102111110000{0 1}0121

HOMINOIDEA 111112111122211111211110211111000000121

Darwinius ????0??10000111?1?2??011{1 2}?????????1120?

Notharctus ????00000000{1 2}111101000111?????111111222

Catopithecus ????011101211111111?1?????????00000?12?

;

END;

BEGIN ASSUMPTIONS;

TYPESET * UNTITLED = unord: 25, ord: 1 - 24 26 - 39;

EXSET * UNTITLED = ;

WTSET * UNTITLED = 1: 1 - 39 ;

END;

**References**

S1. Gingerich PD, Franzen JL, Habersetzer J, Hurum JH, Smith BH (2010) *Darwinius*

*masillae* is a haplorhine- reply to Williams et al. (2010). J Hum Evol 59: 574-

579.

S2. Fleagle JG, Kay RF (1987) The phyletic position of the Parapithecidae. J Hum Evol

16: 483-532.

S3. Simons EL, Rasmussen DT (1996) Skull of *Catopithecus browni*, an early tertiary

catarrhine. Am J Phys Anthropol 100: 261-292.

S4. Kay RF (1975) The functional adaptations of primate molar teeth. Am J Phys

Anthropol 43: 297-326.

S5. Gingerich PD, Smith BH, Rosenberg K (1982) Allometric scaling in the dentition of

primates and prediction of body weight from tooth size in fossils. Am J Phys

Anthropol 58: 81-100.

S6. Conroy GC (1987) Problems of body-weight estimation in fossil primates. Int J

Primatol 8: 115-137.

S7. Simons EL (1971) Relationships of *Amphipithecus* and *Oligopithecus*. Nature 232:

489-491.

S8. Beard KC, Wang J (2004) The eosimiid primates (Anthropoidea) of the Heti

Formation, Yuanqu Basin, Shanxi and Henan Provinces, People's Republic of China. J Hum Evol 46: 401-432.

S9. Takai M., Shigehara N (2004) The Pondaung primates, enigmatic "possible

anthropoids" from the latest middle Eocene, Central Myanmar. In: Ross CF, Kay RF, editors. Anthropoid Origins: New Visions. New York: Kluwer Academic/Plenum Publishers. pp. 283-321.

S10. Franzen JL, Gingerich PD, Habersetzer J, Hurum JH, von Koenigswald W, Smith

BH (2009) Complete primate skeleton from the Middle Eocene of Messel in Germany: morphology and paleobiology. PLoS One 4: e5723. doi: 10.1371/journal.pone.0005723

S11. Fleagle JG (1999) Primate adaptation and evolution, second edition. San Diego:

Academic Press. 528 p.

S12. Ravosa MJ (1996) Mandibular form and function in North American and European

Adapidae and Omomyidae. J Morph 229: 171-190.

S13. Ravosa MJ (1991) Structural allometry of the prosimian mandibular corpus and

symphysis. J Hum Evol 20: 3-20.

S14. Swindler DR (2002) Primate Dentition. Cambridge: Cambridge University Press.

296 p.

S15. Williams BA, Kay RF, Kirk EC, Ross CF (2010) *Darwinius masillae* is a

stresirrhine- a reply to Franzen et al. (2009). J Hum Evol 59: 567-573.

S16. Boyer DM, Seiffert ER, Simons EL (2010) Astragalar morphology of *Afradapi*s, a

large adapiform primate from the earliest Late Eocene of Egypt. Am J Phys Anthropol 143: 383-402.

S17. Seiffert ER, Perry JMG, Simons EL, Boyer DM (2009) Convergent evolution of

anthropoid-like adaptations in Eocene adapiform primates. Nature 461: 1118-1121.

S18. Dagosto M (1990) Models for the origins of the anthropoid postcranium. J Hum

Evol 19: 121-139.

S19. Morton DJ (1924) [Evolution of the human foot II](http://onlinelibrary.wiley.com/doi/10.1002/ajpa.1330070114/abstract). Am J Phys Anthropol 7: 1–52.

S20. Maiolino S, Boyer DM, Rosenberger AL (accepted) Morphological correlates of

the grooming claw in distal phalanges of platyrrhines and other primates: a preliminary study. In: Rosenberger AL, editor. Evolutionary Morphology of New World Monkeys.

S21. Dagosto M, Gebo D L (1994) Postcranial anatomy and the origin of the

Anthropoidea. In: Fleagle JG, Kay RF, editors. Anthropoid Origins. New York: Plenum Press. pp. 567-593.

S22. Jacobs RL, Boyer DM, Patel BA (2009) Comparative functional morphology of

the primate peroneal process. J Hum Evol 57: 721-731.

S23. Patel BA, Boyer DM, Jacobs RL, Seiffert ER, Simons EL (2010) Anthropoid first

metatarsal from the late Eocene of Egypt. Am J Phys Anthropol S50: 186.

S24. Beard KC, Tong Y, Dawson MR, Wang J, Huang X (1996) Earliest complete

dentition of an anthropoid primate from the late middle Eocene of Shanxi Province, China. Science 272: 82-85.

S25. Dagosto M (1988) Implications of postcranial evidence for the origin of

Euprimates. J Hum Evol **17:** 35–56.

S26. Kay RF, Kirk EC (2000) Osteological evidence for the evolution of activity pattern

and visual acuity in primates. Am J Phys Anthropol 113: 235-262.

S27. Gregory WK (1920) On the structure and relations of *Notharctus* an American

Eocene primate. Mem Am Mus Nat His, new series 3: 49-243.

S28. Dagosto M (1985) The distal tibia of primates with special reference to the

Omomyidae. Int J Primatol 6: 45-75.
